# Supplementary material for: The immune contexture of primary central nervous system diffuse large B cell lymphoma associates with patient survival and specific cell signaling
Source: Theranostics. 2021 Jan 22;11(8):3565–79. doi: 10.7150/thno.54343 (PMC7914352; doi:10.7150/thno.54343)
Supplement: Supplementary file 1 — Supplementary figures and tables. [file thnov11p3565s1.pdf]

The immune contexture of primary central nervous system diffuse large B cell lymphoma associates with patient survival and specific cell signaling

Melissa Alame[1,2,3,4], Emmanuel Cornillot[1,3,4,7], Valère Cacheux[2,5,7], Valérie Rigau[5,6], Valérie Costes-Martineau[5,6], Vanessa Lacheretz-Szablewski[5,6,8], Jacques Colinge[1,4,5,8]

[1] Institut de Recherche en Cancérologie de Montpellier (IRCM), INSERM, Parc Euromédecine, 208 rue des Apothicaires, 34298 Montpellier, France
[2] Biological Hematology Department, CHU Montpellier, Hôpital Saint Eloi, 34275 Montpellier, France
[3] Université de Montpellier, Faculté de Pharmacie, 15 avenue Charles Flahault, 34093 Montpellier, France
[4] Institut régional du Cancer Montpellier (ICM), Parc Euromédecine, 208 rue des Apothicaires, 34298 Montpellier, France
[5] Université de Montpellier, Faculté de Médecine, 2 rue école de Médecine, 34060 Montpellier, France
[6] Département de pathologie et d'oncologie biologique, CHU Montpellier, Hôpital Gui De Chauliac, 34000 Montpellier, France
[7] Equal contribution
[8] Corresponding authors: jacques.colinge@inserm.fr, v-szablewski@chu-montpellier.fr

Supplementary Tables

Table S1. References and dilutions of primary antibodies used for IHC.

**Target**	**Manufacturer**	**Cat. No.**	**Dilution IHC**
CD20	Dako Denmark A/S	L26	1/300
CD3	Ventana, PREP Kit Ventana	2GV6	undiluted
CD5	Dako, Denmark A/S	4C7	1/100
CD4	Ventana PREP Kit Ventan	SP35	undiluted
CD8	Ventana, PREP Kit Ventana	SP57	undiluted
CD10	Menarini, California USA	56C6	1/10
BCL6	Ventana PREP Kit Ventana	G1191E/A8	undiluted
MUM1	Dako	MUM1p	1/50
P53	Ventana, PREP Kit Ventana	DO7	undiluted
MYC	Epitomics, Burlingame, CA, USA	EP 121	1/100
CD68	DAKO	KP1	1/400
CD163	Ventana, PREP Kit Ventana	MRQ-26	undiluted
KI67	Ventana, PREP Kit Ventana	30-9	undiluted
PD1	Abcam, Paris FRANCE	NAT105	1/100
PDL1	Cell Signalling Technology	E1L3M	1/200
TIM3	Cell Signalling Technology	D5D5R	1/100
Galectin-9	OriGene Technologies	OTI8B11	1/150

Tables S2 to S8 are compiled at the end of this document:

- **Table S2.** GO terms enriched in the 4 gene clusters
- **Table S3.** Pathways (GO terms and Reactome pathways) associated with HLA status in PCNSL
- **Table S4.** Pathways (GO terms and Reactome pathways) associated with HLA class I gene expression in PCNSL
- **Table S5.** GO terms associated with high *HLA-DRA* gene expression in PCNSL
- **Table S6.** L-R pairs selected by RNA-sequencing analysis (n=20)
- **Table S7.** L-R pairs selected by microarrays analysis (n=34)
- **Table S8.** L-R pairs selection

Supplementary Figures

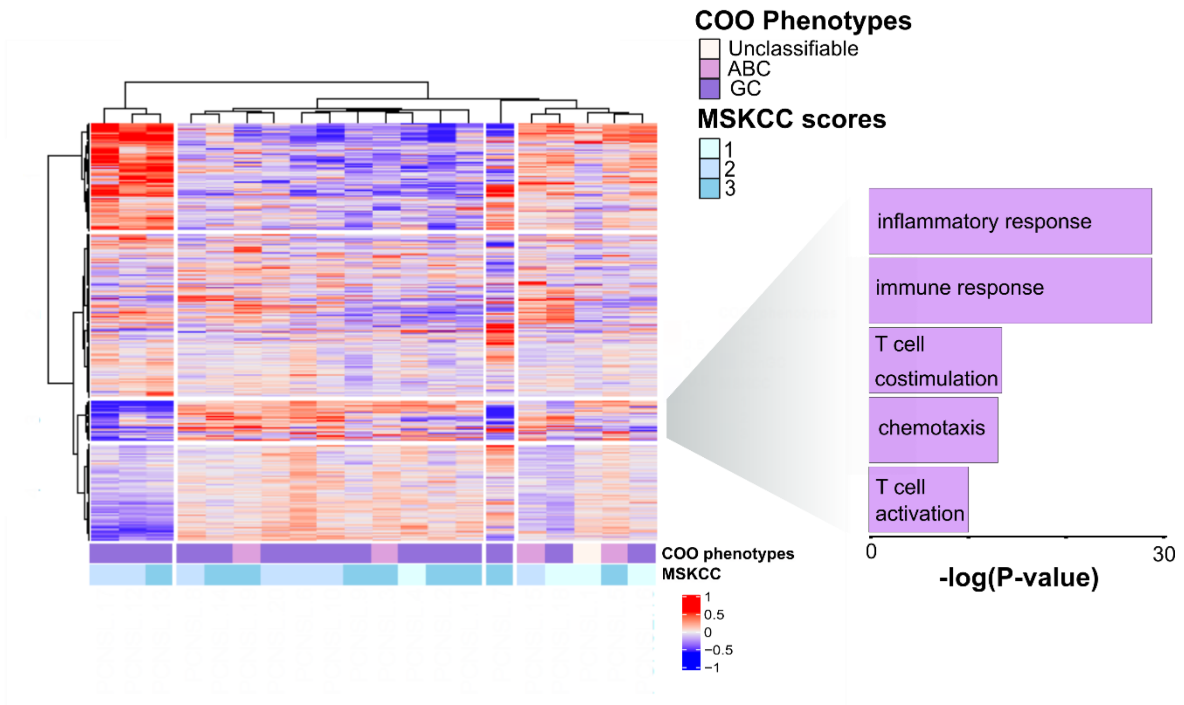

Figure S1. An immune gene expression cluster revealed heterogeneity among PCNSL samples.

Four clusters of genes were revealed by unsupervised clustering of our 20 PCNSL complete transcriptomes (Ward's method based on Euclidean distance). The main GOBP terms found enriched in cluster 3 were almost exclusively related to immune activation (hypergeometric test, FDR <0.01, at least 10 deregulated genes in each GO term to limit the size of the figure). The full list of GOBP terms is provided in Table S2. Cell of origin (COO) phenotypes: germinal center (GC), Activated B-Cell (ABC), and unclassifiable showed 75% (15/20) of PCNSL with ABC phenotype. Memorial Sloan Kettering Cancer Center Score (MSKCC) is a prognosis score used to evaluate the prognosis of PCNSL patients at the diagnosis time.

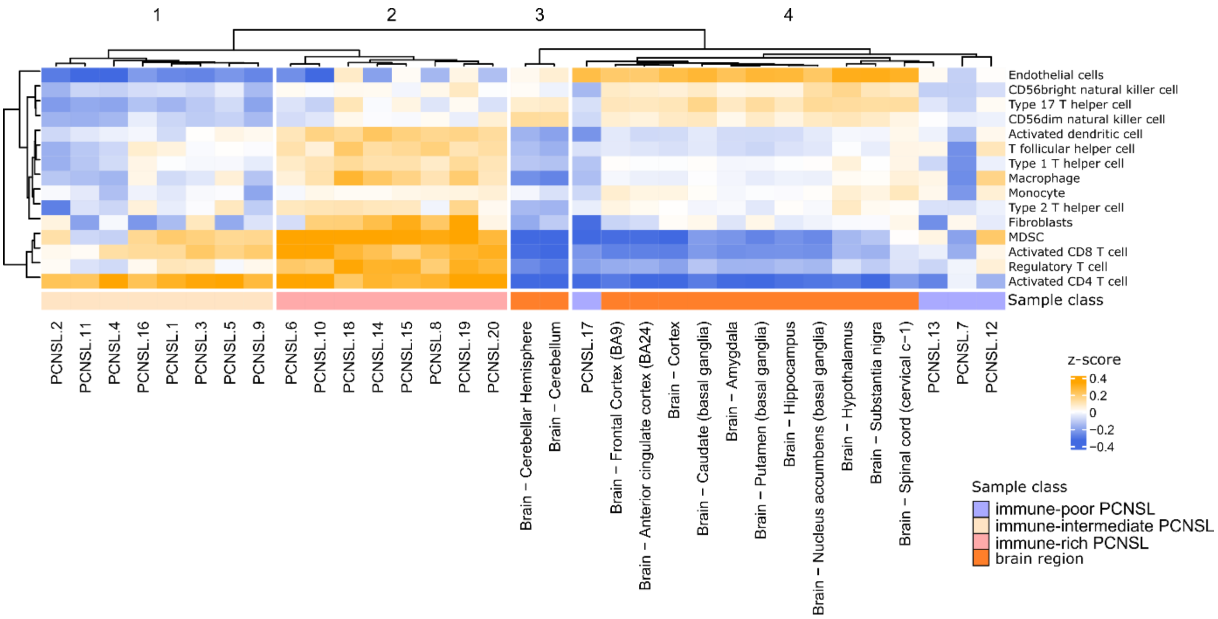

Figure S2. Comparison of brain region and PCNSL transcriptomes.

Clustering of normal brain tissues with PCNSL transcriptomes shows that immune-poor PCNSLs harbor an immune component comparable or reduced compared to normal brain. Immune-intermediate and immune-rich PCNSLs include a much enriched activated T cell component.

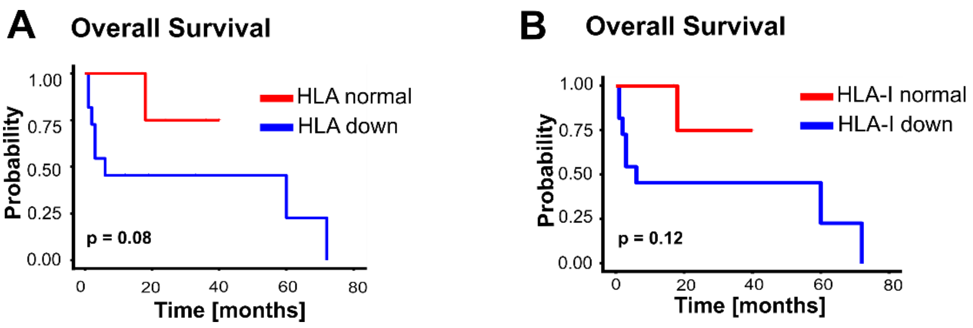

Figure S3. *HLA* gene expression and Overall Survival in PCNSL.

A. Overall Survival of PCNSL patients with respect to *HLA* gene expression (Kaplan Meier curves, log rank test, n = 14, normal = above the median, low = below the median).
B. Overall Survival of PCNSL patients with respect to HLA class I gene expression (Kaplan Meier curves, log rank test, n = 17, normal = above the median, low = below the median).

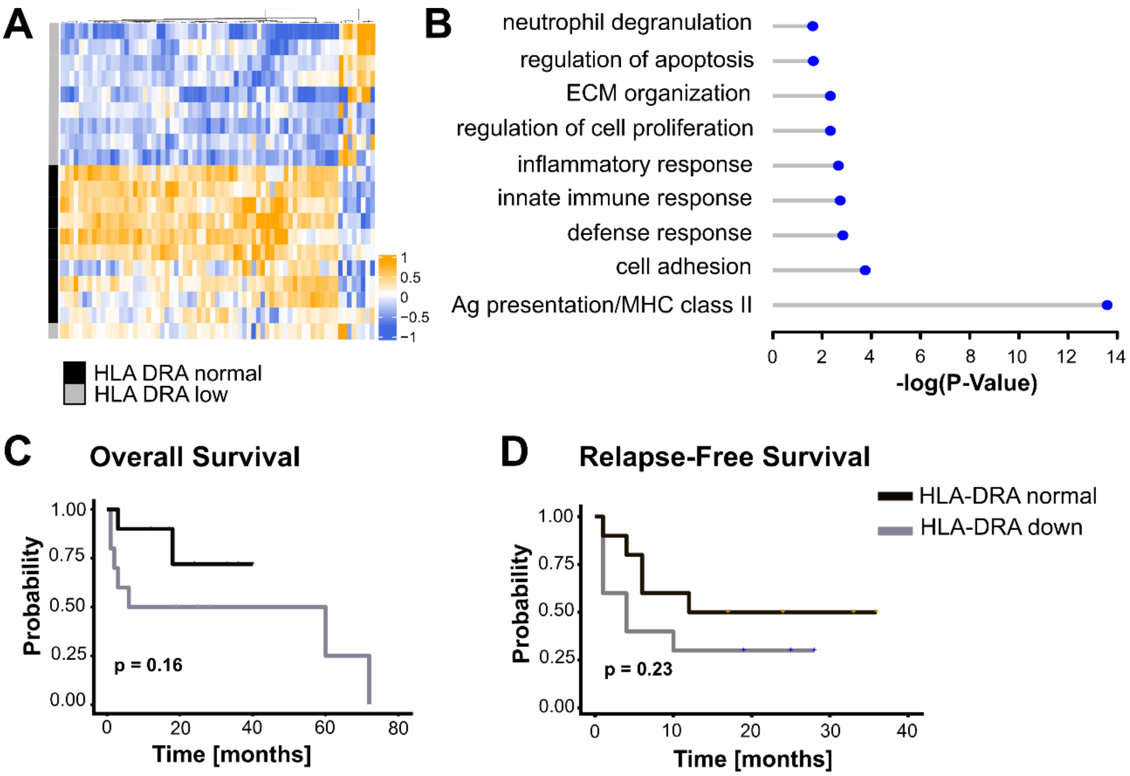

Figure S4. ***HLA-DRA*** **gene expression was not associated with cytotoxic T cells in PCNSL.**

A. Differentially expressed genes between PCNSL with normal *HLA-DRA* gene expression (light orange) and *HLA-DRA* gene expression down (purple): 69 significantly deregulated genes were selected (FDR <0.01, $\log_2$-FC>4 in absolute value, average read count >20), which segregated the two sample clusters perfectly.

B. Main GOBP terms found significantly enriched in genes in Fig. S3a (hypergeometric test, FDR <0.05, at least 3 deregulated genes in the GO term).

C. Overall Survival of PCNSL patients with respect to *HLA-DRA* gene expression (Kaplan-Meier curves, log-rank test, n = 20, normal = above the median, low = below the median).

D. Relapse Free Survival of PCNSL patients with respect to *HLA-DRA* gene expression (Kaplan-Meier curves, log-rank test, n = 20, normal= above the median, low = below the median).

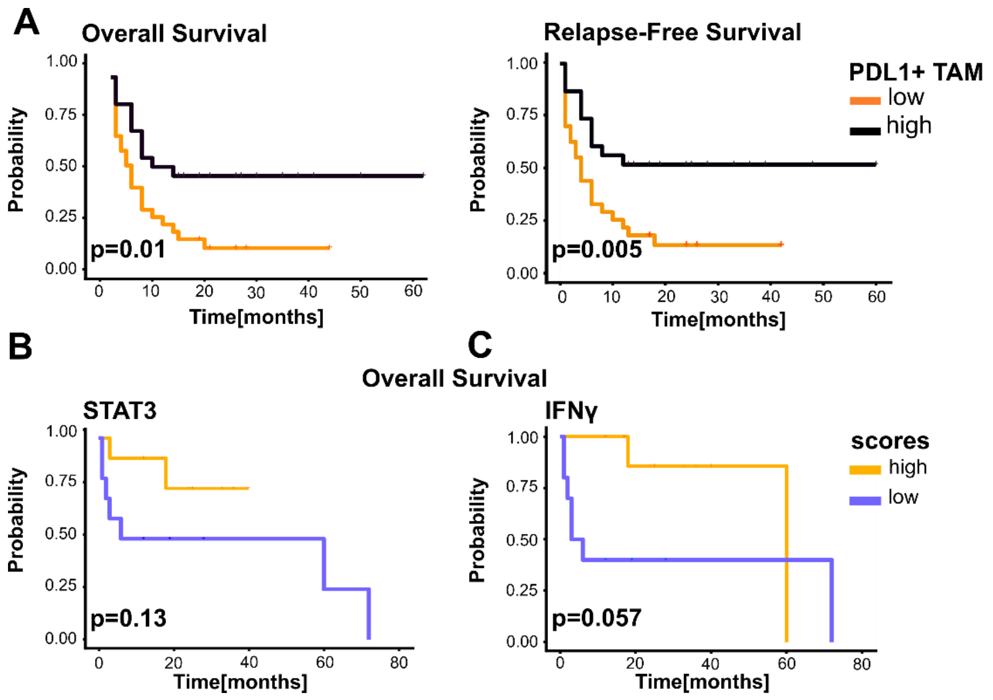

Figure S5 Immune signaling in PCNSL.

A. High PD-L1+TAMs was associated with a better outcome (Kaplan-Meier curves, log-rank test, n = 57). High TAM density (2-3) and a high percentage of CD68+ cells expressing PD-L1 protein (> 50% of CD68+ cells) define high PD-L1+ TAMs.
B. Overall Survival of PCNSL patients with respect to STAT3 gene signature score (Kaplan-Meier curves, log-rank test, n = 20, high = above the median, low = below the median).
C. Overall Survival of PCNSL patients with respect to IFNγ gene signature score (Kaplan-Meier curves, log-rank test, n = 20, high = above the median, low = below the median).

article title	**The immune landscapes of primary central nervous diffuse large B cell lymphoma**

author names

Melissa Alame[1,2,3,4], Emmanuel Cornillot[1,3,4,7], Valère Cacheux[2,5,7], Valérie Rigau[5,6], Valérie Costes-Martineau[5,6], Vanessa Lacheretz-Szablewski[5,6,8], Jacques Colinge[1,4,5,8]

affiliation

1 Institut de Recherche en Cancérologie de Montpellier (IRCM), INSERM, Parc Euromédecine, 208 rue des Apothicaires, 34298 Montpellier, France
2 Biological Hematology Department, CHU Montpellier, Hôpital Saint Eloi, 34275 Montpellier, France
3 Université de Montpellier, Faculté de Pharmacie, 15 avenue Charles Flahault, 34093 Montpellier, France
4 Institut régional du Cancer Montpellier (ICM), Parc Euromédecine, 208 rue des Apothicaires, 34298 Montpellier, France
5 Université de Montpellier, Faculté de Médecine, 2 rue école de Médecine, 34060 Montpellier, France
6 Département de pathologie et d'oncologie biologique, CHU Montpellier, Hôpital Gui De Chauliac, 34000 Montpellier, France
7 Equal contribution
8 Corresponding authors: jacques.colinge@inserm.fr, v-szablewski@chu-montpellier.fr

Description of column titles for Supplementary Tables S2-S5

term	either GO or Reactome term ID
description	description of either GO or Reactome term
intersect.size	number of transcripts/proteins which is found in the GO/Reactome term
n.pw	number of transcripts/proteins in the term
FDR	False Discovery Rate

Table S2 GO terms enriched in the 4 gene clusters identified by whole transcriptomic analysis

gene clusters	GO term	GO description	intersect genes number	n.pw	adjusted p-values
C.1	GO:0071805	potassium ion transmembrane transport	65	133	1.09099591964227e-05
C.1	GO:0034220	ion transmembrane transport	93	235	0.00104997827969927
C.1	GO:0007155	cell adhesion	198	465	2.59408494684976e-11
C.1	GO:0007612	learning	31	58	0.00136013922939981
C.1	GO:0007409	axonogenesis	69	104	0
C.1	GO:0035725	sodium ion transmembrane transport	35	75	0.00998585007199703
C.1	GO:0007399	nervous system development	186	328	0
C.1	GO:0001764	neuron migration	48	103	0.00113605772858566
C.1	GO:0007411	axon guidance	91	193	2.77787197799917e-07
C.1	GO:0051056	regulation of small GTPase mediated signal transduction	58	141	0.00891488849550136
C.1	GO:0007420	brain development	84	201	0.000329561784690646
C.1	GO:0007268	chemical synaptic transmission	131	265	1.17331144799948e-12
C.1	GO:0007156	homophilic cell adhesion via plasma membrane adhesion molecules	101	157	0
C.1	GO:0007267	cell-cell signaling	126	317	5.28559230813519e-05
C.1	GO:0007417	central nervous system development	60	118	6.31313766743524e-06
C.1	GO:0007605	sensory perception of sound	58	140	0.00719667649634692
C.1	GO:0017158	regulation of calcium ion-dependent exocytosis	20	33	0.00356236722370917
C.1	GO:0048791	calcium ion-regulated exocytosis of neurotransmitter	23	36	0.000423369429762547
C.1	GO:1902476	chloride transmembrane transport	43	87	0.000608723771557573
C.1	GO:0007214	gamma-aminobutyric acid signaling pathway	17	25	0.00171467804516548
C.1	GO:0034765	regulation of ion transmembrane transport	51	117	0.00431974077370463
C.1	GO:2000311	regulation of AMPA receptor activity	16	20	0.000199435746624588
C.1	GO:0006813	potassium ion transport	40	76	0.000217032946443051
C.1	GO:1903779	regulation of cardiac conduction	31	56	0.000644209864500028
C.1	GO:0061337	cardiac conduction	28	44	7.19523778213382e-05
C.1	GO:0016339	calcium-dependent cell-cell adhesion via plasma membrane cell adhesion molecules	32	45	2.75073375827217e-07
C.1	GO:0070588	calcium ion transmembrane transport	50	118	0.00994485741188963
C.1	GO:0042391	regulation of membrane potential	55	116	0.000217032946443051
C.1	GO:0014047	glutamate secretion	18	28	0.00286911946111348
C.1	GO:0051965	positive regulation of synapse assembly	42	63	1.791869053136e-08
C.1	GO:0001523	retinoid metabolic process	30	61	0.00955616241722268
C.1	GO:0050808	synapse organization	25	36	2.92996264820924e-05
C.1	GO:0007269	neurotransmitter secretion	30	49	8.2339650132451e-05
C.1	GO:2000463	positive regulation of excitatory postsynaptic potential	18	24	0.000199435746624588
C.1	GO:0007158	neuron cell-cell adhesion	13	16	0.00095310974960499
C.1	GO:0048813	dendrite morphogenesis	22	36	0.00144635673243358

C.1	GO:0016079	synaptic vesicle exocytosis	26	44	0.000717582409895212
C.1	GO:0035249	synaptic transmission, glutamatergic	20	31	0.00117666327632557
C.1	GO:0007416	synapse assembly	44	64	1.38799188809102e-09
C.1	GO:0030534	adult behavior	19	26	0.000199435746624588
C.1	GO:0046037	GMP metabolic process	12	16	0.00567908471676388
C.1	GO:0046710	GDP metabolic process	10	12	0.00526181066756294
C.1	GO:0043410	positive regulation of MAPK cascade	64	143	0.00033472458303363
C.1	GO:0035235	ionotropic glutamate receptor signaling pathway	17	23	0.000423369429762547
C.1	GO:0060291	long-term synaptic potentiation	27	45	0.000376711978586473
C.1	GO:0042552	myelination	25	46	0.00482587018554759
C.1	GO:0007215	glutamate receptor signaling pathway	14	15	2.92996264820924e-05
C.1	GO:0086091	regulation of heart rate by cardiac conduction	21	36	0.00477345625616437
C.1	GO:0048169	regulation of long-term neuronal synaptic plasticity	14	21	0.00982710025415061
C.1	GO:0050771	negative regulation of axonogenesis	15	21	0.00214440039082751
C.1	GO:0071625	vocalization behavior	13	14	8.2339650132451e-05
C.1	GO:0048712	negative regulation of astrocyte differentiation	9	10	0.00431755875317672
C.1	GO:0033674	positive regulation of kinase activity	33	69	0.00915380416912465
C.1	GO:0050804	modulation of chemical synaptic transmission	21	34	0.00170659463951223
C.1	GO:0060078	regulation of postsynaptic membrane potential	12	15	0.00223483792738766
C.1	GO:0022010	central nervous system myelination	10	11	0.00148964435532022
C.1	GO:0016337	cell-cell adhesion	18	26	0.000840555246860836
C.3	GO:0006955	immune response	135	450	0
C.3	GO:0002250	adaptive immune response	71	255	2.10849856093394e-13
C.3	GO:0050776	regulation of immune response	63	208	1.26509913656037e-13
C.3	GO:0050900	leukocyte migration	43	206	9.90169434663203e-05
C.3	GO:0045087	innate immune response	103	516	8.05994659902609e-10
C.3	GO:0050853	B cell receptor signaling pathway	24	114	0.00586921455186801
C.3	GO:0010469	regulation of signaling receptor activity	86	442	1.09393298352168e-07
C.3	GO:0007169	transmembrane receptor protein tyrosine kinase signaling pathway	30	105	5.48452762610258e-06
C.3	GO:0002548	monocyte chemotaxis	14	43	0.00129180693741074
C.3	GO:0006954	inflammatory response	136	392	0
C.3	GO:0030593	neutrophil chemotaxis	27	64	2.48207985537368e-09
C.3	GO:0048247	lymphocyte chemotaxis	15	32	6.85669453022492e-06
C.3	GO:0070098	chemokine-mediated signaling pathway	36	71	0
C.3	GO:0070374	positive regulation of ERK1 and ERK2 cascade	42	230	0.0021581610176062
C.3	GO:0071346	cellular response to interferon-gamma	29	89	4.03714069524347e-07
C.3	GO:0071347	cellular response to interleukin-1	28	76	3.70596415928807e-08
C.3	GO:0071356	cellular response to tumor necrosis factor	33	123	6.41257644335038e-06
C.3	GO:0001816	cytokine production	13	27	2.59057176593617e-05
C.3	GO:0007155	cell adhesion	79	465	8.03285555415201e-05
C.3	GO:0007165	signal transduction	188	1211	3.70596415928807e-08
C.3	GO:0016554	cytidine to uridine editing	8	12	0.000130016643563561
C.3	GO:0008283	cell proliferation	57	367	0.00826863464191963

C.3	GO:0030154	cell differentiation	88	621	0.00806153019385363
C.3	GO:0097190	apoptotic signaling pathway	20	76	0.00117605721784402
C.3	GO:0071222	cellular response to lipopolysaccharide	46	131	1.92927618325456e-12
C.3	GO:0007166	cell surface receptor signaling pathway	62	259	6.3050291901037e-09
C.3	GO:0022617	extracellular matrix disassembly	22	80	0.000280180981133579
C.3	GO:0033089	positive regulation of T cell differentiation in thymus	6	10	0.0031738797078722
C.3	GO:0033209	tumor necrosis factor-mediated signaling pathway	31	126	7.6587247744043e-05
C.3	GO:0050852	T cell receptor signaling pathway	42	174	3.62395445575556e-06
C.3	GO:0007267	cell-cell signaling	59	317	8.66554346541658e-05
C.3	GO:0042102	positive regulation of T cell proliferation	30	60	1.62655603272047e-12
C.3	GO:0042832	defense response to protozoan	11	19	1.89895961743384e-05
C.3	GO:0050870	positive regulation of T cell activation	8	18	0.00354660006508155
C.3	GO:0006915	apoptotic process	90	541	4.38748261737676e-05
C.3	GO:0032496	response to lipopolysaccharide	52	167	6.99254795480639e-12
C.3	GO:0009615	response to virus	29	106	1.89895961743384e-05
C.3	GO:0019221	cytokine-mediated signaling pathway	81	311	1.51811896387244e-13
C.3	GO:0006952	defense response	21	65	3.62370401066227e-05
C.3	GO:0050728	negative regulation of inflammatory response	27	94	1.72086772327634e-05
C.3	GO:0030574	collagen catabolic process	31	65	2.3896317023918e-12
C.3	GO:0010529	negative regulation of transposition	8	18	0.00354660006508155
C.3	GO:0043312	neutrophil degranulation	98	481	8.05994659902609e-10
C.3	GO:0043123	positive regulation of I-kappaB kinase/NF-kappaB signaling	34	167	0.00127772699006555
C.3	GO:0000070	mitotic sister chromatid segregation	11	32	0.00354660006508155
C.3	GO:0050729	positive regulation of inflammatory response	18	70	0.00298279198835079
C.3	GO:0007229	integrin-mediated signaling pathway	20	88	0.00623692635073399
C.3	GO:0051603	proteolysis involved in cellular protein catabolic process	11	33	0.00444292363281422
C.3	GO:0051607	defense response to virus	42	181	9.90293181991109e-06
C.3	GO:0007062	sister chromatid cohesion	23	108	0.00639994857247511
C.3	GO:0019886	antigen processing and presentation of exogenous peptide antigen via MHC class II	24	98	0.000822049942814453
C.3	GO:0042127	regulation of cell proliferation	48	197	3.52248247886395e-07
C.3	GO:0030183	B cell differentiation	17	66	0.00381387748785184
C.3	GO:0051897	positive regulation of protein kinase B signaling	29	147	0.00511917255148888
C.3	GO:0006935	chemotaxis	43	123	1.1681082027574e-11
C.3	GO:0045766	positive regulation of angiogenesis	26	130	0.00731694541134059
C.3	GO:0048245	eosinophil chemotaxis	9	25	0.00731694541134059
C.3	GO:0032689	negative regulation of interferon-gamma production	10	25	0.00191761715988762
C.3	GO:0032720	negative regulation of tumor necrosis factor production	12	39	0.00525012985419082

C.3	GO:0032753	positive regulation of interleukin-4 production	8	20	0.00709741833621589
C.3	GO:0050718	positive regulation of interleukin-1 beta secretion	13	26	1.70662066725338e-05
C.3	GO:1902715	positive regulation of interferon-gamma secretion	5	7	0.00354660006508155
C.3	GO:0032729	positive regulation of interferon-gamma production	22	43	1.72777751827891e-09
C.3	GO:0032733	positive regulation of interleukin-10 production	11	24	0.000255832282948653
C.3	GO:0032735	positive regulation of interleukin-12 production	12	25	6.60320353973802e-05
C.3	GO:0032760	positive regulation of tumor necrosis factor production	14	46	0.0023833457526182
C.3	GO:0042088	T-helper 1 type immune response	5	8	0.00711385990911252
C.3	GO:0045785	positive regulation of cell adhesion	13	43	0.00381387748785184
C.3	GO:0097191	extrinsic apoptotic signaling pathway	13	43	0.00381387748785184
C.3	GO:0030168	platelet activation	26	111	0.000882439608197645
C.3	GO:0000278	mitotic cell cycle	27	123	0.0017203707343926
C.3	GO:0030198	extracellular matrix organization	51	197	1.49546609873319e-08
C.3	GO:0010818	T cell chemotaxis	6	11	0.00525012985419082
C.3	GO:0030101	natural killer cell activation	12	21	7.75679435086977e-06
C.3	GO:0030217	T cell differentiation	14	33	5.79924435703294e-05
C.3	GO:0042110	T cell activation	21	42	7.53751392295873e-09
C.3	GO:0042113	B cell activation	12	26	9.97136518885912e-05
C.3	GO:0043303	mast cell degranulation	7	14	0.0035483765482586
C.3	GO:0001525	angiogenesis	43	229	0.00110689802547591
C.3	GO:0032693	negative regulation of interleukin-10 production	9	13	2.5435392633565e-05
C.3	GO:0007204	positive regulation of cytosolic calcium ion concentration	38	137	3.37567293238195e-07
C.3	GO:0031295	T cell costimulation	33	75	5.56643620086561e-12
C.3	GO:0090023	positive regulation of neutrophil chemotaxis	12	30	0.000510927998759429
C.3	GO:2000406	positive regulation of T cell migration	7	14	0.0035483765482586
C.3	GO:0060333	interferon-gamma-mediated signaling pathway	29	71	1.33783390291834e-09
C.3	GO:0009967	positive regulation of signal transduction	19	59	0.000101622319399352
C.3	GO:0038111	interleukin-7-mediated signaling pathway	8	19	0.00504300461538698
C.3	GO:0042531	positive regulation of tyrosine phosphorylation of STAT protein	23	63	1.23363515955877e-06
C.3	GO:0050868	negative regulation of T cell activation	6	12	0.00826460604713744
C.3	GO:0002690	positive regulation of leukocyte chemotaxis	10	23	0.000975853762221081
C.3	GO:0002526	acute inflammatory response	8	17	0.0023833457526182
C.3	GO:0002544	chronic inflammatory response	5	8	0.00711385990911252
C.3	GO:0019722	calcium-mediated signaling	16	56	0.00196644536841997
C.3	GO:0019835	cytolysis	9	21	0.00224815473097649
C.3	GO:0030889	negative regulation of B cell proliferation	9	16	0.000218120345079233
C.3	GO:0002230	positive regulation of defense response to virus by host	12	24	4.10014854888055e-05
C.3	GO:0060337	type I interferon signaling pathway	17	66	0.00381387748785184

C.3	GO:0045954	positive regulation of natural killer cell mediated cytotoxicity	10	19	0.000146821185176453
C.3	GO:0030199	collagen fibril organization	13	42	0.00329240151635672
C.3	GO:0043372	positive regulation of CD4-positive, alpha-beta T cell differentiation	5	6	0.00143164197188873
C.3	GO:0034341	response to interferon-gamma	10	24	0.00141417141308821
C.3	GO:0043011	myeloid dendritic cell differentiation	8	18	0.00354660006508155
C.3	GO:0006569	tryptophan catabolic process	6	9	0.00159885561158063
C.3	GO:0019441	tryptophan catabolic process to kynurenine	5	7	0.00354660006508155
C.3	GO:0042771	intrinsic apoptotic signaling pathway in response to DNA damage by p53 class mediator	10	29	0.0058439220266263
C.3	GO:0043029	T cell homeostasis	10	28	0.00444292363281422
C.3	GO:0045589	regulation of regulatory T cell differentiation	10	15	1.11802378465724e-05
C.3	GO:0050727	regulation of inflammatory response	21	79	0.000724377506097224
C.3	GO:0001916	positive regulation of T cell mediated cytotoxicity	6	12	0.00826460604713744
C.3	GO:0045078	positive regulation of interferon-gamma biosynthetic process	6	12	0.00826460604713744
C.3	GO:0001823	mesonephros development	6	11	0.00525012985419082
C.3	GO:0043304	regulation of mast cell degranulation	6	10	0.0031738797078722
C.3	GO:0032695	negative regulation of interleukin-12 production	8	12	0.000130016643563561
C.3	GO:0046641	positive regulation of alpha-beta T cell proliferation	5	8	0.00711385990911252
C.3	GO:0006959	humoral immune response	22	57	7.89635619343639e-07
C.3	GO:0006968	cellular defense response	22	57	7.89635619343639e-07
C.3	GO:0002467	germinal center formation	6	8	0.000724377506097224
C.3	GO:0038083	peptidyl-tyrosine autophosphorylation	18	48	1.94277646567986e-05
C.3	GO:0042104	positive regulation of activated T cell proliferation	10	23	0.000975853762221081
C.3	GO:0002430	complement receptor mediated signaling pathway	6	12	0.00826460604713744
C.3	GO:0042130	negative regulation of T cell proliferation	15	39	9.83010307523377e-05
C.3	GO:0030890	positive regulation of B cell proliferation	19	41	2.52009255579296e-07
C.3	GO:0032609	interferon-gamma production	6	8	0.000724377506097224
C.3	GO:0035655	interleukin-18-mediated signaling pathway	5	7	0.00354660006508155
C.3	GO:0050892	intestinal absorption	7	14	0.0035483765482586
C.3	GO:0050715	positive regulation of cytokine secretion	9	25	0.00731694541134059
C.3	GO:0016064	immunoglobulin mediated immune response	7	11	0.000724377506097224
C.3	GO:0010536	positive regulation of activation of Janus kinase activity	5	6	0.00143164197188873
C.3	GO:2000427	positive regulation of apoptotic cell clearance	5	6	0.00143164197188873
C.3	GO:0032740	positive regulation of interleukin-17 production	8	13	0.000280180981133579
C.3	GO:0050901	leukocyte tethering or rolling	10	17	4.64750434511785e-05
C.3	GO:0045086	positive regulation of interleukin-2 biosynthetic process	6	12	0.00826460604713744
C.3	GO:0038110	interleukin-2-mediated signaling pathway	6	11	0.00525012985419082

C.3	GO:0050777	negative regulation of immune response	14	28	6.7771615253718e-06
C.3	GO:0001817	regulation of cytokine production	16	50	0.000572015140696464
C.3	GO:0042476	odontogenesis	10	31	0.00918394808055539
C.3	GO:0002523	leukocyte migration involved in inflammatory response	6	9	0.00159885561158063
C.3	GO:0007159	leukocyte cell-cell adhesion	10	26	0.00258944723324042
C.3	GO:0002687	positive regulation of leukocyte migration	7	12	0.00138364572663393
C.3	GO:0001915	negative regulation of T cell mediated cytotoxicity	4	5	0.00731694541134059
C.3	GO:0002819	regulation of adaptive immune response	5	8	0.00711385990911252
C.3	GO:0070233	negative regulation of T cell apoptotic process	5	8	0.00711385990911252
C.3	GO:0043382	positive regulation of memory T cell differentiation	5	6	0.00143164197188873
C.3	GO:0033004	negative regulation of mast cell activation	5	5	0.000341868961463913
C.3	GO:0050869	negative regulation of B cell activation	6	7	0.000232592507448836
C.3	GO:0038114	interleukin-21-mediated signaling pathway	6	8	0.000724377506097224
C.3	GO:0042509	regulation of tyrosine phosphorylation of STAT protein	4	5	0.00731694541134059
C.3	GO:0070383	DNA cytosine deamination	6	9	0.00159885561158063
C.3	GO:0045869	negative regulation of single stranded viral RNA replication via double stranded DNA intermediate	6	9	0.00159885561158063
C.4	GO:0042274	ribosomal small subunit biogenesis	15	20	6.07264961973356e-05
C.4	GO:0006457	protein folding	87	190	1.86155723375896e-09
C.4	GO:0006355	regulation of transcription, DNA-templated	330	983	1.17840187103207e-10
C.4	GO:0000731	DNA synthesis involved in DNA repair	24	37	5.31656963376827e-06
C.4	GO:0006261	DNA-dependent DNA replication	17	26	0.000206614330427486
C.4	GO:0006357	regulation of transcription by RNA polymerase II	366	1218	1.00137252358925e-05
C.4	GO:0016567	protein ubiquitination	150	488	0.00503954781400909
C.4	GO:0006412	translation	140	188	0
C.4	GO:0000027	ribosomal large subunit assembly	21	27	2.48256481877935e-07
C.4	GO:0000028	ribosomal small subunit assembly	13	19	0.00101661753482992
C.4	GO:0000462	maturation of SSU-rRNA from tricistronic rRNA transcript (SSU-rRNA, 5.8S rRNA, LSU-rRNA)	29	32	2.4034414305506e-13
C.4	GO:0006383	transcription by RNA polymerase III	21	34	7.78824579942767e-05
C.4	GO:0000077	DNA damage checkpoint	18	31	0.000987185501811267
C.4	GO:0000956	nuclear-transcribed mRNA catabolic process	10	15	0.00827507259825226
C.4	GO:0006397	mRNA processing	96	172	0
C.4	GO:0006351	transcription, DNA-templated	562	1744	1.39399602971935e-14
C.4	GO:0006367	transcription initiation from RNA polymerase II promoter	70	170	1.59682662290929e-05
C.4	GO:0000209	protein polyubiquitination	93	242	1.12592599188101e-05
C.4	GO:0030433	ubiquitin-dependent ERAD pathway	32	63	9.16982180568993e-05
C.4	GO:0006977	DNA damage response, signal transduction by p53 class mediator resulting in cell cycle arrest	31	62	0.000182490241672998
C.4	GO:0006511	ubiquitin-dependent protein catabolic process	87	220	6.35608262428391e-06

C.4	GO:0006974	cellular response to DNA damage stimulus	107	218	6.70190398903532e-14
C.4	GO:0090305	nucleic acid phosphodiester bond hydrolysis	36	78	0.000333356552029397
C.4	GO:0032981	mitochondrial respiratory chain complex I assembly	28	64	0.00602977887616229
C.4	GO:0051301	cell division	141	355	1.28926562430136e-09
C.4	GO:0010501	RNA secondary structure unwinding	32	46	4.26388535590405e-09
C.4	GO:0048026	positive regulation of mRNA splicing, via spliceosome	11	17	0.0064029125581949
C.4	GO:0006302	double-strand break repair	32	62	6.2629112274567e-05
C.4	GO:0006400	tRNA modification	19	30	0.000128859559171066
C.4	GO:0090090	negative regulation of canonical Wnt signaling pathway	61	169	0.00423043377872259
C.4	GO:0042254	ribosome biogenesis	22	30	6.60231131129136e-07
C.4	GO:0006298	mismatch repair	20	32	0.000100052625953205
C.4	GO:0031297	replication fork processing	18	30	0.000561892471407549
C.4	GO:0075733	intracellular transport of virus	35	54	1.10157163677532e-08
C.4	GO:0002223	stimulatory C-type lectin receptor signaling pathway	57	122	1.27059126787954e-06
C.4	GO:0002479	antigen processing and presentation of exogenous peptide antigen via MHC class I, TAP-dependent	43	75	2.87318739406411e-08
C.4	GO:0006521	regulation of cellular amino acid metabolic process	38	51	3.37224039542401e-12
C.4	GO:0010972	negative regulation of G2/M transition of mitotic cell cycle	40	59	9.08934039145493e-11
C.4	GO:0016579	protein deubiquitination	117	271	1.40925254182239e-10
C.4	GO:0031145	anaphase-promoting complex-dependent catabolic process	55	80	0
C.4	GO:0031146	SCF-dependent proteasomal ubiquitin-dependent protein catabolic process	43	73	9.56468304188126e-09
C.4	GO:0033209	tumor necrosis factor-mediated signaling pathway	51	126	0.00058620048715004
C.4	GO:0038061	NIK/NF-kappaB signaling	46	68	2.7457497555078e-12
C.4	GO:0043161	proteasome-mediated ubiquitin-dependent protein catabolic process	99	223	8.74213176794681e-10
C.4	GO:0043488	regulation of mRNA stability	72	109	0
C.4	GO:0050852	T cell receptor signaling pathway	68	174	0.000148659104117649
C.4	GO:0060071	Wnt signaling pathway, planar cell polarity pathway	42	93	0.000144419016859058
C.4	GO:0061418	regulation of transcription from RNA polymerase II promoter in response to hypoxia	48	74	8.11228245072786e-12
C.4	GO:0070498	interleukin-1-mediated signaling pathway	54	96	6.94523671906921e-10
C.4	GO:0090263	positive regulation of canonical Wnt signaling pathway	54	139	0.00124314547385398

C.4	GO:1902036	regulation of hematopoietic stem cell differentiation	44	74	4.01118689141137e-09
C.4	GO:0000289	nuclear-transcribed mRNA poly(A) tail shortening	20	30	2.73210933401491e-05
C.4	GO:0017148	negative regulation of translation	33	71	0.00057911333114261
C.4	GO:0061014	positive regulation of mRNA catabolic process	9	11	0.00158721536168761
C.4	GO:0090503	RNA phosphodiester bond hydrolysis, exonucleolytic	22	31	1.63138075568929e-06
C.4	GO:0006270	DNA replication initiation	20	36	0.000919974158369619
C.4	GO:0007049	cell cycle	102	267	4.6174984380557e-06
C.4	GO:0008380	RNA splicing	111	169	0
C.4	GO:0006405	RNA export from nucleus	52	64	0
C.4	GO:0006606	protein import into nucleus	34	61	3.59168467721836e-06
C.4	GO:0000381	regulation of alternative mRNA splicing, via spliceosome	27	48	4.72917052952133e-05
C.4	GO:0006368	transcription elongation from RNA polymerase II promoter	60	103	8.15676055227672e-12
C.4	GO:0006378	mRNA polyadenylation	21	30	4.6174984380557e-06
C.4	GO:0006413	translational initiation	112	134	0
C.4	GO:0006417	regulation of translation	30	58	0.000112006517442051
C.4	GO:0000398	mRNA splicing, via spliceosome	192	244	0
C.4	GO:0016032	viral process	162	334	0
C.4	GO:0006110	regulation of glycolytic process	31	37	2.55928958581286e-12
C.4	GO:0006406	mRNA export from nucleus	80	98	0
C.4	GO:0006409	tRNA export from nucleus	29	33	1.39399602971935e-12
C.4	GO:0007077	mitotic nuclear envelope disassembly	30	44	3.13730561827992e-08
C.4	GO:0016925	protein sumoylation	47	76	1.51301601682201e-10
C.4	GO:0019083	viral transcription	100	108	0
C.4	GO:0060964	regulation of gene silencing by miRNA	40	56	7.33839338502256e-12
C.4	GO:1900034	regulation of cellular response to heat	51	76	2.24035076204895e-13
C.4	GO:0000086	G2/M transition of mitotic cell cycle	56	130	3.69965705680305e-05
C.4	GO:0010389	regulation of G2/M transition of mitotic cell cycle	39	82	7.09913499890044e-05
C.4	GO:0097711	ciliary basal body-plasma membrane docking	48	96	1.08503042766162e-06
C.4	GO:0032508	DNA duplex unwinding	30	43	1.37845629302614e-08
C.4	GO:0034080	CENP-A containing nucleosome assembly	17	30	0.00215757133153515
C.4	GO:0036297	interstrand cross-link repair	26	49	0.000242844208900609
C.4	GO:0006479	protein methylation	23	37	2.67111530206576e-05
C.4	GO:0007098	centrosome cycle	21	40	0.00161644021079509
C.4	GO:0006310	DNA recombination	36	73	6.23709331632377e-05
C.4	GO:0000387	spliceosomal snRNP assembly	25	28	3.37959037449032e-11
C.4	GO:0032968	positive regulation of transcription elongation from RNA polymerase II promoter	12	17	0.00123877450316662
C.4	GO:0007005	mitochondrion organization	39	91	0.00106539886245975
C.4	GO:0000290	deadenylation-dependent decapping of nuclear-transcribed mRNA	9	10	0.000441040283702821

C.4	GO:0033962	cytoplasmic mRNA processing body assembly	10	13	0.00157519962246477
C.4	GO:0006284	base-excision repair	20	37	0.00144145727420984
C.4	GO:0006099	tricarboxylic acid cycle	17	33	0.0083367977766903
C.4	GO:0000723	telomere maintenance	26	40	1.63138075568929e-06
C.4	GO:0006260	DNA replication	86	136	0
C.4	GO:0006281	DNA repair	133	211	0
C.4	GO:0032543	mitochondrial translation	28	36	8.85371591554955e-10
C.4	GO:0006338	chromatin remodeling	55	117	1.63138075568929e-06
C.4	GO:0002181	cytoplasmic translation	42	54	1.39399602971935e-14
C.4	GO:0042273	ribosomal large subunit biogenesis	22	30	6.60231131129136e-07
C.4	GO:0000184	nuclear-transcribed mRNA catabolic process, nonsense-mediated decay	101	117	0
C.4	GO:0007062	sister chromatid cohesion	55	108	5.26451210598989e-08
C.4	GO:0071897	DNA biosynthetic process	16	25	0.000516310721454489
C.4	GO:0006369	termination of RNA polymerase II transcription	63	69	0
C.4	GO:0031124	mRNA 3'-end processing	49	55	0
C.4	GO:0045815	positive regulation of gene expression, epigenetic	27	36	8.01578432255381e-09
C.4	GO:0006626	protein targeting to mitochondrion	18	36	0.00911399870494888
C.4	GO:0043248	proteasome assembly	9	11	0.00158721536168761
C.4	GO:0009411	response to UV	21	41	0.0025111560591991
C.4	GO:0006370	7-methylguanosine mRNA capping	25	33	2.46131177063556e-08
C.4	GO:0050434	positive regulation of viral transcription	18	27	8.09005760938227e-05
C.4	GO:0051123	RNA polymerase II transcriptional preinitiation complex assembly	13	21	0.00386329773736957
C.4	GO:1901796	regulation of signal transduction by p53 class mediator	81	135	0
C.4	GO:0075522	IRES-dependent viral translational initiation	9	10	0.000441040283702821
C.4	GO:0000082	G1/S transition of mitotic cell cycle	52	104	3.13765486259499e-07
C.4	GO:0070317	negative regulation of G0 to G1 transition	19	38	0.00665841517553432
C.4	GO:0006610	ribosomal protein import into nucleus	7	8	0.00456576284498184
C.4	GO:0006414	translational elongation	13	20	0.00203514066985584
C.4	GO:0048025	negative regulation of mRNA splicing, via spliceosome	15	20	6.07264961973356e-05
C.4	GO:0070584	mitochondrion morphogenesis	12	20	0.00911399870494888
C.4	GO:0006396	RNA processing	54	77	0
C.4	GO:0007099	centriole replication	11	17	0.0064029125581949
C.4	GO:0042795	snRNA transcription by RNA polymerase II	48	70	3.71732274591826e-13
C.4	GO:0000724	double-strand break repair via homologous recombination	50	80	1.9132595507898e-11
C.4	GO:0006303	double-strand break repair via nonhomologous end joining	35	54	1.10157163677532e-08
C.4	GO:0000055	ribosomal large subunit export from nucleus	8	9	0.00144145727420984
C.4	GO:0000463	maturation of LSU-rRNA from tricistronic rRNA transcript (SSU-rRNA, 5.8S rRNA, LSU-rRNA)	9	9	6.96640685900127e-05

C.4	GO:0006364	rRNA processing	172	205	0
C.4	GO:0000375	RNA splicing, via transesterification reactions	22	25	1.30554064528806e-09
C.4	GO:0030490	maturation of SSU-rRNA	14	18	6.2629112274567e-05
C.4	GO:0007059	chromosome segregation	32	67	0.000405222830494786
C.4	GO:0045727	positive regulation of translation	30	62	0.000516310721454489
C.4	GO:0048208	COPII vesicle coating	30	63	0.000731300494805568
C.4	GO:0045899	positive regulation of RNA polymerase II transcriptional preinitiation complex assembly	10	11	0.000132203242903511
C.4	GO:0030521	androgen receptor signaling pathway	25	46	0.000206614330427486
C.4	GO:0019827	stem cell population maintenance	23	47	0.00298095604413514
C.4	GO:0043044	ATP-dependent chromatin remodeling	15	24	0.00126355594541138
C.4	GO:0010498	proteasomal protein catabolic process	17	33	0.0083367977766903
C.4	GO:0043923	positive regulation by host of viral transcription	11	17	0.0064029125581949
C.4	GO:0030488	tRNA methylation	19	27	1.52657860613393e-05
C.4	GO:0032212	positive regulation of telomere maintenance via telomerase	22	34	1.76535209290723e-05
C.4	GO:0006353	DNA-templated transcription, termination	8	9	0.00144145727420984
C.4	GO:0007004	telomere maintenance via telomerase	13	20	0.00203514066985584
C.4	GO:0032481	positive regulation of type I interferon production	27	50	0.000114643762708916
C.4	GO:0000245	spliceosomal complex assembly	25	29	1.64743637171832e-10
C.4	GO:0051170	import into nucleus	18	21	1.83644588885074e-07
C.4	GO:0019985	translesion synthesis	21	36	0.00024499704420954
C.4	GO:0006189	'de novo' IMP biosynthetic process	6	6	0.00316266256790981
C.4	GO:0009168	purine ribonucleoside monophosphate biosynthetic process	10	13	0.00157519962246477
C.4	GO:0001682	tRNA 5'-leader removal	11	12	4.2046453882127e-05
C.4	GO:0043928	exonucleolytic nuclear-transcribed mRNA catabolic process involved in deadenylation-dependent decay	27	33	2.48209527761099e-10
C.4	GO:0071044	histone mRNA catabolic process	12	14	5.71550149708888e-05
C.4	GO:0006361	transcription initiation from RNA polymerase I promoter	27	32	6.8347293433323e-11
C.4	GO:0006362	transcription elongation from RNA polymerase I promoter	26	29	9.69377502245625e-12
C.4	GO:0006363	termination of RNA polymerase I transcription	27	31	1.49318420875707e-11
C.4	GO:0070125	mitochondrial translational elongation	70	88	0
C.4	GO:0070126	mitochondrial translational termination	72	90	0
C.4	GO:0034644	cellular response to UV	26	51	0.000558423446403068
C.4	GO:0000732	strand displacement	20	26	7.58892072213771e-07
C.4	GO:0010212	response to ionizing radiation	26	50	0.000374292606755673
C.4	GO:0006446	regulation of translational initiation	18	27	8.09005760938227e-05
C.4	GO:0006283	transcription-coupled nucleotide-excision repair	54	73	0
C.4	GO:0016070	RNA metabolic process	37	46	1.16166335809946e-13
C.4	GO:0071479	cellular response to ionizing radiation	18	31	0.000987185501811267

C.4	GO:0033683	nucleotide-excision repair, DNA incision	26	38	3.54496039572488e-07
C.4	GO:0006418	tRNA aminoacylation for protein translation	29	38	8.85371591554955e-10
C.4	GO:0000244	spliceosomal tri-snRNP complex assembly	11	12	4.2046453882127e-05
C.4	GO:0000722	telomere maintenance via recombination	8	10	0.00472860687921588
C.4	GO:0032790	ribosome disassembly	7	8	0.00456576284498184
C.4	GO:2000234	positive regulation of rRNA processing	8	9	0.00144145727420984
C.4	GO:0042769	DNA damage response, detection of DNA damage	26	39	7.7825798415156e-07
C.4	GO:0000380	alternative mRNA splicing, via spliceosome	12	16	0.000514622595383152
C.4	GO:1901409	positive regulation of phosphorylation of RNA polymerase II C-terminal domain	8	10	0.00472860687921588
C.4	GO:0042276	error-prone translesion synthesis	13	21	0.00386329773736957
C.4	GO:1904851	positive regulation of establishment of protein localization to telomere	10	10	2.03195823067793e-05
C.4	GO:1904871	positive regulation of protein localization to Cajal body	9	9	6.96640685900127e-05
C.4	GO:1904874	positive regulation of telomerase RNA localization to Cajal body	15	15	2.38671396639301e-08
C.4	GO:0006289	nucleotide-excision repair	23	38	4.83945499166023e-05
C.4	GO:0000729	DNA double-strand break processing	12	15	0.000182490241672998
C.4	GO:0000447	endonucleolytic cleavage in ITS1 to separate SSU-rRNA from 5.8S rRNA and LSU-rRNA from tricistronic rRNA transcript (SSU-rRNA, 5.8S rRNA, LSU-rRNA)	9	9	6.96640685900127e-05
C.4	GO:0000480	endonucleolytic cleavage in 5'-ETS of tricistronic rRNA transcript (SSU-rRNA, 5.8S rRNA, LSU-rRNA)	6	6	0.00316266256790981
C.4	GO:0008033	tRNA processing	18	23	2.3210723632446e-06
C.4	GO:0042407	cristae formation	17	31	0.00349731608070675
C.4	GO:0000470	maturation of LSU-rRNA	14	16	5.25869381535659e-06
C.4	GO:0006614	SRP-dependent cotranslational protein targeting to membrane	80	89	0
C.4	GO:0006379	mRNA cleavage	9	11	0.00158721536168761
C.4	GO:0000389	mRNA 3'-splice site recognition	6	6	0.00316266256790981
C.4	GO:0043968	histone H2A acetylation	11	16	0.00316266256790981
C.4	GO:0006388	tRNA splicing, via endonucleolytic cleavage and ligation	10	15	0.00827507259825226
C.4	GO:0008334	histone mRNA metabolic process	11	12	4.2046453882127e-05
C.4	GO:0006297	nucleotide-excision repair, DNA gap filling	17	24	4.78145723813449e-05
C.4	GO:0032211	negative regulation of telomere maintenance via telomerase	14	20	0.000385322031857243
C.4	GO:0006293	nucleotide-excision repair, preincision complex stabilization	15	21	0.000141951467933417
C.4	GO:0006295	nucleotide-excision repair, DNA incision, 3'-to lesion	15	22	0.000319395377650084

C.4	GO:0006296	nucleotide-excision repair, DNA incision, 5'-to lesion	26	37	1.55667248748275e-07
C.4	GO:0070911	global genome nucleotide-excision repair	21	32	2.19644859354259e-05
C.4	GO:0000715	nucleotide-excision repair, DNA damage recognition	15	23	0.000648816353323766
C.4	GO:0000717	nucleotide-excision repair, DNA duplex unwinding	15	22	0.000319395377650084
C.4	GO:0006294	nucleotide-excision repair, preincision complex assembly	22	29	2.43966308025699e-07
C.4	GO:0006271	DNA strand elongation involved in DNA replication	7	8	0.00456576284498184
C.4	GO:0032201	telomere maintenance via semi-conservative replication	22	24	2.32579525083487e-10
C.4	GO:0070987	error-free translesion synthesis	15	19	2.19644859354259e-05
C.4	GO:0009113	purine nucleobase biosynthetic process	7	7	0.000917588816104897
C.4	GO:0006999	nuclear pore organization	7	8	0.00456576284498184
C.4	GO:0010793	regulation of mRNA export from nucleus	7	8	0.00456576284498184
C.4	GO:0001188	RNA polymerase I transcriptional preinitiation complex assembly	8	9	0.00144145727420984
C.4	GO:1901838	positive regulation of transcription of nucleolar large rRNA by RNA polymerase I	8	10	0.00472860687921588
C.4	GO:0045910	negative regulation of DNA recombination	6	6	0.00316266256790981
C.4	GO:0051096	positive regulation of helicase activity	6	6	0.00316266256790981
C.4	GO:1900264	positive regulation of DNA-directed DNA polymerase activity	6	6	0.00316266256790981
C.4	GO:0070131	positive regulation of mitochondrial translation	9	11	0.00158721536168761
C.4	GO:0051415	microtubule nucleation by interphase microtubule organizing center	6	6	0.00316266256790981
C.4	GO:0046784	viral mRNA export from host cell nucleus	8	8	0.000248545204909958
C.4	GO:0080009	mRNA methylation	8	10	0.00472860687921588
C.4	GO:0001510	RNA methylation	14	18	6.2629112274567e-05

Table S3 Pathways (GO terms and Reactome pathways) associated with HLA status in PCNSL

gene clusters	GO term	GO description	intersect genes number	n.pw	FDR
C.1	GO:0006955	immune response	11	450	6.62301868015191e-08
C.1	GO:0006958	complement activation, classical pathway	3	152	0.015667939307991
C.1	GO:0030449	regulation of complement activation	3	109	0.00731353810387936
C.1	GO:0050776	regulation of immune response	3	208	0.0302318652956718
C.1	GO:0006954	inflammatory response	7	392	0.000243882953531283
C.1	GO:0043066	negative regulation of apoptotic process	5	525	0.0192498016008339
C.1	GO:0007165	signal transduction	8	1211	0.0184570876868354
C.1	GO:0007166	cell surface receptor signaling pathway	3	259	0.0444091144794976
C.1	GO:0050852	T cell receptor signaling pathway	11	174	3.10285130922239e-12
C.1	GO:0042102	positive regulation of T cell proliferation	4	60	9.43445160533589e-05
C.1	GO:0043312	neutrophil degranulation	6	481	0.00382621296618498
C.1	GO:0019886	antigen processing and presentation of exogenous peptide antigen via MHC class II	10	98	1.31927802016207e-12
C.1	GO:0002576	platelet degranulation	3	124	0.00991676186081192
C.1	GO:0031295	T cell costimulation	9	75	2.14028794687238e-12
C.1	GO:0060333	interferon-gamma-mediated signaling pathway	9	71	1.91946458727443e-12
C.2	GO:0007186	G-protein coupled receptor signaling pathway	8	1143	0.0008901120427213
C.2	GO:0006955	immune response	4	450	0.00760075096013457
C.2	GO:0007165	signal transduction	5	1211	0.0457187269545129
C.2	GO:0043312	neutrophil degranulation	6	481	0.00043711912458352
C.2	GO:0007204	positive regulation of cytosolic calcium ion concentration	3	137	0.00284006080527319
C.2	GO:0006874	cellular calcium ion homeostasis	3	88	0.000963240549427706
C.2	GO:0008015	blood circulation	3	43	0.00043711912458352
C.3	GO:0006955	immune response	6	450	0.000442989503746105
C.3	GO:0002250	adaptive immune response	8	255	1.91991843534467e-07
C.3	GO:0050776	regulation of immune response	7	208	7.88796393017913e-07
C.3	GO:0045087	innate immune response	6	516	0.000572192469919858
C.3	GO:0007169	transmembrane receptor protein tyrosine kinase signaling pathway	5	105	8.96176950371252e-06
C.3	GO:0006954	inflammatory response	6	392	0.000251877719122323
C.3	GO:0070098	chemokine-mediated signaling pathway	3	71	0.000657878210874538
C.3	GO:0007166	cell surface receptor signaling pathway	7	259	2.64413860667778e-06
C.3	GO:0050852	T cell receptor signaling pathway	5	174	7.66923047280337e-05
C.3	GO:0007267	cell-cell signaling	3	317	0.0247539323363789
C.3	GO:0042102	positive regulation of T cell proliferation	3	60	0.000546497165013167
C.3	GO:0006915	apoptotic process	4	541	0.0204634557753719
C.3	GO:0009615	response to virus	3	106	0.00168960868673164
C.3	GO:0019221	cytokine-mediated signaling pathway	3	311	0.0243568954152458
C.3	GO:0006952	defense response	3	65	0.000572192469919858
C.3	GO:0051260	protein homooligomerization	4	203	0.000927578410489436

C.3	GO:0006935	chemotaxis	3	123	0.00249586046231036
C.3	GO:0042110	T cell activation	6	42	3.68079677848243e-09
C.3	GO:0031295	T cell costimulation	4	75	6.67039406180662e-05
C.3	GO:0009967	positive regulation of signal transduction	3	59	0.000546497165013167
C.1	R-HSA-109582	Hemostasis	7	699	0.0299132234633671
C.1	R-HSA-1280218	Adaptive Immune System	15	846	1.50088287931283e-06
C.1	R-HSA-166658	Complement cascade	3	144	0.0299132234633671
C.1	R-HSA-168249	Innate Immune System	11	1129	0.0103204700428954
C.1	R-HSA-168256	Immune System	24	2065	4.06380963304607e-07
C.1	R-HSA-977606	Regulation of Complement cascade	3	134	0.0271848178943493
C.1	R-HSA-1280215	Cytokine Signaling in Immune system	11	672	0.000137112031331445
C.1	R-HSA-202403	TCR signaling	9	124	5.05904207415142e-09
C.1	R-HSA-202424	Downstream TCR signaling	8	103	2.40003284406814e-08
C.1	R-HSA-2132295	MHC class II antigen presentation	10	126	2.95873547884185e-10
C.1	R-HSA-913531	Interferon Signaling	9	194	2.06496972410264e-07
C.1	R-HSA-76002	Platelet activation, signaling and aggregation	4	260	0.0299132234633671
C.1	R-HSA-6798695	Neutrophil degranulation	6	479	0.0246134242633866
C.1	R-HSA-76005	Response to elevated platelet cytosolic Ca2+	3	133	0.0271848178943493
C.1	R-HSA-114608	Platelet degranulation	3	128	0.0267440064513832
C.1	R-HSA-388841	Costimulation by the CD28 family	9	72	5.65023583476432e-11
C.1	R-HSA-877300	Interferon gamma signaling	9	92	3.88632871915563e-10
C.1	R-HSA-202433	Generation of second messenger molecules	9	39	6.18172180111287e-13
C.1	R-HSA-202427	Phosphorylation of CD3 and TCR zeta chains	8	28	1.17476398979003e-12
C.1	R-HSA-202430	Translocation of ZAP-70 to Immunological synapse	8	25	6.18172180111287e-13
C.1	R-HSA-389948	PD-1 signaling	8	29	1.21325172131037e-12
C.2	R-HSA-418594	G alpha (i) signalling events	4	393	0.0487655169836094
C.2	R-HSA-6798695	Neutrophil degranulation	6	479	0.0121586239422841
C.2	R-HSA-500792	GPCR ligand binding	5	455	0.0306741237637812
C.2	R-HSA-373076	Class A/1 (Rhodopsin-like receptors)	5	322	0.0121586239422841
C.2	R-HSA-375276	Peptide ligand-binding receptors	3	187	0.0399740405628638
C.3	R-HSA-1280218	Adaptive Immune System	8	846	0.0056369226966999
C.3	R-HSA-168256	Immune System	15	2065	0.000711086456718389
C.3	R-HSA-198933	Immunoregulatory interactions between a Lymphoid and a non-Lymphoid cell	7	221	5.86657789623524e-05
C.3	R-HSA-202403	TCR signaling	4	124	0.00125773484956825
C.3	R-HSA-202424	Downstream TCR signaling	3	103	0.00728127334246723
C.3	R-HSA-375276	Peptide ligand-binding receptors	3	187	0.0322584633104336
C.3	R-HSA-380108	Chemokine receptors bind chemokines	3	48	0.00118649614887181
C.3	R-HSA-388841	Costimulation by the CD28 family	4	72	0.000343802530555937
C.3	R-HSA-202433	Generation of second messenger molecules	4	39	5.86657789623524e-05
C.3	R-HSA-202427	Phosphorylation of CD3 and TCR zeta chains	3	28	0.000346596837453528
C.3	R-HSA-202430	Translocation of ZAP-70 to Immunological synapse	3	25	0.000343802530555937
C.3	R-HSA-389948	PD-1 signaling	3	29	0.000346596837453528

Table S4 Pathways (GO terms and Reactome pathways) associated with HLA class I gene expression in PCNSL

gene clusters	GO term	GO description	intersect genes number	n.pw	FDR
C.1	GO:0006955	immune response	4	450	0.0139212198384278
C.1	GO:0002250	adaptive immune response	7	255	8.3890723225899e-06
C.1	GO:0050776	regulation of immune response	5	208	0.00017290202925128
C.1	GO:0045087	innate immune response	5	516	0.00574135763550225
C.1	GO:0006954	inflammatory response	4	392	0.0102622648163284
C.1	GO:0043066	negative regulation of apoptotic process	4	525	0.0205572960729634
C.1	GO:0007165	signal transduction	7	1211	0.0102622648163284
C.1	GO:0045944	positive regulation of transcription by RNA polymerase II	5	1063	0.0440869942156194
C.1	GO:0007166	cell surface receptor signaling pathway	6	259	4.56636151145995e-05
C.1	GO:0050852	T cell receptor signaling pathway	5	174	8.77527762527919e-05
C.1	GO:0007267	cell-cell signaling	3	317	0.0253026103648645
C.1	GO:0042110	T cell activation	4	42	1.51665531673562e-05
C.1	GO:0031295	T cell costimulation	3	75	0.00115043379961304
C.1	GO:0006959	humoral immune response	3	57	0.000708853713821472
C.2	GO:0006955	immune response	4	450	0.00448646741409986
C.2	GO:0006508	proteolysis	3	522	0.029873579479573
C.2	GO:0006956	complement activation	3	91	0.00127939290571533
C.2	GO:0030449	regulation of complement activation	3	109	0.00145397078881082
C.2	GO:0045087	innate immune response	3	516	0.029873579479573
C.2	GO:0006954	inflammatory response	4	392	0.00370657711421174
C.2	GO:0043312	neutrophil degranulation	6	481	0.000229488095136787
C.1	R-HSA-168256	Immune System	13	2065	0.0192256333390624
C.1	R-HSA-198933	Immunoregulatory interactions between a Lymphoid and a non-Lymphoid cell	5	221	0.0137332478625909
C.1	R-HSA-388841	Costimulation by the CD28 family	3	72	0.0192256333390624
C.2	R-HSA-166658	Complement cascade	4	144	0.00140293434230215
C.2	R-HSA-166663	Initial triggering of complement	3	109	0.00421239671141556
C.2	R-HSA-168249	Innate Immune System	10	1129	0.000368496968165544
C.2	R-HSA-168256	Immune System	11	2065	0.00330404481217522
C.2	R-HSA-977606	Regulation of Complement cascade	3	134	0.00635017888650365
C.2	R-HSA-6798695	Neutrophil degranulation	6	479	0.00144086520055024

Table S5 GO terms associated with high HLA-DRA expression in PCNSL

gene clusters	GO term	GO description	intersect genes number	n.pw	FDR
C.1	GO:0006955	immune response	13	450	6.23428046075958e-08
C.1	GO:0006958	complement activation, classical pathway	3	152	0.0382372309515108
C.1	GO:0050776	regulation of immune response	4	208	0.0216942292899246
C.1	GO:0045087	innate immune response	7	516	0.0109417967542629
C.1	GO:0006954	inflammatory response	6	392	0.0115621659324993
C.1	GO:0043066	negative regulation of apoptotic process	6	525	0.0290649654304541
C.1	GO:0007155	cell adhesion	8	465	0.00178016260760732
C.1	GO:0071222	cellular response to lipopolysaccharide	3	131	0.0303696241815757
C.1	GO:0044267	cellular protein metabolic process	4	192	0.0184944502795765
C.1	GO:0043065	positive regulation of apoptotic process	4	315	0.0477022335970671
C.1	GO:0043687	post-translational protein modification	5	355	0.0253037711750362
C.1	GO:0050852	T cell receptor signaling pathway	10	174	8.93487100972479e-09
C.1	GO:0042102	positive regulation of T cell proliferation	4	60	0.000660792323790543
C.1	GO:0007160	cell-matrix adhesion	3	93	0.0184944502795765
C.1	GO:0019221	cytokine-mediated signaling pathway	7	311	0.00100170405430619
C.1	GO:0006952	defense response	3	65	0.00963043950474303
C.1	GO:0043312	neutrophil degranulation	5	481	0.0487897195971517
C.1	GO:0019886	antigen processing and presentation of exogenous peptide antigen via MHC class II	11	98	2.60147459130167e-12
C.1	GO:0042127	regulation of cell proliferation	4	197	0.0186358479450804
C.1	GO:0051092	positive regulation of NF-kappaB transcription factor activity	3	142	0.0353379424647638
C.1	GO:0030198	extracellular matrix organization	4	197	0.0186358479450804
C.1	GO:0031295	T cell costimulation	9	75	1.22815017415216e-10
C.1	GO:0060333	interferon-gamma-mediated signaling pathway	9	71	1.1039447134209e-10
C.1	GO:0007568	aging	4	157	0.0114631252456456
C.1	GO:0009612	response to mechanical stimulus	3	60	0.00818224537933379
C.1	GO:0048146	positive regulation of fibroblast proliferation	3	50	0.00611118521392581
C.1	GO:0043410	positive regulation of MAPK cascade	3	143	0.0353379424647638
C.1	R-HSA-1280218	Adaptive Immune System	17	846	1.59689284169488e-05

C.1	R-HSA-168256	Immune System	28	2065	1.25381304037682e-05
C.1	R-HSA-1280215	Cytokine Signaling in Immune system	17	672	7.465236082234e-07
C.1	R-HSA-202403	TCR signaling	9	124	2.36209612369809e-07
C.1	R-HSA-202424	Downstream TCR signaling	8	103	7.465236082234e-07
C.1	R-HSA-449147	Signaling by Interleukins	7	451	0.0340228514572806
C.1	R-HSA-1474244	Extracellular matrix organization	8	300	0.00126434045649886
C.1	R-HSA-2132295	MHC class II antigen presentation	11	126	1.08293248442711e-09
C.1	R-HSA-913531	Interferon Signaling	10	194	7.465236082234e-07
C.1	R-HSA-381426	Regulation of Insulin-like Growth Factor (IGF) transport and uptake by Insulin-like Growth Factor Binding Proteins (IGFBPs)	4	124	0.015528303046484
C.1	R-HSA-8957275	Post-translational protein phosphorylation	4	107	0.00964438829075351
C.1	R-HSA-388841	Costimulation by the CD28 family	9	72	2.04155594017509e-09
C.1	R-HSA-6783783	Interleukin-10 signaling	3	47	0.00755489869267778
C.1	R-HSA-6785807	Interleukin-4 and 13 signaling	5	111	0.0016801458967102
C.1	R-HSA-877300	Interferon gamma signaling	10	92	1.08293248442711e-09
C.1	R-HSA-202433	Generation of second messenger molecules	9	39	2.21962448421209e-11
C.1	R-HSA-1566948	Elastic fibre formation	3	45	0.00703600662572493
C.1	R-HSA-2129379	Molecules associated with elastic fibres	3	38	0.00484254557263875
C.1	R-HSA-202427	Phosphorylation of CD3 and TCR zeta chains	8	28	4.19541438626917e-11
C.1	R-HSA-202430	Translocation of ZAP-70 to Immunological synapse	8	25	2.21962448421209e-11
C.1	R-HSA-389948	PD-1 signaling	8	29	4.32575641973187e-11

article title	**The immune landscapes of primary central nervous diffuse large B cell lymphoma**

author names	Melissa Alame[1,2,3,4], Emmanuel Cornillot[1,3,4,7], Valère Cacheux[2,5,7], Valérie Rigau[5,6], Valérie Costes-Martineau[5,6], Vanessa Lacheretz-Szablewski[5,6,8], Jacques Colinge[1,4,5,8]
affiliation	1 Institut de Recherche en Cancérologie de Montpellier (IRCM), INSERM, Parc Euromédecine, 208 rue des Apothicaires, 34298 Montpellier, France<br>2 Biological Hematology Department, CHU Montpellier, Hôpital Saint Eloi, 34275 Montpellier, France<br>3 Université de Montpellier, Faculté de Pharmacie, 15 avenue Charles Flahault, 34093 Montpellier, France<br>4 Institut régional du Cancer Montpellier (ICM), Parc Euromédecine, 208 rue des Apothicaires, 34298 Montpellier, France<br>5 Université de Montpellier, Faculté de Médecine, 2 rue école de Médecine, 34060 Montpellier, France<br>6 Département de pathologie et d'oncologie biologique, CHU Montpellier, Hôpital Gui De Chauliac, 34000 Montpellier, France<br>7 Equal contribution<br>8 Corresponding authors: jacques.colinge@inserm.fr, v-szablewski@chu-montpellier.fr

Description of column titles for Supplementary Tables S6-S7

**Ligand**	Ligand gene expression
**Receptor**	Receptor gene expression
**therapeutical target (Ligand)**	potential target used in clinical trial* (examples are given in brackets)
**therapeutical target (Receptor)**	potential target used in clinical trial* (examples are given in brackets)
**corr.glob**	Spearman correlation between ligand and receptor gene expression
**num.cor.pw**	number of target genes that are correlated (r>0.5) with the receptor of the LR pair in each pathway
**pwid**	Reactome and Gene Ontology terms ID
**pwname**	Reactome and Gene Ontology term names
**L.ct**	Cell types expressing the ligand transcript of the LR pair
**R.ct**	Cell types expressing the receptor transcript of the LR pair

Table S6 L-R pairs selected by RNA-sequencing analysis (n=20)

Ligand	Receptor	corr.glob	num.cor.pw	pwid	pwname	L.ct	R.ct
A2M	LRP1	0.786466165413534	4	R-HSA-196854|R-HSA-2187338|R-HSA-68066	Metabolism of vitamins and cofactors|Visual phototransduction|Meta	Monocytic lineage;Neutrophils;Endothelial ce	Monocytic lineage;Endothelial cells
ADAM10	AXL	0.705263157894737	4	R-HSA-194138|R-HSA-4420097	Signaling by VEGF|VEGFA-VEGFR2 Pathway	Endothelial cells	T cells;Monocytic lineage;Endothelial cells
ADAM15	ITGA9	0.64812030075188	4	R-HSA-373760	L1CAM interactions	Endothelial cells	Monocytic lineage;Endothelial cells
ADAM15	ITGAV	0.697744360902256	4	R-HSA-194138|R-HSA-202733|R-HSA-373760	Signaling by VEGF|Cell surface interactions at the vascular wall|L1C	Endothelial cells	Monocytic lineage;Neutrophils;Endothelial cells
ADAM17	ITGB1	0.609022556390977	4	R-HSA-6785807	Interleukin-4 and 13 signaling	NA	Endothelial cells
ADAM9	ITGA6	0.765413533834587	4	R-HSA-1474290|R-HSA-1500931|R-HSA-2022	Collagen formation|Cell-Cell communication|Assembly of collagen fi	Monocytic lineage;Endothelial cells	Neutrophils;Endothelial cells
ADAM9	ITGAV	0.926315789473684	4	R-HSA-194138|R-HSA-202733|R-HSA-373760	Signaling by VEGF|Cell surface interactions at the vascular wall|L1C	Monocytic lineage;Endothelial cells	Monocytic lineage;Neutrophils;Endothelial cells
ADAM9	ITGB5	0.897744360902256	4	R-HSA-445355	Smooth Muscle Contraction	Monocytic lineage;Endothelial cells	Monocytic lineage;Endothelial cells
AGRN	LRP1	0.747368421052632	4	R-HSA-196854|R-HSA-2187338|R-HSA-68066	Metabolism of vitamins and cofactors|Visual phototransduction|Meta	Neutrophils;Endothelial cells	Monocytic lineage;Endothelial cells
AGRN	LRP2	0.601503759398496	4	R-HSA-196854|R-HSA-2187338|R-HSA-68066	Metabolism of vitamins and cofactors|Visual phototransduction|Meta	Neutrophils;Endothelial cells	Neutrophils;Endothelial cells
AGT	LRP2	0.759398496240602	4	R-HSA-196854|R-HSA-2187338|R-HSA-68066	Metabolism of vitamins and cofactors|Visual phototransduction|Meta	Neutrophils;Endothelial cells	Neutrophils;Endothelial cells
AHSG	INSR	0.77636918331219	4	R-HSA-199418|R-HSA-6811558|R-HSA-74751	Negative regulation of the PI3K/AKT network|PI5P, PP2A and IER3	Endothelial cells	Neutrophils;Endothelial cells
ALB	LRP2	0.739458669912053	4	R-HSA-196854|R-HSA-2187338|R-HSA-68066	Metabolism of vitamins and cofactors|Visual phototransduction|Meta	NA	Neutrophils;Endothelial cells
ANGPT1	TEK	0.753383458646616	4	R-HSA-202733	Cell surface interactions at the vascular wall	Neutrophils;Endothelial cells	T cells;Monocytic lineage;Neutrophils;Endothelial cells
ANGPTL1	TEK	0.784962406015038	4	R-HSA-202733	Cell surface interactions at the vascular wall	Neutrophils;Endothelial cells	T cells;Monocytic lineage;Neutrophils;Endothelial cells
ANOS1	SDC2	0.742857142857143	4	R-HSA-1630316|R-HSA-1638091|R-HSA-1793	Glycosaminoglycan metabolism|Heparan sulfate/heparin (HS-GAG) r	Monocytic lineage;Endothelial cells	Neutrophils;Endothelial cells
ANXA1	DYSF	0.581954887218045	4	R-HSA-445355	Smooth Muscle Contraction	T cells;Endothelial cells	Monocytic lineage;Neutrophils
APOB	OLR1	0.599473528666933	4	R-HSA-202733	Cell surface interactions at the vascular wall	Neutrophils;Endothelial cells	Endothelial cells
APOC2	LRP1	0.55187969924812	4	R-HSA-196854|R-HSA-2187338|R-HSA-68066	Metabolism of vitamins and cofactors|Visual phototransduction|Meta	NA	Monocytic lineage;Endothelial cells
APOE	LRP1	0.682706766917293	4	R-HSA-196854|R-HSA-2187338|R-HSA-68066	Metabolism of vitamins and cofactors|Visual phototransduction|Meta	T cells;Monocytic lineage;Myeloid dendritic c	Monocytic lineage;Endothelial cells
APP	GPC1	0.827067669172932	4	R-HSA-1630316|R-HSA-1638091|R-HSA-1793	Glycosaminoglycan metabolism|Heparan sulfate/heparin (HS-GAG) r	Neutrophils;Endothelial cells	Neutrophils;Endothelial cells
APP	LRP1	0.772932330827068	4	R-HSA-196854|R-HSA-2187338|R-HSA-68066	Metabolism of vitamins and cofactors|Visual phototransduction|Meta	Neutrophils;Endothelial cells	Monocytic lineage;Endothelial cells
APP	TNFRSF21	0.742857142857143	4	R-HSA-1989781|R-HSA-400206	PPARA activates gene expression|Regulation of lipid metabolism by	Neutrophils;Endothelial cells	Neutrophils;Endothelial cells
AZGP1	ITGAV	0.598721366146649	4	R-HSA-194138|R-HSA-202733|R-HSA-373760	Signaling by VEGF|Cell surface interactions at the vascular wall|L1C	Endothelial cells	Monocytic lineage;Neutrophils;Endothelial cells
B2M	CD3D	0.866165413533835	4	R-HSA-202403|R-HSA-202424|R-HSA-202427	TCR signaling|Downstream TCR signaling|Phosphorylation of CD3 a	T cells;Cytotoxic lymphocytes;NK cells;Mon	T cells;Cytotoxic lymphocytes;NK cells;Monocytic lineage;Neutrophils
B2M	CD3G	0.917293233082707	4	R-HSA-198933|R-HSA-202403|R-HSA-202424	Immunoregulatory interactions between a Lymphoid and a non-Lymp	T cells;Cytotoxic lymphocytes;NK cells;Mon	T cells;Cytotoxic lymphocytes;NK cells;Monocytic lineage
B2M	HLA-F	0.941353383458647	4	R-HSA-1236977|R-HSA-877300|R-HSA-98317	Endosomal/Vacuolar pathway|Interferon gamma signaling|Antigen Pr	T cells;Cytotoxic lymphocytes;NK cells;Mon	T cells;Cytotoxic lymphocytes;NK cells;Monocytic lineage;Myeloid dendritic c
B2M	LILRB2	0.887218045112782	4	R-HSA-198933	Immunoregulatory interactions between a Lymphoid and a non-Lymp	T cells;Cytotoxic lymphocytes;NK cells;Mon	T cells;Cytotoxic lymphocytes;NK cells;Monocytic lineage
BCAN	EGFR	0.541353383458647	4	R-HSA-1227986|R-HSA-1236394|R-HSA-1250	Signaling by ERBB2|Signaling by ERBB4|SHC1 events in ERBB2 si	Neutrophils;Endothelial cells	Neutrophils;Endothelial cells
BCAN	NRCAM	0.699248120300752	4	R-HSA-373760	L1CAM interactions	Neutrophils;Endothelial cells	Endothelial cells
BMP8A	BMPR1B	0.545864661654135	4	R-HSA-9006936	Signaling by TGF-beta family members	Neutrophils;Endothelial cells	Neutrophils;Endothelial cells
BMP8B	BMPR1B	0.607518796992481	4	R-HSA-9006936	Signaling by TGF-beta family members	Neutrophils;Endothelial cells	Neutrophils;Endothelial cells
BTLA	CD79A	0.747368421052632	4	R-HSA-983695|R-HSA-983705	Antigen activates B Cell Receptor (BCR) leading to generation of sec	B lineage	B lineage
C1QA	CR1	0.568421052631579	4	R-HSA-166658|R-HSA-977606	Complement cascade|Regulation of Complement cascade	T cells;Monocytic lineage	Monocytic lineage;Neutrophils
C1QB	LRP1	0.643609022556391	4	R-HSA-196854|R-HSA-2187338|R-HSA-68066	Metabolism of vitamins and cofactors|Visual phototransduction|Meta	T cells;Monocytic lineage	Monocytic lineage;Endothelial cells
C3	C3AR1	0.732330827067669	4	R-HSA-166658|R-HSA-977606	Complement cascade|Regulation of Complement cascade	T cells;Monocytic lineage;Neutrophils;Endotl	T cells;Monocytic lineage;Neutrophils;Endothelial cells
C3	C5AR2	0.678195488721804	4	R-HSA-166658|R-HSA-977606	Complement cascade|Regulation of Complement cascade	T cells;Monocytic lineage;Neutrophils;Endotl	Monocytic lineage;Endothelial cells
C3	CD81	0.748872180451128	4	R-HSA-166658|R-HSA-977606	Complement cascade|Regulation of Complement cascade	T cells;Monocytic lineage;Neutrophils;Endotl	Monocytic lineage;Endothelial cells
C3	LRP1	0.819548872180451	4	R-HSA-196854|R-HSA-2187338|R-HSA-68066	Metabolism of vitamins and cofactors|Visual phototransduction|Meta	T cells;Monocytic lineage;Neutrophils;Endotl	Monocytic lineage;Endothelial cells
C4A	C3AR1	0.810526315789474	4	R-HSA-166658|R-HSA-977606	Complement cascade|Regulation of Complement cascade	Monocytic lineage;Neutrophils	T cells;Monocytic lineage;Neutrophils;Endothelial cells
C4A	C5AR2	0.62406015037594	4	R-HSA-166658|R-HSA-977606	Complement cascade|Regulation of Complement cascade	Monocytic lineage;Neutrophils	Monocytic lineage;Endothelial cells
C4A	CR1	0.545864661654135	4	R-HSA-166658|R-HSA-977606	Complement cascade|Regulation of Complement cascade	Monocytic lineage;Neutrophils	Monocytic lineage;Neutrophils
C4BPA	LRP1	0.599921641650329	4	R-HSA-196854|R-HSA-2187338|R-HSA-68066	Metabolism of vitamins and cofactors|Visual phototransduction|Meta	Endothelial cells	Monocytic lineage;Endothelial cells
CALM1	ADCY8	0.760005411236147	4	R-HSA-111885|R-HSA-111933|R-HSA-111996	Opioid Signalling|Calmodulin induced events|Ca-dependent events|C	Endothelial cells	Neutrophils;Endothelial cells
CALM1	ADCYAP1R1	0.711278195488722	4	R-HSA-166520|R-HSA-187037|R-HSA-373080	Signaling by NTRKs|Signaling by NTRK1 (TRKA)|Class B/2 (Secret	Endothelial cells	Neutrophils;Endothelial cells
CALM1	GRM5	0.612952502350724	4	R-HSA-6794362	Protein-protein interactions at synapses	Endothelial cells	Endothelial cells
CALM1	KCNQ1	0.601503759398496	4	R-HSA-5576891	Cardiac conduction	Endothelial cells	Monocytic lineage;Endothelial cells
CALM1	MYLK	0.618045112781955	4	R-HSA-445355|R-HSA-5627123	Smooth Muscle Contraction|RHO GTPases activate PAKs	Endothelial cells	T cells;Neutrophils;Endothelial cells
CALM1	PDE1A	0.616541353383459	4	R-HSA-111885|R-HSA-111933|R-HSA-111996	Opioid Signalling|Calmodulin induced events|Ca-dependent events|C	Endothelial cells	Neutrophils;Endothelial cells
CALM1	PDE1C	0.592481203007519	4	R-HSA-111885|R-HSA-111933|R-HSA-111996	Opioid Signalling|Calmodulin induced events|Ca-dependent events|C	Endothelial cells	NA
CALM2	ADCY8	0.612834552059336	4	R-HSA-111885|R-HSA-111933|R-HSA-111996	Opioid Signalling|Calmodulin induced events|Ca-dependent events|C	Neutrophils	Neutrophils;Endothelial cells
CALM2	MYLK	0.639097744360902	4	R-HSA-445355|R-HSA-5627123	Smooth Muscle Contraction|RHO GTPases activate PAKs	Neutrophils	T cells;Neutrophils;Endothelial cells
CALM2	PDE1A	0.556390977443609	4	R-HSA-111885|R-HSA-111933|R-HSA-111996	Opioid Signalling|Calmodulin induced events|Ca-dependent events|C	Neutrophils	Neutrophils;Endothelial cells
CCL13	CCR2	0.518796992481203	4	R-HSA-6783783	Interleukin-10 signaling	NA	Monocytic lineage
CCL23	CCR1	0.573795831438884	4	R-HSA-6783783	Interleukin-10 signaling	T cells;Monocytic lineage	T cells;Monocytic lineage
CCL2	CCR1	0.669172932330827	4	R-HSA-6783783	Interleukin-10 signaling	T cells;Monocytic lineage	T cells;Monocytic lineage
CCL2	CCR5	0.712781954887218	4	R-HSA-162587|R-HSA-6783783	HIV Life Cycle|Interleukin-10 signaling	T cells;Monocytic lineage	T cells;Cytotoxic lymphocytes;NK cells;Monocytic lineage;Neutrophils
CCL3	CCR1	0.556390977443609	4	R-HSA-6783783	Interleukin-10 signaling	T cells	T cells;Monocytic lineage
CCL3	CCR5	0.633082706766917	4	R-HSA-162587|R-HSA-6783783	HIV Life Cycle|Interleukin-10 signaling	T cells	T cells;Cytotoxic lymphocytes;NK cells;Monocytic lineage;Neutrophils
CCL4	CCR5	0.717293233082707	4	R-HSA-162587|R-HSA-6783783	HIV Life Cycle|Interleukin-10 signaling	T cells	T cells;Cytotoxic lymphocytes;NK cells;Monocytic lineage;Neutrophils
CCL5	CCR5	0.815037593984962	4	R-HSA-162587|R-HSA-6783783	HIV Life Cycle|Interleukin-10 signaling	T cells;Cytotoxic lymphocytes;NK cells;Mon	T cells;Cytotoxic lymphocytes;NK cells;Monocytic lineage;Neutrophils
CCL7	CCR2	0.642346792323163	4	R-HSA-6783783	Interleukin-10 signaling	T cells;Monocytic lineage;Fibroblasts	Monocytic lineage
CCL7	CCR5	0.553591614929565	4	R-HSA-162587|R-HSA-6783783	HIV Life Cycle|Interleukin-10 signaling	T cells;Monocytic lineage;Fibroblasts	T cells;Cytotoxic lymphocytes;NK cells;Monocytic lineage;Neutrophils
CCL8	CCR1	0.628571428571429	4	R-HSA-6783783	Interleukin-10 signaling	T cells;Monocytic lineage	T cells;Monocytic lineage
CCL8	CCR5	0.798496240601504	4	R-HSA-162587|R-HSA-6783783	HIV Life Cycle|Interleukin-10 signaling	T cells;Monocytic lineage	T cells;Cytotoxic lymphocytes;NK cells;Monocytic lineage;Neutrophils

CD14	ITGB2	0.784962406015038	4	R-HSA-166016|R-HSA-168898|R-HSA-202733	Toll Like Receptor 4 (TLR4) Cascade|Toll-Like Receptors Cascades|(	T cells;Monocytic lineage;Endothelial cells	T cells;NK cells;Monocytic lineage;Myeloid dendritic cells;Neutrophils
CD14	RIPK1	0.548872180451128	4	R-HSA-109581|R-HSA-1606322|R-HSA-16601	Apoptosis|ZBP1(DAI) mediated induction of type I IFNs|Toll Like Re	T cells;Monocytic lineage;Endothelial cells	T cells;Cytotoxic lymphocytes;NK cells;Monocytic lineage;Myeloid dendritic c
CD40LG	ITGB2	0.562406015037594	4	R-HSA-166016|R-HSA-168898|R-HSA-202733	Toll Like Receptor 4 (TLR4) Cascade|Toll-Like Receptors Cascades|(	T cells	T cells;NK cells;Monocytic lineage;Myeloid dendritic cells;Neutrophils
CD86	CD28	0.628571428571429	4	R-HSA-2219528|R-HSA-388841|R-HSA-38935	PI3K/AKT Signaling in Cancer|Costimulation by the CD28 family|CI	T cells;Cytotoxic lymphocytes;NK cells;Mono	T cells;Cytotoxic lymphocytes;NK cells;Monocytic lineage;Neutrophils
CD86	CTLA4	0.613533834586466	4	R-HSA-388841	Costimulation by the CD28 family	T cells;Cytotoxic lymphocytes;NK cells;Mono	T cells;Cytotoxic lymphocytes;Monocytic lineage
CDH1	PTPRF	0.583458646616541	4	R-HSA-6794362|R-HSA-8849932	Protein-protein interactions at synapses|Synaptic adhesion-like molec	NA	Neutrophils;Endothelial cells
CFH	SELL	0.545864661654135	4	R-HSA-202733	Cell surface interactions at the vascular wall	T cells;Cytotoxic lymphocytes;NK cells;Mono	T cells;Cytotoxic lymphocytes;NK cells
CGN	OCLN	0.687218045112782	4	R-HSA-109581|R-HSA-111465|R-HSA-535780	Apoptosis|Apoptotic cleavage of cellular proteins|Programmed Cell D	NA	Neutrophils;Endothelial cells
CGN	TGFBR1	0.518796992481203	4	R-HSA-170834|R-HSA-5689880|R-HSA-90069	Signaling by TGF-beta Receptor Complex|Ub-specific processing pro	NA	T cells;Monocytic lineage;Neutrophils;Endothelial cells;Fibroblasts
CNTN2	CNTN1	0.769924812030075	4	R-HSA-157118|R-HSA-1980143|R-HSA-37376	Signaling by NOTCH|Signaling by NOTCH1|L1CAM interactions	Neutrophils;Endothelial cells	Neutrophils;Endothelial cells
CNTN2	NRCAM	0.57593984962406	4	R-HSA-373760	L1CAM interactions	Neutrophils;Endothelial cells	Endothelial cells
COL18A1	GPC1	0.590977443609023	4	R-HSA-1630316|R-HSA-1638091|R-HSA-1793	Glycosaminoglycan metabolism|Heparan sulfate/heparin (HS-GAG) r	Neutrophils;Endothelial cells	Neutrophils;Endothelial cells
COL18A1	GPC4	0.849624060150376	4	R-HSA-1630316|R-HSA-1638091|R-HSA-1793	Glycosaminoglycan metabolism|Heparan sulfate/heparin (HS-GAG) r	Neutrophils;Endothelial cells	Neutrophils;Endothelial cells
COL18A1	ITGB1	0.709774436090226	4	R-HSA-6785807	Interleukin-4 and 13 signaling	Neutrophils;Endothelial cells	Endothelial cells
COL18A1	ITGB3	0.536842105263158	4	R-HSA-202733|R-HSA-354192|R-HSA-567413	Cell surface interactions at the vascular wall|Integrin alphaIIb beta3 si	Neutrophils;Endothelial cells	Monocytic lineage;Endothelial cells;Fibroblasts
COL18A1	ITGB5	0.700751879699248	4	R-HSA-445355	Smooth Muscle Contraction	Neutrophils;Endothelial cells	Monocytic lineage;Endothelial cells
COL18A1	KDR	0.613533834586466	4	R-HSA-194138|R-HSA-4420097	Signaling by VEGF|VEGFA-VEGFR2 Pathway	Neutrophils;Endothelial cells	Endothelial cells
COL1A1	CD44	0.643609022556391	4	R-HSA-1474228|R-HSA-202733	Degradation of the extracellular matrix|Cell surface interactions at the	Monocytic lineage;Endothelial cells;Fibroblas	Monocytic lineage;Fibroblasts
COL1A1	ITGA5	0.902255639097744	4	R-HSA-1566977|R-HSA-202733	Fibronectin matrix formation|Cell surface interactions at the vascular	Monocytic lineage;Endothelial cells;Fibroblas	NK cells;Monocytic lineage;Endothelial cells;Fibroblasts
COL1A2	CD36	0.616541353383459	4	R-HSA-166016|R-HSA-168898|R-HSA-198978	Toll Like Receptor 4 (TLR4) Cascade|Toll-Like Receptors Cascades|I	Monocytic lineage;Endothelial cells;Fibroblas	Fibroblasts
COL1A2	CD44	0.6	4	R-HSA-1474228|R-HSA-202733	Degradation of the extracellular matrix|Cell surface interactions at the	Monocytic lineage;Endothelial cells;Fibroblas	Monocytic lineage;Fibroblasts
COL1A2	ITGA1	0.569924812030075	4	R-HSA-445355	Smooth Muscle Contraction	Monocytic lineage;Endothelial cells;Fibroblas	Endothelial cells
COL1A2	ITGB1	0.556390977443609	4	R-HSA-6785807	Interleukin-4 and 13 signaling	Monocytic lineage;Endothelial cells;Fibroblas	Endothelial cells
COL1A2	ITGB3	0.745864661654135	4	R-HSA-202733|R-HSA-354192|R-HSA-567413	Cell surface interactions at the vascular wall|Integrin alphaIIb beta3 si	Monocytic lineage;Endothelial cells;Fibroblas	Monocytic lineage;Endothelial cells;Fibroblasts
COL4A1	ITGA1	0.607518796992481	4	R-HSA-445355	Smooth Muscle Contraction	Fibroblasts	Endothelial cells
COL4A2	ITGB3	0.723308270676692	4	R-HSA-202733|R-HSA-354192|R-HSA-567413	Cell surface interactions at the vascular wall|Integrin alphaIIb beta3 si	Fibroblasts	Monocytic lineage;Endothelial cells;Fibroblasts
COL4A5	ITGAV	0.634586466165413	4	R-HSA-194138|R-HSA-202733|R-HSA-373760	Signaling by VEGF|Cell surface interactions at the vascular wall|L1C	Neutrophils;Endothelial cells	Monocytic lineage;Neutrophils;Endothelial cells
COL4A6	ITGAV	0.688253792565801	4	R-HSA-194138|R-HSA-202733|R-HSA-373760	Signaling by VEGF|Cell surface interactions at the vascular wall|L1C	T cells;Monocytic lineage;Neutrophils;Endotl	Monocytic lineage;Neutrophils;Endothelial cells
COL5A2	ITGA1	0.533834586466165	4	R-HSA-445355	Smooth Muscle Contraction	Monocytic lineage;Endothelial cells;Fibroblas	Endothelial cells
COL6A1	ITGA1	0.514285714285714	4	R-HSA-445355	Smooth Muscle Contraction	Monocytic lineage;Endothelial cells;Fibroblas	Endothelial cells
COL6A2	ITGA1	0.559398496240601	4	R-HSA-445355	Smooth Muscle Contraction	Monocytic lineage;Myeloid dendritic cells;En	Endothelial cells
COL6A2	ITGB1	0.557894736842105	4	R-HSA-6785807	Interleukin-4 and 13 signaling	Monocytic lineage;Myeloid dendritic cells;En	Endothelial cells
COL7A1	ITGB1	0.619548872180451	4	R-HSA-6785807	Interleukin-4 and 13 signaling	NA	Endothelial cells
COL8A1	ITGA1	0.875187969924812	4	R-HSA-445355	Smooth Muscle Contraction	NA	Endothelial cells
CRH	ADCYAP1R1	0.762904169089243	4	R-HSA-166520|R-HSA-187037|R-HSA-373080	Signaling by NTRKs|Signaling by NTRK1 (TRKA)|Class B/2 (Secret	Endothelial cells	Neutrophils;Endothelial cells
CRH	PTH1R	0.686891846045114	4	R-HSA-373080	Class B/2 (Secretin family receptors)	Endothelial cells	Neutrophils;Endothelial cells
CRH	RAMP3	0.53301324085822	4	R-HSA-373080	Class B/2 (Secretin family receptors)	Endothelial cells	Endothelial cells
CTGF	ITGA5	0.742857142857143	4	R-HSA-1566977|R-HSA-202733	Fibronectin matrix formation|Cell surface interactions at the vascular	Fibroblasts	NK cells;Monocytic lineage;Endothelial cells;Fibroblasts
CXCL12	CD4	0.697744360902256	4	R-HSA-162587|R-HSA-202403|R-HSA-202424	HIV Life Cycle|TCR signaling|Downstream TCR signaling|Phosphory	T cells;Cytotoxic lymphocytes;Monocytic line	Monocytic lineage;Myeloid dendritic cells
CYR61	ITGA5	0.697744360902256	4	R-HSA-1566977|R-HSA-202733	Fibronectin matrix formation|Cell surface interactions at the vascular	Fibroblasts	NK cells;Monocytic lineage;Endothelial cells;Fibroblasts
CYR61	ITGB3	0.663157894736842	4	R-HSA-202733|R-HSA-354192|R-HSA-567413	Cell surface interactions at the vascular wall|Integrin alphaIIb beta3 si	Fibroblasts	Monocytic lineage;Endothelial cells;Fibroblasts
DKK1	KREMEN1	0.517293233082707	4	R-HSA-4791275	Signaling by WNT in cancer	NA	Monocytic lineage;Endothelial cells
DLL1	NOTCH1	0.572932330827068	4	R-HSA-157118|R-HSA-1980143|R-HSA-26446	Signaling by NOTCH|Signaling by NOTCH1|Signaling by NOTCH1	Endothelial cells	Endothelial cells
DLL1	NOTCH3	0.822556390977444	4	R-HSA-157118|R-HSA-3781865|R-HSA-90128	Signaling by NOTCH|Diseases of glycosylation|Signaling by NOTCH	Endothelial cells	Endothelial cells
DLL4	NOTCH4	0.517293233082707	4	R-HSA-3781865	Diseases of glycosylation	Endothelial cells	Endothelial cells
DSPP	ITGB1	0.518101711740638	4	R-HSA-6785807	Interleukin-4 and 13 signaling	NA	Endothelial cells
EBI3	IL27RA	0.556390977443609	4	R-HSA-447115|R-HSA-8984722|R-HSA-90209	Interleukin-12 family signaling|Interleukin-35 Signalling|Interleukin-2	NA	T cells;NK cells;Monocytic lineage
EDIL3	ITGAV	0.796992481203008	4	R-HSA-194138|R-HSA-202733|R-HSA-373760	Signaling by VEGF|Cell surface interactions at the vascular wall|L1C	T cells;Neutrophils;Endothelial cells	Monocytic lineage;Neutrophils;Endothelial cells
EDIL3	ITGB5	0.666165413533835	4	R-HSA-445355	Smooth Muscle Contraction	T cells;Neutrophils;Endothelial cells	Monocytic lineage;Endothelial cells
EFNB1	EPHB3	0.565413533834586	4	R-HSA-2682334|R-HSA-3928662|R-HSA-3928	EPH-Ephrin signaling|EPHB-mediated forward signaling|Ephrin sign	Endothelial cells	Neutrophils;Endothelial cells
EFNB1	EPHB4	0.639097744360902	4	R-HSA-2682334|R-HSA-3928665	EPH-Ephrin signaling|EPH-ephrin mediated repulsion of cells	Endothelial cells	Endothelial cells
EFNB2	EPHB3	0.840601503759399	4	R-HSA-2682334|R-HSA-3928662|R-HSA-3928	EPH-Ephrin signaling|EPHB-mediated forward signaling|Ephrin sign	Neutrophils;Endothelial cells	Neutrophils;Endothelial cells
EFNB2	EPHB4	0.616541353383459	4	R-HSA-2682334|R-HSA-3928665	EPH-Ephrin signaling|EPH-ephrin mediated repulsion of cells	Neutrophils;Endothelial cells	Endothelial cells
EFNB2	GRM1	0.751536717960897	4	R-HSA-6794362	Protein-protein interactions at synapses	Neutrophils;Endothelial cells	NA
EFNB2	GRM5	0.65813327647977	4	R-HSA-6794362	Protein-protein interactions at synapses	Neutrophils;Endothelial cells	Endothelial cells
EFNB3	EPHB3	0.855639097744361	4	R-HSA-2682334|R-HSA-3928662|R-HSA-3928	EPH-Ephrin signaling|EPHB-mediated forward signaling|Ephrin sign	Endothelial cells	Neutrophils;Endothelial cells
EFNB3	EPHB4	0.544360902255639	4	R-HSA-2682334|R-HSA-3928665	EPH-Ephrin signaling|EPH-ephrin mediated repulsion of cells	Endothelial cells	Endothelial cells
EGF	ADRB2	0.534639160527045	4	R-HSA-8856825|R-HSA-8856828	Cargo recognition for clathrin-mediated endocytosis|Clathrin-mediate	Neutrophils;Endothelial cells	NA
EGF	EGFR	0.671687508718484	4	R-HSA-1227986|R-HSA-1236394|R-HSA-1250	Signaling by ERBB2|Signaling by ERBB4|SHC1 events in ERBB2 si	Neutrophils;Endothelial cells	Neutrophils;Endothelial cells
EGF	ERBB2	0.792169573062607	4	R-HSA-199418|R-HSA-2219528|R-HSA-22195	Negative regulation of the PI3K/AKT network|PI3K/AKT Signaling i	Neutrophils;Endothelial cells	Endothelial cells
EGF	LRP2	0.70632610221742	4	R-HSA-196854|R-HSA-2187338|R-HSA-68066	Metabolism of vitamins and cofactors|Visual phototransduction|Meta	Neutrophils;Endothelial cells	Neutrophils;Endothelial cells
FASLG	FAS	0.670676691729323	4	R-HSA-109581|R-HSA-140534|R-HSA-337137	Apoptosis|Ligand-dependent caspase activation|Regulation by c-FLIP	T cells;Cytotoxic lymphocytes;NK cells;Mono	T cells;Cytotoxic lymphocytes;B lineage;NK cells;Monocytic lineage;Myeloid
FBLN1	ITGB1	0.593984962406015	4	R-HSA-6785807	Interleukin-4 and 13 signaling	NA	Endothelial cells
FBN1	ITGB1	0.645112781954887	4	R-HSA-6785807	Interleukin-4 and 13 signaling	NA	Endothelial cells
FGB	ITGAM	0.586410823488855	4	R-HSA-166016|R-HSA-168898|R-HSA-678580	Toll Like Receptor 4 (TLR4) Cascade|Toll-Like Receptors Cascades|I	NA	Monocytic lineage
FGF12	FGFR2	0.723308270676692	4	R-HSA-1226099|R-HSA-1839126|R-HSA-2033	Signaling by FGFR in disease|FGFR2 mutant receptor activation|Acti	Neutrophils;Endothelial cells	T cells;Neutrophils;Endothelial cells
FGF12	FGFR3	0.831578947368421	4	R-HSA-1226099|R-HSA-5655332|R-HSA-8853	Signaling by FGFR in disease|Signaling by FGFR3 in disease|Signali	Neutrophils;Endothelial cells	Neutrophils;Endothelial cells

FGF13	FGFR2	0.573147840456968	4	R-HSA-1226099|R-HSA-1839126|R-HSA-2033	Signaling by FGFR in disease|FGFR2 mutant receptor activation|Acti	NA	T cells;Neutrophils;Endothelial cells
FGF13	FGFR3	0.540804852084724	4	R-HSA-1226099|R-HSA-5655332|R-HSA-8853	Signaling by FGFR in disease|Signaling by FGFR3 in disease|Signali	NA	Neutrophils;Endothelial cells
FGF14	FGFR2	0.546822152247002	4	R-HSA-1226099|R-HSA-1839126|R-HSA-2033	Signaling by FGFR in disease|FGFR2 mutant receptor activation|Acti	Neutrophils;Endothelial cells	T cells;Neutrophils;Endothelial cells
FGF14	FGFR3	0.91989476230823	4	R-HSA-1226099|R-HSA-5655332|R-HSA-8853	Signaling by FGFR in disease|Signaling by FGFR3 in disease|Signali	Neutrophils;Endothelial cells	Neutrophils;Endothelial cells
FGF1	EGFR	0.596992481203008	4	R-HSA-1227986|R-HSA-1236394|R-HSA-1250	Signaling by ERBB2|Signaling by ERBB4|SHC1 events in ERBB2 si	T cells;Neutrophils;Endothelial cells	Neutrophils;Endothelial cells
FGF1	FGFR2	0.878195488721805	4	R-HSA-1226099|R-HSA-1839126|R-HSA-2033	Signaling by FGFR in disease|FGFR2 mutant receptor activation|Acti	T cells;Neutrophils;Endothelial cells	T cells;Neutrophils;Endothelial cells
FGF1	FGFR3	0.760902255639098	4	R-HSA-1226099|R-HSA-5655332|R-HSA-8853	Signaling by FGFR in disease|Signaling by FGFR3 in disease|Signali	T cells;Neutrophils;Endothelial cells	Neutrophils;Endothelial cells
FGF1	FGFRL1	0.580451127819549	4	R-HSA-190236|R-HSA-5654736	Signaling by FGFR|Signaling by FGFR1	T cells;Neutrophils;Endothelial cells	Endothelial cells
FGF20	FGFR2	0.641514001539946	4	R-HSA-1226099|R-HSA-1839126|R-HSA-2033	Signaling by FGFR in disease|FGFR2 mutant receptor activation|Acti	Neutrophils;Endothelial cells	T cells;Neutrophils;Endothelial cells
FGF20	FGFR3	0.804534030166567	4	R-HSA-1226099|R-HSA-5655332|R-HSA-8853	Signaling by FGFR in disease|Signaling by FGFR3 in disease|Signali	Neutrophils;Endothelial cells	Neutrophils;Endothelial cells
FGF22	FGFR2	0.772932330827068	4	R-HSA-1226099|R-HSA-1839126|R-HSA-2033	Signaling by FGFR in disease|FGFR2 mutant receptor activation|Acti	NA	T cells;Neutrophils;Endothelial cells
FGF2	FGFR2	0.657142857142857	4	R-HSA-1226099|R-HSA-1839126|R-HSA-2033	Signaling by FGFR in disease|FGFR2 mutant receptor activation|Acti	T cells;Monocytic lineage;Neutrophils;Endotl	T cells;Neutrophils;Endothelial cells
FGF2	FGFR3	0.607518796992481	4	R-HSA-1226099|R-HSA-5655332|R-HSA-8853	Signaling by FGFR in disease|Signaling by FGFR3 in disease|Signali	T cells;Monocytic lineage;Neutrophils;Endotl	Neutrophils;Endothelial cells
FGF2	FGFRL1	0.625563909774436	4	R-HSA-190236|R-HSA-5654736	Signaling by FGFR|Signaling by FGFR1	T cells;Monocytic lineage;Neutrophils;Endotl	Endothelial cells
FGF2	SDC2	0.705263157894737	4	R-HSA-1630316|R-HSA-1638091|R-HSA-1793	Glycosaminoglycan metabolism|Heparan sulfate/heparin (HS-GAG) r	T cells;Monocytic lineage;Neutrophils;Endotl	Neutrophils;Endothelial cells
FGF2	SDC3	0.780451127819549	4	R-HSA-1630316|R-HSA-1638091|R-HSA-1793	Glycosaminoglycan metabolism|Heparan sulfate/heparin (HS-GAG) r	T cells;Monocytic lineage;Neutrophils;Endotl	Monocytic lineage;Endothelial cells
FN1	COL13A1	0.721804511278195	4	R-HSA-1474290|R-HSA-1650814	Collagen formation|Collagen biosynthesis and modifying enzymes	Fibroblasts	T cells;Monocytic lineage;Fibroblasts
FN1	ITGA5	0.882706766917293	4	R-HSA-1566977|R-HSA-202733	Fibronectin matrix formation|Cell surface interactions at the vascular	Fibroblasts	NK cells;Monocytic lineage;Endothelial cells;Fibroblasts
FN1	ITGB3	0.529323308270677	4	R-HSA-202733|R-HSA-354192|R-HSA-567413	Cell surface interactions at the vascular wall|Integrin alphaIIb beta3 si	Fibroblasts	Monocytic lineage;Endothelial cells;Fibroblasts
GAS6	AXL	0.894736842105263	4	R-HSA-194138|R-HSA-4420097	Signaling by VEGF|VEGFA-VEGFR2 Pathway	Monocytic lineage;Neutrophils;Endothelial ce	T cells;Monocytic lineage;Endothelial cells
GDF11	ACVR2B	0.580451127819549	4	R-HSA-201451|R-HSA-9006936	Signaling by BMP|Signaling by TGF-beta family members	NA	NA
GDF11	BMPR1B	0.581954887218045	4	R-HSA-9006936	Signaling by TGF-beta family members	NA	Neutrophils;Endothelial cells
GDF5	BMPR1B	0.618045112781955	4	R-HSA-9006936	Signaling by TGF-beta family members	NA	Neutrophils;Endothelial cells
GDNF	GFRA1	0.661654135338346	4	R-HSA-8853659	RET signaling	Neutrophils;Endothelial cells	NA
GNAI2	ADCY7	0.675187969924812	4	R-HSA-163685|R-HSA-418597	Integration of energy metabolism|G alpha (z) signalling events	Monocytic lineage	T cells;Monocytic lineage;Myeloid dendritic cells;Neutrophils
GNAI2	CCR5	0.661654135338346	4	R-HSA-162587|R-HSA-6783783	HIV Life Cycle|Interleukin-10 signaling	Monocytic lineage	T cells;Cytotoxic lymphocytes;NK cells;Monocytic lineage;Neutrophils
GNAI2	FPR1	0.774436090225564	4	R-HSA-6783783	Interleukin-10 signaling	Monocytic lineage	Monocytic lineage
GNAI2	PTPRU	0.542857142857143	4	R-HSA-1433557	Signaling by SCF-KIT	Monocytic lineage	Endothelial cells
GNAI2	S1PR1	0.523308270676692	4	R-HSA-6785807	Interleukin-4 and 13 signaling	Monocytic lineage	Neutrophils;Endothelial cells
GPC3	IGF1R	0.687476543540247	4	R-HSA-2404192|R-HSA-2428924|R-HSA-2428	Signaling by Type 1 Insulin-like Growth Factor 1 Receptor (IGF1R)|I	Neutrophils;Endothelial cells	Endothelial cells
GPC6	LRP1	0.610526315789474	4	R-HSA-196854|R-HSA-2187338|R-HSA-68066	Metabolism of vitamins and cofactors|Visual phototransduction|Meta	Endothelial cells	Monocytic lineage;Endothelial cells
HBEGF	EGFR	0.584962406015038	4	R-HSA-1227986|R-HSA-1236394|R-HSA-1250	Signaling by ERBB2|Signaling by ERBB4|SHC1 events in ERBB2 si	NA	Neutrophils;Endothelial cells
HGF	ITGB1	0.619548872180451	4	R-HSA-6785807	Interleukin-4 and 13 signaling	Monocytic lineage;Endothelial cells	Endothelial cells
HGF	SDC2	0.830075187969925	4	R-HSA-1630316|R-HSA-1638091|R-HSA-1793	Glycosaminoglycan metabolism|Heparan sulfate/heparin (HS-GAG) r	Monocytic lineage;Endothelial cells	Neutrophils;Endothelial cells
HLA-A	CD3D	0.830075187969925	4	R-HSA-202403|R-HSA-202424|R-HSA-202427	TCR signaling|Downstream TCR signaling|Phosphorylation of CD3 a	T cells;Cytotoxic lymphocytes;NK cells;Mono	T cells;Cytotoxic lymphocytes;NK cells;Monocytic lineage;Neutrophils
HLA-A	CD3G	0.837593984962406	4	R-HSA-198933|R-HSA-202403|R-HSA-202424	Immunoregulatory interactions between a Lymphoid and a non-Lymp	T cells;Cytotoxic lymphocytes;NK cells;Mono	T cells;Cytotoxic lymphocytes;NK cells;Monocytic lineage
HLA-A	LILRB2	0.830075187969925	4	R-HSA-198933	Immunoregulatory interactions between a Lymphoid and a non-Lymp	T cells;Cytotoxic lymphocytes;NK cells;Mono	T cells;Cytotoxic lymphocytes;NK cells;Monocytic lineage
HLA-B	CD3D	0.760902255639098	4	R-HSA-202403|R-HSA-202424|R-HSA-202427	TCR signaling|Downstream TCR signaling|Phosphorylation of CD3 a	T cells;Cytotoxic lymphocytes;Monocytic line	T cells;Cytotoxic lymphocytes;NK cells;Monocytic lineage;Neutrophils
HLA-B	CD3G	0.778947368421053	4	R-HSA-198933|R-HSA-202403|R-HSA-202424	Immunoregulatory interactions between a Lymphoid and a non-Lymp	T cells;Cytotoxic lymphocytes;Monocytic line	T cells;Cytotoxic lymphocytes;NK cells;Monocytic lineage
HLA-B	LILRB2	0.711278195488722	4	R-HSA-198933	Immunoregulatory interactions between a Lymphoid and a non-Lymp	T cells;Cytotoxic lymphocytes;Monocytic line	T cells;Cytotoxic lymphocytes;NK cells;Monocytic lineage
HLA-C	CD3D	0.735338345864662	4	R-HSA-202403|R-HSA-202424|R-HSA-202427	TCR signaling|Downstream TCR signaling|Phosphorylation of CD3 a	T cells;Cytotoxic lymphocytes;Monocytic line	T cells;Cytotoxic lymphocytes;NK cells;Monocytic lineage;Neutrophils
HLA-C	CD3G	0.77593984962406	4	R-HSA-198933|R-HSA-202403|R-HSA-202424	Immunoregulatory interactions between a Lymphoid and a non-Lymp	T cells;Cytotoxic lymphocytes;Monocytic line	T cells;Cytotoxic lymphocytes;NK cells;Monocytic lineage
HLA-C	LILRB2	0.798496240601504	4	R-HSA-198933	Immunoregulatory interactions between a Lymphoid and a non-Lymp	T cells;Cytotoxic lymphocytes;Monocytic line	T cells;Cytotoxic lymphocytes;NK cells;Monocytic lineage
HRAS	AGTR1	0.654564238224007	4	R-HSA-8856825|R-HSA-8856828	Cargo recognition for clathrin-mediated endocytosis|Clathrin-mediate	Endothelial cells	NA
HRAS	INSR	0.747368421052632	4	R-HSA-199418|R-HSA-6811558|R-HSA-74751	Negative regulation of the PI3K/AKT network|PI5P, PP2A and IER3	Endothelial cells	Neutrophils;Endothelial cells
HRAS	SDC2	0.583458646616541	4	R-HSA-1630316|R-HSA-1638091|R-HSA-1793	Glycosaminoglycan metabolism|Heparan sulfate/heparin (HS-GAG) r	Endothelial cells	Neutrophils;Endothelial cells
HSPG2	COL13A1	0.754887218045113	4	R-HSA-1474290|R-HSA-1650814	Collagen formation|Collagen biosynthesis and modifying enzymes	Endothelial cells;Fibroblasts	T cells;Monocytic lineage;Fibroblasts
HSPG2	ITGB1	0.53984962406015	4	R-HSA-6785807	Interleukin-4 and 13 signaling	Endothelial cells;Fibroblasts	Endothelial cells
ICAM1	IL2RA	0.721804511278195	4	R-HSA-392451|R-HSA-397795|R-HSA-451927	G beta:gamma signalling through PI3Kgamma|G-protein beta:gamma	T cells;Cytotoxic lymphocytes;NK cells;Mono	T cells;B lineage
ICAM1	IL2RG	0.765413533834587	4	R-HSA-1266695|R-HSA-392451|R-HSA-39779	Interleukin-7 signaling|G beta:gamma signalling through PI3Kgamma	T cells;Cytotoxic lymphocytes;NK cells;Mono	B lineage;NK cells;Monocytic lineage;Fibroblasts
ICAM1	ITGAL	0.869172932330827	4	R-HSA-202733|R-HSA-8878159	Cell surface interactions at the vascular wall|Transcriptional regulatio	T cells;Cytotoxic lymphocytes;NK cells;Mono	T cells;Cytotoxic lymphocytes;NK cells;Monocytic lineage;Myeloid dendritic c
ICAM1	ITGAX	0.553383458646617	4	R-HSA-6785807	Interleukin-4 and 13 signaling	T cells;Cytotoxic lymphocytes;NK cells;Mono	Monocytic lineage;Myeloid dendritic cells;Fibroblasts
ICAM1	ITGB2	0.711278195488722	4	R-HSA-166016|R-HSA-168898|R-HSA-202733	Toll Like Receptor 4 (TLR4) Cascade|Toll-Like Receptors Cascades|	T cells;Cytotoxic lymphocytes;NK cells;Mono	T cells;NK cells;Monocytic lineage;Myeloid dendritic cells;Neutrophils
ICAM4	ITGAL	0.529323308270677	4	R-HSA-202733|R-HSA-8878159	Cell surface interactions at the vascular wall|Transcriptional regulatio	NA	T cells;Cytotoxic lymphocytes;NK cells;Monocytic lineage;Myeloid dendritic c
ICAM4	ITGAM	0.542857142857143	4	R-HSA-166016|R-HSA-168898|R-HSA-678580	Toll Like Receptor 4 (TLR4) Cascade|Toll-Like Receptors Cascades|	NA	Monocytic lineage
ICAM4	ITGB2	0.625563909774436	4	R-HSA-166016|R-HSA-168898|R-HSA-202733	Toll Like Receptor 4 (TLR4) Cascade|Toll-Like Receptors Cascades|	NA	T cells;NK cells;Monocytic lineage;Myeloid dendritic cells;Neutrophils
ICAM4	ITGB3	0.536842105263158	4	R-HSA-202733|R-HSA-354192|R-HSA-567413	Cell surface interactions at the vascular wall|Integrin alphaIIb beta3 si	NA	Monocytic lineage;Endothelial cells;Fibroblasts
IFNG	IFNGR2	0.628571428571429	4	R-HSA-877300	Interferon gamma signaling	T cells;Cytotoxic lymphocytes;NK cells;Mono	T cells;Cytotoxic lymphocytes;NK cells;Monocytic lineage;Myeloid dendritic c
IGF2	IGF1R	0.654135338345865	4	R-HSA-2404192|R-HSA-2428924|R-HSA-2428	Signaling by Type 1 Insulin-like Growth Factor 1 Receptor (IGF1R)|I	Endothelial cells	Endothelial cells
IGF2	INSR	0.66015037593985	4	R-HSA-199418|R-HSA-6811558|R-HSA-74751	Negative regulation of the PI3K/AKT network|PI5P, PP2A and IER3	Endothelial cells	Neutrophils;Endothelial cells
IL10	IL10RB	0.645112781954887	4	R-HSA-6783783|R-HSA-8854691	Interleukin-10 signaling|Interleukin-20 family signaling	B lineage	NA
IL10	SIRPG	0.541353383458647	4	R-HSA-202733	Cell surface interactions at the vascular wall	B lineage	T cells;Cytotoxic lymphocytes;NK cells;Monocytic lineage
IL12B	IL23R	0.683114450704478	4	R-HSA-447115|R-HSA-6785807	Interleukin-12 family signaling|Interleukin-4 and 13 signaling	NA	NA
IL18	IL18BP	0.801503759398496	4	R-HSA-446652	Interleukin-1 family signaling	T cells;Cytotoxic lymphocytes;Monocytic line	T cells;Cytotoxic lymphocytes;NK cells;Monocytic lineage;Myeloid dendritic c
IL18	IL18R1	0.520300751879699	4	R-HSA-446652	Interleukin-1 family signaling	T cells;Cytotoxic lymphocytes;Monocytic line	NA
IL18	IL18RAP	0.690225563909774	4	R-HSA-446652	Interleukin-1 family signaling	T cells;Cytotoxic lymphocytes;Monocytic line	Monocytic lineage
IL1RN	IL1R2	0.601503759398496	4	R-HSA-446652|R-HSA-6783783|R-HSA-90207	Interleukin-1 family signaling|Interleukin-10 signaling|Interleukin-1 s	T cells	T cells

IL27	IL27RA	0.519578902484029	4	R-HSA-447115|R-HSA-8984722|R-HSA-90209	Interleukin-12 family signaling|Interleukin-35 Signalling|Interleukin-	T cells;Cytotoxic lymphocytes;NK cells;Neut	T cells;NK cells;Monocytic lineage
INHBB	ACVR2B	0.559398496240601	4	R-HSA-201451|R-HSA-9006936	Signaling by BMP|Signaling by TGF-beta family members	Endothelial cells	NA
IRAK4	TLR6	0.545864661654135	4	R-HSA-166016|R-HSA-166058|R-HSA-168179	Toll Like Receptor 4 (TLR4) Cascade|MyD88:Mal cascade initiated c	NA	NA
JAG1	NOTCH1	0.58796992481203	4	R-HSA-157118|R-HSA-1980143|R-HSA-26446	Signaling by NOTCH|Signaling by NOTCH1|Signaling by NOTCH1	Endothelial cells	Endothelial cells
JAG1	NOTCH2	0.708270676691729	4	R-HSA-157118|R-HSA-3781865	Signaling by NOTCH|Diseases of glycosylation	Endothelial cells	Endothelial cells;Fibroblasts
JAG1	NOTCH3	0.915789473684211	4	R-HSA-157118|R-HSA-3781865|R-HSA-90128	Signaling by NOTCH|Diseases of glycosylation|Signaling by NOTCH	Endothelial cells	Endothelial cells
JAG2	NOTCH1	0.694736842105263	4	R-HSA-157118|R-HSA-1980143|R-HSA-26446	Signaling by NOTCH|Signaling by NOTCH1|Signaling by NOTCH1	Endothelial cells	Endothelial cells
JAG2	NOTCH3	0.708270676691729	4	R-HSA-157118|R-HSA-3781865|R-HSA-90128	Signaling by NOTCH|Diseases of glycosylation|Signaling by NOTCH	Endothelial cells	Endothelial cells
KITLG	KIT	0.83609022556391	4	R-HSA-1433557|R-HSA-199418|R-HSA-22195	Signaling by SCF-KIT|Negative regulation of the PI3K/AKT network	Neutrophils;Endothelial cells	Neutrophils;Endothelial cells
LAMA1	GPC1	0.735338345864662	4	R-HSA-1630316|R-HSA-1638091|R-HSA-1793	Glycosaminoglycan metabolism|Heparan sulfate/heparin (HS-GAG) r	Neutrophils;Endothelial cells	Neutrophils;Endothelial cells
LAMA1	ITGA6	0.810526315789474	4	R-HSA-1474290|R-HSA-1500931|R-HSA-2022	Collagen formation|Cell-Cell communication|Assembly of collagen fi	Neutrophils;Endothelial cells	Neutrophils;Endothelial cells
LAMA1	ITGB4	0.548872180451128	4	R-HSA-1474290	Collagen formation	Neutrophils;Endothelial cells	Endothelial cells
LAMA1	SDC2	0.879699248120301	4	R-HSA-1630316|R-HSA-1638091|R-HSA-1793	Glycosaminoglycan metabolism|Heparan sulfate/heparin (HS-GAG) r	Neutrophils;Endothelial cells	Neutrophils;Endothelial cells
LAMA3	SDC2	0.616541353383459	4	R-HSA-1630316|R-HSA-1638091|R-HSA-1793	Glycosaminoglycan metabolism|Heparan sulfate/heparin (HS-GAG) r	Endothelial cells	Neutrophils;Endothelial cells
LAMA4	ITGB1	0.651127819548872	4	R-HSA-6785807	Interleukin-4 and 13 signaling	Endothelial cells;Fibroblasts	Endothelial cells
LAMA5	ITGA6	0.550375939849624	4	R-HSA-1474290|R-HSA-1500931|R-HSA-2022	Collagen formation|Cell-Cell communication|Assembly of collagen fi	Endothelial cells	Neutrophils;Endothelial cells
LAMB1	ITGA1	0.581954887218045	4	R-HSA-445355	Smooth Muscle Contraction	Monocytic lineage;Fibroblasts	Endothelial cells
LAMB1	ITGB1	0.699248120300752	4	R-HSA-6785807	Interleukin-4 and 13 signaling	Monocytic lineage;Fibroblasts	Endothelial cells
LAMB2	ITGB1	0.589473684210526	4	R-HSA-6785807	Interleukin-4 and 13 signaling	Endothelial cells	Endothelial cells
LAMC3	ITGB1	0.55187969924812	4	R-HSA-6785807	Interleukin-4 and 13 signaling	NA	Endothelial cells
LGALS9	HAVCR2	0.756390977443609	4	R-HSA-451927	Interleukin-2 family signaling	T cells;Monocytic lineage;Myeloid dendritic	T cells;Cytotoxic lymphocytes;Monocytic lineage
LIPC	LRP1	0.807518796992481	4	R-HSA-196854|R-HSA-2187338|R-HSA-68066	Metabolism of vitamins and cofactors|Visual phototransduction|Meta	Monocytic lineage;Endothelial cells	Monocytic lineage;Endothelial cells
LRPAP1	LRP1	0.801503759398496	4	R-HSA-196854|R-HSA-2187338|R-HSA-68066	Metabolism of vitamins and cofactors|Visual phototransduction|Meta	Monocytic lineage;Endothelial cells	Monocytic lineage;Endothelial cells
LTA	TNFRSF14	0.622556390977444	4	R-HSA-388841|R-HSA-5668541	Costimulation by the CD28 family|TNFR2 non-canonical NF-kB pat	B lineage	T cells;Cytotoxic lymphocytes;NK cells;Monocytic lineage;Myeloid dendritic
LTBP1	ITGB5	0.627067669172932	4	R-HSA-445355	Smooth Muscle Contraction	Endothelial cells	Monocytic lineage;Endothelial cells
LTBP3	ITGB5	0.950375939849624	4	R-HSA-445355	Smooth Muscle Contraction	Monocytic lineage;Endothelial cells	Monocytic lineage;Endothelial cells
LTF	LRP1	0.530827067669173	4	R-HSA-196854|R-HSA-2187338|R-HSA-68066	Metabolism of vitamins and cofactors|Visual phototransduction|Meta	T cells;Neutrophils;Endothelial cells	Monocytic lineage;Endothelial cells
LYZ	ITGAL	0.708270676691729	4	R-HSA-202733|R-HSA-8878159	Cell surface interactions at the vascular wall|Transcriptional regulatio	T cells;Cytotoxic lymphocytes;Monocytic lin	T cells;Cytotoxic lymphocytes;NK cells;Monocytic lineage;Myeloid dendritic
MDK	LRP1	0.544360902255639	4	R-HSA-196854|R-HSA-2187338|R-HSA-68066	Metabolism of vitamins and cofactors|Visual phototransduction|Meta	Endothelial cells	Monocytic lineage;Endothelial cells
MFGE8	ITGAV	0.593984962406015	4	R-HSA-194138|R-HSA-202733|R-HSA-373760	Signaling by VEGF|Cell surface interactions at the vascular wall|L1C	Endothelial cells	Monocytic lineage;Neutrophils;Endothelial cells
MFGE8	ITGB3	0.589473684210526	4	R-HSA-202733|R-HSA-354192|R-HSA-567413	Cell surface interactions at the vascular wall|Integrin alphaIIb beta3 s	Endothelial cells	Monocytic lineage;Endothelial cells;Fibroblasts
MFGE8	PDGFRB	0.618045112781955	4	R-HSA-199418|R-HSA-2219528|R-HSA-22195	Negative regulation of the PI3K/AKT network|PI3K/AKT Signaling i	Endothelial cells	Endothelial cells;Fibroblasts
MLLT4	EPHB3	0.536842105263158	4	R-HSA-2682334|R-HSA-3928662|R-HSA-3928	EPH-Ephrin signaling|EPHB-mediated forward signaling|Ephrin sign	NA	Neutrophils;Endothelial cells
MMP9	CD44	0.708270676691729	4	R-HSA-1474228|R-HSA-202733	Degradation of the extracellular matrix|Cell surface interactions at the	Monocytic lineage;Myeloid dendritic cells;Fi	Monocytic lineage;Fibroblasts
MMP9	ITGB2	0.533834586466165	4	R-HSA-166016|R-HSA-168898|R-HSA-202733	Toll Like Receptor 4 (TLR4) Cascade|Toll-Like Receptors Cascades|	Monocytic lineage;Myeloid dendritic cells;Fi	T cells;NK cells;Monocytic lineage;Myeloid dendritic cells;Neutrophils
MSTN	ACVR2B	0.602482178748072	4	R-HSA-201451|R-HSA-9006936	Signaling by BMP|Signaling by TGF-beta family members	NA	NA
NCAM1	FGFR2	0.876691729323308	4	R-HSA-1226099|R-HSA-1839126|R-HSA-2033	Signaling by FGFR in disease|FGFR2 mutant receptor activation|Acti	Neutrophils;Endothelial cells	T cells;Neutrophils;Endothelial cells
NCAM1	GFRA1	0.891729323308271	4	R-HSA-8853659	RET signaling	Neutrophils;Endothelial cells	NA
NCAN	CDH2	0.774436090225564	4	R-HSA-375170|R-HSA-381426|R-HSA-525793	CDO in myogenesis|Regulation of Insulin-like Growth Factor (IGF) t	Neutrophils;Endothelial cells	Neutrophils;Endothelial cells
NCAN	SDC3	0.651127819548872	4	R-HSA-1630316|R-HSA-1638091|R-HSA-1793	Glycosaminoglycan metabolism|Heparan sulfate/heparin (HS-GAG) r	Neutrophils;Endothelial cells	Monocytic lineage;Endothelial cells
NDP	LGR4	0.765413533834587	4	R-HSA-4641263	Regulation of FZD by ubiquitination	Neutrophils;Endothelial cells;Fibroblasts	Endothelial cells
NID1	COL13A1	0.595488721804511	4	R-HSA-1474290|R-HSA-1650814	Collagen formation|Collagen biosynthesis and modifying enzymes	Endothelial cells;Fibroblasts	T cells;Monocytic lineage;Fibroblasts
NID1	ITGB1	0.714285714285714	4	R-HSA-6785807	Interleukin-4 and 13 signaling	Endothelial cells;Fibroblasts	Endothelial cells
NID1	ITGB3	0.640601503759399	4	R-HSA-202733|R-HSA-354192|R-HSA-567413	Cell surface interactions at the vascular wall|Integrin alphaIIb beta3 s	Endothelial cells;Fibroblasts	Monocytic lineage;Endothelial cells;Fibroblasts
NID2	COL13A1	0.639097744360902	4	R-HSA-1474290|R-HSA-1650814	Collagen formation|Collagen biosynthesis and modifying enzymes	Endothelial cells;Fibroblasts	T cells;Monocytic lineage;Fibroblasts
NLGN1	NRXN1	0.770214420771568	4	R-HSA-6794362	Protein-protein interactions at synapses	Neutrophils;Endothelial cells	Endothelial cells
NLGN1	NRXN2	0.951485588160189	4	R-HSA-6794362	Protein-protein interactions at synapses	Neutrophils;Endothelial cells	Neutrophils;Endothelial cells
NLGN1	NRXN3	0.913125299625668	4	R-HSA-6794362	Protein-protein interactions at synapses	Neutrophils;Endothelial cells	Neutrophils;Endothelial cells
NLGN2	NRXN1	0.630075187969925	4	R-HSA-6794362	Protein-protein interactions at synapses	Neutrophils;Endothelial cells	Endothelial cells
NLGN2	NRXN2	0.914285714285714	4	R-HSA-6794362	Protein-protein interactions at synapses	Neutrophils;Endothelial cells	Neutrophils;Endothelial cells
NLGN2	NRXN3	0.884210526315789	4	R-HSA-6794362	Protein-protein interactions at synapses	Neutrophils;Endothelial cells	Neutrophils;Endothelial cells
NLGN3	NRXN1	0.673684210526316	4	R-HSA-6794362	Protein-protein interactions at synapses	Neutrophils;Endothelial cells	Endothelial cells
NLGN3	NRXN2	0.912781954887218	4	R-HSA-6794362	Protein-protein interactions at synapses	Neutrophils;Endothelial cells	Neutrophils;Endothelial cells
NLGN3	NRXN3	0.869172932330827	4	R-HSA-6794362	Protein-protein interactions at synapses	Neutrophils;Endothelial cells	Neutrophils;Endothelial cells
NTN1	NEO1	0.747368421052632	4	R-HSA-375170|R-HSA-525793	CDO in myogenesis|Myogenesis	Neutrophils;Endothelial cells	T cells;Neutrophils;Endothelial cells
NTN1	UNC5B	0.627067669172932	4	R-HSA-109581|R-HSA-373752|R-HSA-535780	Apoptosis|Netrin-1 signaling|Programmed Cell Death	Neutrophils;Endothelial cells	T cells;Neutrophils;Endothelial cells;Fibroblasts
NXPH1	NRXN1	0.783133418236798	4	R-HSA-6794362	Protein-protein interactions at synapses	Neutrophils;Endothelial cells	Endothelial cells
NXPH1	NRXN2	0.816265985931431	4	R-HSA-6794362	Protein-protein interactions at synapses	Neutrophils;Endothelial cells	Neutrophils;Endothelial cells
NXPH1	NRXN3	0.727410463477641	4	R-HSA-6794362	Protein-protein interactions at synapses	Neutrophils;Endothelial cells	Neutrophils;Endothelial cells
NXPH3	NRXN1	0.6	4	R-HSA-6794362	Protein-protein interactions at synapses	Neutrophils;Endothelial cells	Endothelial cells
NXPH3	NRXN2	0.827067669172932	4	R-HSA-6794362	Protein-protein interactions at synapses	Neutrophils;Endothelial cells	Neutrophils;Endothelial cells
NXPH3	NRXN3	0.827067669172932	4	R-HSA-6794362	Protein-protein interactions at synapses	Neutrophils;Endothelial cells	Neutrophils;Endothelial cells
OMG	LINGO1	0.732330827067669	4	R-HSA-73887	Death Receptor Signalling	Neutrophils;Endothelial cells	Neutrophils;Endothelial cells
OMG	RTN4RL1	0.690225563909774	4	R-HSA-163125	Post-translational modification: synthesis of GPI-anchored proteins	Neutrophils;Endothelial cells	Neutrophils;Endothelial cells
OSM	OSMR	0.533834586466165	4	R-HSA-6783589	Interleukin-6 family signaling	T cells	T cells;Monocytic lineage;Myeloid dendritic cells;Endothelial cells;Fibroblasts
PDGFB	ITGAV	0.86015037593985	4	R-HSA-194138|R-HSA-202733|R-HSA-373760	Signaling by VEGF|Cell surface interactions at the vascular wall|L1C	Monocytic lineage;Endothelial cells	Monocytic lineage;Neutrophils;Endothelial cells
PDGFB	LRP1	0.876691729323308	4	R-HSA-196854|R-HSA-2187338|R-HSA-68066	Metabolism of vitamins and cofactors|Visual phototransduction|Meta	Monocytic lineage;Endothelial cells	Monocytic lineage;Endothelial cells

PDGFB	PDGFRA	0.765413533834587	4	R-HSA-199418|R-HSA-2219528|R-HSA-22195	Negative regulation of the PI3K/AKT network|PI3K/AKT Signaling i	Monocytic lineage;Endothelial cells	Endothelial cells
PDGFB	S1PR1	0.714285714285714	4	R-HSA-6785807	Interleukin-4 and 13 signaling	Monocytic lineage;Endothelial cells	Neutrophils;Endothelial cells
PDGFC	FLT1	0.637593984962406	4	R-HSA-194138	Signaling by VEGF	Endothelial cells	Endothelial cells
PDGFC	FLT4	0.793984962406015	4	R-HSA-194138	Signaling by VEGF	Endothelial cells	Endothelial cells
PDGFC	KDR	0.790977443609023	4	R-HSA-194138|R-HSA-4420097	Signaling by VEGF|VEGFA-VEGFR2 Pathway	Endothelial cells	Endothelial cells
PDGFC	PDGFRA	0.766917293233083	4	R-HSA-199418|R-HSA-2219528|R-HSA-22195	Negative regulation of the PI3K/AKT network|PI3K/AKT Signaling i	Endothelial cells	Endothelial cells
PGF	FLT1	0.511278195488722	4	R-HSA-194138	Signaling by VEGF	Endothelial cells;Fibroblasts	Endothelial cells
PGF	NRP1	0.62406015037594	4	R-HSA-194138|R-HSA-373755|R-HSA-373760	Signaling by VEGF|Semaphorin interactions|L1CAM interactions	Endothelial cells;Fibroblasts	Endothelial cells
PKM	CD44	0.670676691729323	4	R-HSA-1474228|R-HSA-202733	Degradation of the extracellular matrix|Cell surface interactions at the	B lineage;Monocytic lineage;Fibroblasts	Monocytic lineage;Fibroblasts
PLAT	ITGAM	0.714285714285714	4	R-HSA-166016|R-HSA-168898|R-HSA-678580	Toll Like Receptor 4 (TLR4) Cascade|Toll-Like Receptors Cascades|I	Endothelial cells	Monocytic lineage
PLAT	LRP1	0.762406015037594	4	R-HSA-196854|R-HSA-2187338|R-HSA-68066	Metabolism of vitamins and cofactors|Visual phototransduction|Meta	Endothelial cells	Monocytic lineage;Endothelial cells
PLAU	IGF2R	0.678195488721804	4	R-HSA-199992|R-HSA-421837|R-HSA-432722	trans-Golgi Network Vesicle Budding|Clathrin derived vesicle buddir	T cells;Monocytic lineage;Myeloid dendritic	NK cells;Monocytic lineage;Fibroblasts
PLAU	ITGA5	0.714285714285714	4	R-HSA-1566977|R-HSA-202733	Fibronectin matrix formation|Cell surface interactions at the vascular	T cells;Monocytic lineage;Myeloid dendritic	NK cells;Monocytic lineage;Endothelial cells;Fibroblasts
PLAU	ITGB2	0.741353383458647	4	R-HSA-166016|R-HSA-168898|R-HSA-202733	Toll Like Receptor 4 (TLR4) Cascade|Toll-Like Receptors Cascades|C	T cells;Monocytic lineage;Myeloid dendritic	T cells;NK cells;Monocytic lineage;Myeloid dendritic cells;Neutrophils
PLTP	ABCA1	0.538345864661654	4	R-HSA-1989781|R-HSA-400206	PPARA activates gene expression|Regulation of lipid metabolism by	T cells	T cells;Monocytic lineage
PROS1	AXL	0.768421052631579	4	R-HSA-194138|R-HSA-4420097	Signaling by VEGF|VEGFA-VEGFR2 Pathway	Monocytic lineage;Endothelial cells	T cells;Monocytic lineage;Endothelial cells
PSAP	LRP1	0.721804511278195	4	R-HSA-196854|R-HSA-2187338|R-HSA-68066	Metabolism of vitamins and cofactors|Visual phototransduction|Meta	Monocytic lineage	Monocytic lineage;Endothelial cells
PTN	PTPRS	0.515789473684211	4	R-HSA-6794362	Protein-protein interactions at synapses	T cells;Neutrophils;Endothelial cells	Endothelial cells
PTN	SDC3	0.781954887218045	4	R-HSA-1630316|R-HSA-1638091|R-HSA-1793	Glycosaminoglycan metabolism|Heparan sulfate/heparin (HS-GAG) r	T cells;Neutrophils;Endothelial cells	Monocytic lineage;Endothelial cells
RGMA	BMPR1B	0.866165413533835	4	R-HSA-9006936	Signaling by TGF-beta family members	Neutrophils;Endothelial cells	Neutrophils;Endothelial cells
RGMA	NEO1	0.897744360902256	4	R-HSA-375170|R-HSA-525793	CDO in myogenesis|Myogenesis	Neutrophils;Endothelial cells	T cells;Neutrophils;Endothelial cells
RNASE2	TLR2	0.572932330827068	4	R-HSA-166016|R-HSA-166058|R-HSA-168179	Toll Like Receptor 4 (TLR4) Cascade|MyD88:Mal cascade initiated c	Endothelial cells	T cells;Monocytic lineage;Neutrophils
RSPO3	SDC4	0.621052631578947	4	R-HSA-202733|R-HSA-3781865	Cell surface interactions at the vascular wall|Diseases of glycosylatior	T cells;NK cells;Monocytic lineage;Myeloid c	Monocytic lineage;Fibroblasts
RTN4	LINGO1	0.84812030075188	4	R-HSA-73887	Death Receptor Signalling	T cells;Monocytic lineage;Neutrophils;Endotl	Neutrophils;Endothelial cells
RTN4	RTN4RL1	0.628571428571429	4	R-HSA-163125	Post-translational modification: synthesis of GPI-anchored proteins	T cells;Monocytic lineage;Neutrophils;Endotl	Neutrophils;Endothelial cells
SELPLG	ITGAM	0.633082706766917	4	R-HSA-166016|R-HSA-168898|R-HSA-678580	Toll Like Receptor 4 (TLR4) Cascade|Toll-Like Receptors Cascades|I	NA	Monocytic lineage
SELPLG	ITGB2	0.521804511278195	4	R-HSA-166016|R-HSA-168898|R-HSA-202733	Toll Like Receptor 4 (TLR4) Cascade|Toll-Like Receptors Cascades|C	NA	T cells;NK cells;Monocytic lineage;Myeloid dendritic cells;Neutrophils
SEMA6D	TREM2	0.720300751879699	4	R-HSA-373755	Semaphorin interactions	Neutrophils;Endothelial cells	Monocytic lineage;Neutrophils;Endothelial cells
SERPINA1	LRP1	0.526315789473684	4	R-HSA-196854|R-HSA-2187338|R-HSA-68066	Metabolism of vitamins and cofactors|Visual phototransduction|Meta	T cells;Cytotoxic lymphocytes;NK cells;Mon	Monocytic lineage;Endothelial cells
SERPINE2	LRP1	0.663157894736842	4	R-HSA-196854|R-HSA-2187338|R-HSA-68066	Metabolism of vitamins and cofactors|Visual phototransduction|Meta	Endothelial cells	Monocytic lineage;Endothelial cells
SLIT2	GPC1	0.673684210526316	4	R-HSA-1630316|R-HSA-1638091|R-HSA-1793	Glycosaminoglycan metabolism|Heparan sulfate/heparin (HS-GAG) r	Endothelial cells	Neutrophils;Endothelial cells
SORBS1	ITGB5	0.697744360902256	4	R-HSA-445355	Smooth Muscle Contraction	Neutrophils;Endothelial cells	Monocytic lineage;Endothelial cells
SPP1	ITGA9	0.52781954887218	4	R-HSA-373760	L1CAM interactions	Endothelial cells;Fibroblasts	Monocytic lineage;Endothelial cells
SPP1	ITGAV	0.526315789473684	4	R-HSA-194138|R-HSA-202733|R-HSA-373760	Signaling by VEGF|Cell surface interactions at the vascular wall|L1C	Endothelial cells;Fibroblasts	Monocytic lineage;Neutrophils;Endothelial cells
SPP1	ITGB5	0.556390977443609	4	R-HSA-445355	Smooth Muscle Contraction	Endothelial cells;Fibroblasts	Monocytic lineage;Endothelial cells
TDGF1	ACVR1C	0.582315930261588	4	R-HSA-9006936	Signaling by TGF-beta family members	Neutrophils;Endothelial cells	Neutrophils;Endothelial cells;Fibroblasts
TDGF1	GPC1	0.643774075935634	4	R-HSA-1630316|R-HSA-1638091|R-HSA-1793	Glycosaminoglycan metabolism|Heparan sulfate/heparin (HS-GAG) r	Neutrophils;Endothelial cells	Neutrophils;Endothelial cells
TFPI	SDC4	0.61203007518797	4	R-HSA-202733|R-HSA-3781865	Cell surface interactions at the vascular wall|Diseases of glycosylatior	Fibroblasts	Monocytic lineage;Fibroblasts
TF	LRP2	0.83609022556391	4	R-HSA-196854|R-HSA-2187338|R-HSA-68066	Metabolism of vitamins and cofactors|Visual phototransduction|Meta	Neutrophils;Endothelial cells	Neutrophils;Endothelial cells
TGFA	EGFR	0.827067669172932	4	R-HSA-1227986|R-HSA-1236394|R-HSA-1250	Signaling by ERBB2|Signaling by ERBB4|SHC1 events in ERBB2 si	Neutrophils;Endothelial cells	Neutrophils;Endothelial cells
TGFA	ERBB2	0.630075187969925	4	R-HSA-199418|R-HSA-2219528|R-HSA-22195	Negative regulation of the PI3K/AKT network|PI3K/AKT Signaling i	Neutrophils;Endothelial cells	Endothelial cells
TGFB1	ITGB3	0.544360902255639	4	R-HSA-202733|R-HSA-354192|R-HSA-567413	Cell surface interactions at the vascular wall|Integrin alphaIIb beta3 si	NK cells;Monocytic lineage;Neutrophils	Monocytic lineage;Endothelial cells;Fibroblasts
TGFB2	TGFBR1	0.690225563909774	4	R-HSA-170834|R-HSA-5689880|R-HSA-90069	Signaling by TGF-beta Receptor Complex|Ub-specific processing prc	Endothelial cells	T cells;Monocytic lineage;Neutrophils;Endothelial cells;Fibroblasts
TGFB3	ITGB5	0.881203007518797	4	R-HSA-445355	Smooth Muscle Contraction	Endothelial cells	Monocytic lineage;Endothelial cells
TGFB3	TGFBR1	0.616541353383459	4	R-HSA-170834|R-HSA-5689880|R-HSA-90069	Signaling by TGF-beta Receptor Complex|Ub-specific processing prc	Endothelial cells	T cells;Monocytic lineage;Neutrophils;Endothelial cells;Fibroblasts
TGM2	ITGB3	0.682706766917293	4	R-HSA-202733|R-HSA-354192|R-HSA-567413	Cell surface interactions at the vascular wall|Integrin alphaIIb beta3 si	T cells;Cytotoxic lymphocytes;NK cells;Mon	Monocytic lineage;Endothelial cells;Fibroblasts
TGM2	SDC4	0.553383458646617	4	R-HSA-202733|R-HSA-3781865	Cell surface interactions at the vascular wall|Diseases of glycosylatior	T cells;Cytotoxic lymphocytes;NK cells;Mon	Monocytic lineage;Fibroblasts
THBS1	CD36	0.675187969924812	4	R-HSA-166016|R-HSA-168898|R-HSA-198978	Toll Like Receptor 4 (TLR4) Cascade|Toll-Like Receptors Cascades|I	Fibroblasts	Fibroblasts
THBS1	ITGB1	0.604511278195489	4	R-HSA-6785807	Interleukin-4 and 13 signaling	Fibroblasts	Endothelial cells
THBS1	ITGB3	0.62406015037594	4	R-HSA-202733|R-HSA-354192|R-HSA-567413	Cell surface interactions at the vascular wall|Integrin alphaIIb beta3 si	Fibroblasts	Monocytic lineage;Endothelial cells;Fibroblasts
THBS1	SDC4	0.616541353383459	4	R-HSA-202733|R-HSA-3781865	Cell surface interactions at the vascular wall|Diseases of glycosylatior	Fibroblasts	Monocytic lineage;Fibroblasts
THBS2	ITGA6	0.526315789473684	4	R-HSA-1474290|R-HSA-1500931|R-HSA-2022	Collagen formation|Cell-Cell communication|Assembly of collagen fi	Endothelial cells	Neutrophils;Endothelial cells
THBS2	NOTCH3	0.732330827067669	4	R-HSA-157118|R-HSA-3781865|R-HSA-90128	Signaling by NOTCH|Diseases of glycosylation|Signaling by NOTCH	Endothelial cells	Endothelial cells
THBS2	NOTCH4	0.619548872180451	4	R-HSA-3781865	Diseases of glycosylation	Endothelial cells	Endothelial cells
TLN1	ITGB3	0.592481203007519	4	R-HSA-202733|R-HSA-354192|R-HSA-567413	Cell surface interactions at the vascular wall|Integrin alphaIIb beta3 si	T cells;Monocytic lineage;Myeloid dendritic	Monocytic lineage;Endothelial cells;Fibroblasts
TNC	ITGA5	0.732330827067669	4	R-HSA-1566977|R-HSA-202733	Fibronectin matrix formation|Cell surface interactions at the vascular	T cells;Monocytic lineage;Myeloid dendritic	NK cells;Monocytic lineage;Endothelial cells;Fibroblasts
TNC	ITGB3	0.633082706766917	4	R-HSA-202733|R-HSA-354192|R-HSA-567413	Cell surface interactions at the vascular wall|Integrin alphaIIb beta3 si	T cells;Monocytic lineage;Myeloid dendritic	Monocytic lineage;Endothelial cells;Fibroblasts
TNC	SDC4	0.595488721804511	4	R-HSA-202733|R-HSA-3781865	Cell surface interactions at the vascular wall|Diseases of glycosylatior	T cells;Monocytic lineage;Myeloid dendritic	Monocytic lineage;Fibroblasts
TNFSF13	SDC2	0.64812030075188	4	R-HSA-1630316|R-HSA-1638091|R-HSA-1793	Glycosaminoglycan metabolism|Heparan sulfate/heparin (HS-GAG) r	T cells;Monocytic lineage;Myeloid dendritic	Neutrophils;Endothelial cells
TNFSF13	TNFRSF1A	0.833082706766917	4	R-HSA-5668541|R-HSA-6783783|R-HSA-7388	TNFR2 non-canonical NF-kB pathway|Interleukin-10 signaling|Death	T cells;Monocytic lineage;Myeloid dendritic	T cells;Monocytic lineage;Neutrophils;Endothelial cells
TNFSF14	TNFRSF14	0.613533834586466	4	R-HSA-388841|R-HSA-5668541	Costimulation by the CD28 family|TNFR2 non-canonical NF-kB patl	T cells;Cytotoxic lymphocytes;Monocytic line	T cells;Cytotoxic lymphocytes;NK cells;Monocytic lineage;Myeloid dendritic c
TNFSF4	TRAF2	0.580451127819549	4	R-HSA-109581|R-HSA-140534|R-HSA-168928	Apoptosis|Ligand-dependent caspase activation|DDX58/IFIH1-media	T cells;Cytotoxic lymphocytes;Monocytic line	B lineage
TNFSF9	TNFRSF9	0.730827067669173	4	R-HSA-5668541	TNFR2 non-canonical NF-kB pathway	B lineage	B lineage;Monocytic lineage;Myeloid dendritic cells;Fibroblasts
TNFSF9	TRAF2	0.53984962406015	4	R-HSA-109581|R-HSA-140534|R-HSA-168928	Apoptosis|Ligand-dependent caspase activation|DDX58/IFIH1-media	B lineage	B lineage
UBC	RIPK1	0.753383458646616	4	R-HSA-109581|R-HSA-1606322|R-HSA-16601	Apoptosis|ZBP1(DAI) mediated induction of type I IFNs|Toll Like Re	T cells;Cytotoxic lymphocytes	T cells;Cytotoxic lymphocytes;NK cells;Monocytic lineage;Myeloid dendritic c
VCAM1	ITGA4	0.724812030075188	4	R-HSA-8878159	Transcriptional regulation by RUNX3	T cells;Cytotoxic lymphocytes;Monocytic line	T cells;Cytotoxic lymphocytes
VCAM1	ITGB2	0.590977443609023	4	R-HSA-166016|R-HSA-168898|R-HSA-202733	Toll Like Receptor 4 (TLR4) Cascade|Toll-Like Receptors Cascades|C	T cells;Cytotoxic lymphocytes;Monocytic line	T cells;NK cells;Monocytic lineage;Myeloid dendritic cells;Neutrophils

VCAN	EGFR	0.557894736842105	4	R-HSA-1227986|R-HSA-1236394|R-HSA-1250	Signaling by ERBB2|Signaling by ERBB4|SHC1 events in ERBB2 si	Endothelial cells	Neutrophils;Endothelial cells
VCAN	ITGB1	0.590977443609023	4	R-HSA-6785807	Interleukin-4 and 13 signaling	Endothelial cells	Endothelial cells
VEGFA	ITGB3	0.562406015037594	4	R-HSA-202733|R-HSA-354192|R-HSA-567413	Cell surface interactions at the vascular wall|Integrin alphaIIb beta3 si	Endothelial cells;Fibroblasts	Monocytic lineage;Endothelial cells;Fibroblasts
VEGFB	FLT1	0.592481203007519	4	R-HSA-194138	Signaling by VEGF	Neutrophils;Endothelial cells	Endothelial cells
VEGFB	NRP1	0.550375939849624	4	R-HSA-194138|R-HSA-373755|R-HSA-373760	Signaling by VEGF|Semaphorin interactions|L1CAM interactions	Neutrophils;Endothelial cells	Endothelial cells
VEGFC	FLT1	0.523308270676692	4	R-HSA-194138	Signaling by VEGF	Monocytic lineage;Endothelial cells;Fibroblas	Endothelial cells
VIM	CD44	0.748872180451128	4	R-HSA-1474228|R-HSA-202733	Degradation of the extracellular matrix|Cell surface interactions at the	Monocytic lineage;Myeloid dendritic cells;Fi	Monocytic lineage;Fibroblasts
VIP	ADCYAP1R1	0.617564448659594	4	R-HSA-166520|R-HSA-187037|R-HSA-373080	Signaling by NTRKs|Signaling by NTRK1 (TRKA)|Class B/2 (Secret	Neutrophils	Neutrophils;Endothelial cells
VIP	PTH1R	0.553089166598278	4	R-HSA-373080	Class B/2 (Secretin family receptors)	Neutrophils	Neutrophils;Endothelial cells
VIP	VIPR2	0.659831288733794	4	R-HSA-373080|R-HSA-420092	Class B/2 (Secretin family receptors)|Glucagon-type ligand receptors	Neutrophils	NA
VTN	ITGA2B	0.62279056679576	4	R-HSA-6802952|R-HSA-6802957	Signaling by BRAF and RAF fusions|Oncogenic MAPK signaling	Endothelial cells	Endothelial cells
VTN	ITGB5	0.602482178748072	4	R-HSA-445355	Smooth Muscle Contraction	Endothelial cells	Monocytic lineage;Endothelial cells
WNT5A	FZD1	0.702255639097744	4	R-HSA-373080|R-HSA-3858494|R-HSA-40864	Class B/2 (Secretin family receptors)|Beta-catenin independent WNT	Endothelial cells	T cells;Endothelial cells
WNT5A	FZD8	0.733834586466166	4	R-HSA-373080|R-HSA-3858494|R-HSA-40864	Class B/2 (Secretin family receptors)|Beta-catenin independent WNT	Endothelial cells	Neutrophils;Endothelial cells
WNT5A	RYK	0.628571428571429	4	R-HSA-3858494	Beta-catenin independent WNT signaling	Endothelial cells	NA
WNT7A	FZD10	0.681250868722914	4	R-HSA-373080	Class B/2 (Secretin family receptors)	Monocytic lineage;Endothelial cells	Neutrophils;Endothelial cells
WNT7A	FZD5	0.549499521469898	4	R-HSA-373080|R-HSA-3858494|R-HSA-40864	Class B/2 (Secretin family receptors)|Beta-catenin independent WNT	Monocytic lineage;Endothelial cells	Monocytic lineage
WNT7A	FZD9	0.640520255542905	4	R-HSA-373080	Class B/2 (Secretin family receptors)	Monocytic lineage;Endothelial cells	Neutrophils;Endothelial cells
VCAM1	MSN	0.560902255639098	4	R-HSA-373760|R-HSA-447115|R-HSA-895050	L1CAM interactions|Interleukin-12 family signaling|Gene and protein	T cells;Cytotoxic lymphocytes;Monocytic lin	T cells;Monocytic lineage;Myeloid dendritic cells;Fibroblasts
ADAM10	NOTCH1	0.645112781954887	4	R-HSA-157118|R-HSA-1980143|R-HSA-26446	Signaling by NOTCH|Signaling by NOTCH1|Signaling by NOTCH1	Endothelial cells	Endothelial cells
FADD	FAS	0.675187969924812	4	R-HSA-109581|R-HSA-140534|R-HSA-337137	Apoptosis|Ligand-dependent caspase activation|Regulation by c-FLIP	T cells;Cytotoxic lymphocytes;Monocytic lin	T cells;Cytotoxic lymphocytes;B lineage;NK cells;Monocytic lineage;Myeloid
ST6GAL1	CD22	0.571428571428571	4	R-HSA-5690714|R-HSA-983695|R-HSA-98370	CD22 mediated BCR regulation|Antigen activates B Cell Receptor (E	NA	NA
DLL4	NOTCH3	0.75187969924812	4	R-HSA-157118|R-HSA-3781865|R-HSA-90128	Signaling by NOTCH|Diseases of glycosylation|Signaling by NOTCH	Endothelial cells	Endothelial cells
ICAM1	SPN	0.804511278195489	4	R-HSA-202733	Cell surface interactions at the vascular wall	T cells;Cytotoxic lymphocytes;NK cells;Mon	T cells;Cytotoxic lymphocytes;Monocytic lineage
WNT5A	FZD3	0.584962406015038	4	R-HSA-3858494	Beta-catenin independent WNT signaling	Endothelial cells	Endothelial cells
CD274	CD80	0.584962406015038	4	R-HSA-199418|R-HSA-2219528|R-HSA-38884	Negative regulation of the PI3K/AKT network|PI3K/AKT Signaling i	T cells;Cytotoxic lymphocytes;NK cells;Mon	T cells;Cytotoxic lymphocytes;B lineage;NK cells
HLA-F	LILRB2	0.849624060150376	4	R-HSA-198933	Immunoregulatory interactions between a Lymphoid and a non-Lymp	T cells;Cytotoxic lymphocytes;NK cells;Mon	T cells;Cytotoxic lymphocytes;NK cells;Monocytic lineage
WNT2B	FZD4	0.538345864661654	4	R-HSA-3858494|R-HSA-8856825|R-HSA-8856	Beta-catenin independent WNT signaling|Cargo recognition for clath	Endothelial cells	Endothelial cells
ANXA1	FPR2	0.56390977443609	4	GO:0001934|GO:0006898	positive regulation of protein phosphorylation|receptor-mediated endo	T cells;Endothelial cells	T cells;Monocytic lineage;Neutrophils
B2M	CD247	0.78796992481203	4	GO:0002250|GO:0031295|GO:0050852	adaptive immune response|T cell costimulation|T cell receptor signali	T cells;Cytotoxic lymphocytes;NK cells;Mon	T cells;Cytotoxic lymphocytes;NK cells;Monocytic lineage
B2M	LILRB1	0.774436090225564	4	GO:0002250	adaptive immune response	T cells;Cytotoxic lymphocytes;NK cells;Mon	T cells;Myeloid dendritic cells
BTC	ERBB3	0.513554799266823	4	GO:0007169|GO:0009968|GO:0014068|GO:003	transmembrane receptor protein tyrosine kinase signaling pathway|ne	NA	Neutrophils;Endothelial cells
CNTF	CNTFR	0.586686765822093	4	GO:0043524	negative regulation of neuron apoptotic process	T cells;Neutrophils	Neutrophils;Endothelial cells
CNTF	IL6ST	0.586466165413534	4	GO:0042531	positive regulation of tyrosine phosphorylation of STAT protein	T cells;Neutrophils	Monocytic lineage;Endothelial cells
COL9A1	MAG	0.780121366628195	4	GO:0043524	negative regulation of neuron apoptotic process	Neutrophils;Endothelial cells	T cells;Neutrophils;Endothelial cells
COL9A2	MAG	0.586466165413534	4	GO:0043524	negative regulation of neuron apoptotic process	Endothelial cells	T cells;Neutrophils;Endothelial cells
COL9A3	MAG	0.666165413533835	4	GO:0043524	negative regulation of neuron apoptotic process	Endothelial cells	T cells;Neutrophils;Endothelial cells
CRH	CRHR2	0.587428571428571	4	GO:0007188	adenylate cyclase-modulating G-protein coupled receptor signaling pa	Endothelial cells	NA
EGF	ERBB3	0.6867477667615	4	GO:0007169|GO:0009968|GO:0014068|GO:003	transmembrane receptor protein tyrosine kinase signaling pathway|ne	Neutrophils;Endothelial cells	Neutrophils;Endothelial cells
EGF	ERBB4	0.719880334456133	4	GO:0001934|GO:0007169|GO:0014068|GO:003	positive regulation of protein phosphorylation|transmembrane recepto	Neutrophils;Endothelial cells	Neutrophils;Endothelial cells
FN1	ITGB7	0.518796992481203	4	GO:0007229	integrin-mediated signaling pathway	Fibroblasts	T cells;Cytotoxic lymphocytes;NK cells;Monocytic lineage
HLA-A	LILRB1	0.657142857142857	4	GO:0002250	adaptive immune response	T cells;Cytotoxic lymphocytes;NK cells;Mon	T cells;Myeloid dendritic cells
HLA-B	LILRB1	0.538345864661654	4	GO:0002250	adaptive immune response	T cells;Cytotoxic lymphocytes;Monocytic lin	T cells;Myeloid dendritic cells
HLA-C	LILRB1	0.532330827067669	4	GO:0002250	adaptive immune response	T cells;Cytotoxic lymphocytes;Monocytic lin	T cells;Myeloid dendritic cells
ICAM3	ITGAD	0.574436090225564	4	GO:0007229	integrin-mediated signaling pathway	B lineage	NA
IL18	IL1RL2	0.753051912956786	4	GO:0070498	interleukin-1-mediated signaling pathway	T cells;Cytotoxic lymphocytes;Monocytic lin	NA
LAMA1	ITGB8	0.726315789473684	4	GO:0007229	integrin-mediated signaling pathway	Neutrophils;Endothelial cells	Neutrophils;Endothelial cells
LTB	CD40	0.742857142857143	4	GO:0033209|GO:0043123	tumor necrosis factor-mediated signaling pathway|positive regulation	B lineage;Fibroblasts	B lineage;NK cells;Monocytic lineage;Fibroblasts
NRG3	ERBB3	0.700263306385088	4	GO:0007169|GO:0009968|GO:0014068|GO:003	transmembrane receptor protein tyrosine kinase signaling pathway|ne	Neutrophils;Endothelial cells	Neutrophils;Endothelial cells
NRG3	ERBB4	0.686724381019963	4	GO:0001934|GO:0007169|GO:0014068|GO:003	positive regulation of protein phosphorylation|transmembrane recepto	Neutrophils;Endothelial cells	Neutrophils;Endothelial cells
PLAU	PLAUR	0.709774436090226	4	GO:0007596	blood coagulation	T cells;Monocytic lineage;Myeloid dendritic	T cells;Cytotoxic lymphocytes;NK cells;Monocytic lineage;Neutrophils
PTN	CDH10	0.965179212381233	4	GO:0098609	cell-cell adhesion	T cells;Neutrophils;Endothelial cells	Neutrophils;Endothelial cells
RELN	VLDLR	0.691237356141671	4	GO:0006898	receptor-mediated endocytosis	Neutrophils;Endothelial cells	Neutrophils;Endothelial cells
SERPING1	SELE	0.538345864661654	4	GO:0050900	leukocyte migration	T cells;Cytotoxic lymphocytes;NK cells;Mon	T cells;Cytotoxic lymphocytes
SLIT2	ROBO1	0.639097744360902	4	GO:0002042	cell migration involved in sprouting angiogenesis	Endothelial cells	Endothelial cells
TGFA	ERBB3	0.825563909774436	4	GO:0007169|GO:0009968|GO:0014068|GO:003	transmembrane receptor protein tyrosine kinase signaling pathway|ne	Neutrophils;Endothelial cells	Neutrophils;Endothelial cells
TGFA	ERBB4	0.849624060150376	4	GO:0001934|GO:0007169|GO:0014068|GO:003	positive regulation of protein phosphorylation|transmembrane recepto	Neutrophils;Endothelial cells	Neutrophils;Endothelial cells
VCAM1	ITGAD	0.565413533834586	4	GO:0007229	integrin-mediated signaling pathway	T cells;Cytotoxic lymphocytes;Monocytic lin	NA
VCAM1	ITGB7	0.627067669172932	4	GO:0007229	integrin-mediated signaling pathway	T cells;Cytotoxic lymphocytes;Monocytic lin	T cells;Cytotoxic lymphocytes;NK cells;Monocytic lineage
HLA-F	LILRB1	0.741353383458647	4	GO:0002250	adaptive immune response	T cells;Cytotoxic lymphocytes;NK cells;Mon	T cells;Myeloid dendritic cells

cells

cells

dendritic cells;Fibroblasts

cells

cells

cells

cells

cells;Fibroblasts

cells

:ells;Fibroblasts

:ells

dendritic cells;Fibroblasts

Table S7 L-R pairs selected by microarrays analysis (n=34)

Ligand	Receptor	corr	num.cor.pw	pwid	pwname	L.ct	R.ct
217757_at A2M	200785_s_at LRP1	0.575859434682964	4	R-HSA-196854|R-HSA-2187338|R-HSA-	Metabolism of vitamins and cofactors|Visual phototransduction|M	Monocytic lineage;Neutrophils;Endothelial cells	NA
234212_at ACTR2	217103_at LDLR	0.554774637127578	4	R-HSA-174824|R-HSA-196854|R-HSA-2	Plasma lipoprotein assembly, remodeling, and clearance|Metaboli	T cells;Neutrophils;Endothelial cells	T cells;B lineage;NK cells;Myeloid dendritic cells;Neutrophi
202603_at ADAM10	202686_s_at AXL	0.528800611153552	4	R-HSA-194138|R-HSA-4420097	Signaling by VEGF|VEGFA-VEGFR2 Pathway	NA	T cells;Monocytic lineage;Endothelial cells
215613_at ADAM12	216190_x_at ITGB1	0.557219251336898	4	R-HSA-1500931|R-HSA-202733|R-HSA-	Cell-Cell communication|Cell surface interactions at the vascular	Neutrophils;Endothelial cells	T cells;Neutrophils;Endothelial cells;Fibroblasts
1555896_a_at ADAM15	206009_at ITGA9	0.765011459129106	4	R-HSA-373760	L1CAM interactions	T cells;B lineage;NK cells;Myeloid dendritic cells;Neutr	T cells;B lineage;NK cells;Neutrophils;Endothelial cells
1555896_a_at ADAM15	215878_at ITGB1	0.679807473906995	4	R-HSA-202733|R-HSA-373755|R-HSA-3	Cell surface interactions at the vascular wall|Semaphorin interacti	T cells;B lineage;NK cells;Myeloid dendritic cells;Neutr	T cells
1555896_a_at ADAM15	211579_at ITGB3	0.60722744466316	4	R-HSA-194138|R-HSA-202733|R-HSA-2	Signaling by VEGF|Cell surface interactions at the vascular wall|I	T cells;B lineage;NK cells;Myeloid dendritic cells;Neutr	T cells;B lineage;NK cells;Myeloid dendritic cells;Neutrophi
213532_at ADAM17	211945_s_at ITGB1	0.596027501909855	4	R-HSA-1500931|R-HSA-202733|R-HSA-	Cell-Cell communication|Cell surface interactions at the vascular	NA	NA
1559268_at ADAM23	204625_s_at ITGB3	0.584110007639419	4	R-HSA-194138|R-HSA-202733|R-HSA-3	Signaling by VEGF|Cell surface interactions at the vascular wall|I	T cells;B lineage;Myeloid dendritic cells;Neutrophils;En	T cells;B lineage;Neutrophils;Fibroblasts
207664_at ADAM2	229643_at ITGA6	0.503132161955691	4	R-HSA-1474290|R-HSA-2022090|R-HSA	Collagen formation|Assembly of collagen fibrils and other multim	T cells;B lineage;Myeloid dendritic cells;Neutrophils;En	Fibroblasts
207664_at ADAM2	1555336_a_at ITGA9	0.618334606569901	4	R-HSA-373760	L1CAM interactions	T cells;B lineage;Myeloid dendritic cells;Neutrophils;En	CD8 T cells;Endothelial cells;Fibroblasts
207664_at ADAM2	215879_at ITGB1	0.608861726508785	4	R-HSA-1500931|R-HSA-202733|R-HSA-	Cell-Cell communication|Cell surface interactions at the vascular	T cells;B lineage;Myeloid dendritic cells;Neutrophils;En	NK cells;Neutrophils;Endothelial cells
202381_at ADAM9	201656_at ITGA6	0.707257448433919	4	R-HSA-1474290|R-HSA-1500931|R-HSA	Collagen formation|Cell-Cell communication|Assembly of collage	Neutrophils	Neutrophils;Endothelial cells
202381_at ADAM9	202351_at ITGAV	0.825515660809778	4	R-HSA-194138|R-HSA-202733|R-HSA-2	Signaling by VEGF|Cell surface interactions at the vascular wall|I	Neutrophils	Neutrophils;Endothelial cells
202381_at ADAM9	211945_s_at ITGB1	0.811459129106188	4	R-HSA-1500931|R-HSA-202733|R-HSA-	Cell-Cell communication|Cell surface interactions at the vascular	Neutrophils	NA
206281_at ADCYAP1	210382_at SCTR	0.523187410801877	4	R-HSA-373080	Class B/2 (Secretin family receptors)	Neutrophils;Endothelial cells	NA
206281_at ADCYAP1	205019_s_at VIPR1	0.712146676852559	4	R-HSA-373080|R-HSA-420092	Class B/2 (Secretin family receptors)|Glucagon-type ligand recept	Neutrophils;Endothelial cells	NA
220538_at ADM2	207151_at ADCYAP1R1	0.524828113063407	4	R-HSA-166520|R-HSA-187037|R-HSA-3	Signaling by NTRKs|Signaling by NTRK1 (TRKA)|Class B/2 (Se	T cells;Myeloid dendritic cells;Neutrophils;Endothelial c	T cells
220538_at ADM2	207887_s_at CALCR	0.662032085561497	4	R-HSA-373080	Class B/2 (Secretin family receptors)	T cells;Myeloid dendritic cells;Neutrophils;Endothelial c	T cells;B lineage;Myeloid dendritic cells;Neutrophils;Endoth
217410_at AGRN	214432_at ATP1A3	0.723355491453296	4	R-HSA-5576891|R-HSA-5578775|R-HSA	Cardiac conduction|Ion homeostasis|Ion transport by P-type ATPa	T cells;B lineage;Myeloid dendritic cells;Neutrophils;En	B lineage;Myeloid dendritic cells;Neutrophils;Endothelial ce
217410_at AGRN	215878_at ITGB1	0.737565897178931	4	R-HSA-202733|R-HSA-373755|R-HSA-3	Cell surface interactions at the vascular wall|Semaphorin interacti	T cells;B lineage;Myeloid dendritic cells;Neutrophils;En	T cells
217410_at AGRN	1569042_at LRP1	0.721313980137509	4	R-HSA-196854|R-HSA-2168880|R-HSA-	Metabolism of vitamins and cofactors|Scavenging of heme from p	T cells;B lineage;Myeloid dendritic cells;Neutrophils;En	T cells;B lineage;Myeloid dendritic cells;Neutrophils;Endoth
202834_at AGT	230863_at LRP2	0.703590527119939	4	R-HSA-196854|R-HSA-2187338|R-HSA-	Metabolism of vitamins and cofactors|Visual phototransduction|M	Neutrophils;Endothelial cells;Fibroblasts	Neutrophils;Endothelial cells;Fibroblasts
210929_s_at AHSG	226212_s_at INSR	0.566997708174179	4	R-HSA-199418|R-HSA-6811558|R-HSA-	Negative regulation of the PI3K/AKT network|PI5P, PP2A and IE	T cells;Myeloid dendritic cells;Neutrophils;Endothelial c	T cells;Endothelial cells
1552939_at ANGPT1	215879_at ITGB1	0.667532467532468	4	R-HSA-1500931|R-HSA-202733|R-HSA-	Cell-Cell communication|Cell surface interactions at the vascular	T cells;Neutrophils;Endothelial cells;Fibroblasts	NK cells;Neutrophils;Endothelial cells
1552939_at ANGPT1	217711_at TEK	0.608250572956455	4	R-HSA-202733	Cell surface interactions at the vascular wall	T cells;Neutrophils;Endothelial cells;Fibroblasts	T cells;CD8 T cells;B lineage;NK cells;Myeloid dendritic ce
239183_at ANGPTL1	217711_at TEK	0.621084797555386	4	R-HSA-202733	Cell surface interactions at the vascular wall	T cells;B lineage;Myeloid dendritic cells;Neutrophils;En	T cells;CD8 T cells;B lineage;NK cells;Myeloid dendritic ce
219803_at ANGPTL3	211579_at ITGB3	0.721216290591372	4	R-HSA-194138|R-HSA-202733|R-HSA-2	Signaling by VEGF|Cell surface interactions at the vascular wall|I	T cells;B lineage;NK cells;Myeloid dendritic cells;Neutr	T cells;B lineage;NK cells;Myeloid dendritic cells;Neutrophi
205206_at ANOS1	212158_at SDC2	0.646753246753247	4	R-HSA-1630316|R-HSA-1638091|R-HSA	Glycosaminoglycan metabolism|Heparan sulfate/heparin (HS-GA	Monocytic lineage;Neutrophils;Endothelial cells;Fibrobl	Endothelial cells
201012_at ANXA1	205119_s_at FPR1	0.644308632543927	4	R-HSA-6783783	Interleukin-10 signaling	Monocytic lineage	NA
231694_at APOA1	217103_at LDLR	0.640641711229947	4	R-HSA-174824|R-HSA-196854|R-HSA-2	Plasma lipoprotein assembly, remodeling, and clearance|Metaboli	T cells;Myeloid dendritic cells;Neutrophils;Endothelial c	T cells;B lineage;NK cells;Myeloid dendritic cells;Neutrophi
231694_at APOA1	1569042_at LRP1	0.686478227654698	4	R-HSA-196854|R-HSA-2168880|R-HSA-	Metabolism of vitamins and cofactors|Scavenging of heme from p	T cells;Myeloid dendritic cells;Neutrophils;Endothelial c	T cells;B lineage;Myeloid dendritic cells;Neutrophils;Endoth
219465_at APOA2	217005_at LDLR	0.530939648586707	4	R-HSA-174824|R-HSA-196854|R-HSA-2	Plasma lipoprotein assembly, remodeling, and clearance|Metaboli	NA	B lineage;Neutrophils;Endothelial cells
219465_at APOA2	1569042_at LRP1	0.700534759358289	4	R-HSA-196854|R-HSA-2168880|R-HSA-	Metabolism of vitamins and cofactors|Scavenging of heme from p	NA	T cells;B lineage;Myeloid dendritic cells;Neutrophils;Endoth
206894_at APOA4	217005_at LDLR	0.58716577540107	4	R-HSA-174824|R-HSA-196854|R-HSA-2	Plasma lipoprotein assembly, remodeling, and clearance|Metaboli	B lineage;Endothelial cells;Fibroblasts	B lineage;Neutrophils;Endothelial cells
206894_at APOA4	1569042_at LRP1	0.563025210084034	4	R-HSA-196854|R-HSA-2168880|R-HSA-	Metabolism of vitamins and cofactors|Scavenging of heme from p	B lineage;Endothelial cells;Fibroblasts	T cells;B lineage;Myeloid dendritic cells;Neutrophils;Endoth
223579_s_at APOB	207887_s_at CALCR	0.733745895639782	4	R-HSA-373080	Class B/2 (Secretin family receptors)	T cells;B lineage;Neutrophils;Endothelial cells;Fibroblas	T cells;B lineage;Myeloid dendritic cells;Neutrophils;Endoth
223579_s_at APOB	217005_at LDLR	0.605241043862802	4	R-HSA-174824|R-HSA-196854|R-HSA-2	Plasma lipoprotein assembly, remodeling, and clearance|Metaboli	T cells;B lineage;Neutrophils;Endothelial cells;Fibroblas	B lineage;Neutrophils;Endothelial cells
223579_s_at APOB	1555353_at LRP1	0.758939486552567	4	R-HSA-196854|R-HSA-2168880|R-HSA-	Metabolism of vitamins and cofactors|Scavenging of heme from p	T cells;B lineage;Neutrophils;Endothelial cells;Fibroblas	B lineage;NK cells;Neutrophils;Endothelial cells;Fibroblasts
223579_s_at APOB	205606_at LRP6	0.563985027239991	4	R-HSA-4641262|R-HSA-4791275	Disassembly of the destruction complex and recruitment of AXIN	T cells;B lineage;Neutrophils;Endothelial cells;Fibroblas	T cells;Myeloid dendritic cells;Neutrophils;Endothelial cells
223579_s_at APOB	205675_at MTTP	0.526894865525672	4	R-HSA-174824	Plasma lipoprotein assembly, remodeling, and clearance	T cells;B lineage;Neutrophils;Endothelial cells;Fibroblas	T cells;Neutrophils;Endothelial cells
231561_s_at APOC2	217005_at LDLR	0.658976317799847	4	R-HSA-174824|R-HSA-196854|R-HSA-2	Plasma lipoprotein assembly, remodeling, and clearance|Metaboli	Endothelial cells	B lineage;Neutrophils;Endothelial cells
231561_s_at APOC2	200784_s_at LRP1	0.573414820473644	4	R-HSA-196854|R-HSA-2187338|R-HSA-	Metabolism of vitamins and cofactors|Visual phototransduction|M	Endothelial cells	T cells;Endothelial cells
205820_s_at APOC3	217005_at LDLR	0.677005347593583	4	R-HSA-174824|R-HSA-196854|R-HSA-2	Plasma lipoprotein assembly, remodeling, and clearance|Metaboli	T cells;B lineage;NK cells;Myeloid dendritic cells;Neutr	B lineage;Neutrophils;Endothelial cells
205820_s_at APOC3	1569042_at LRP1	0.705423987776929	4	R-HSA-196854|R-HSA-2168880|R-HSA-	Metabolism of vitamins and cofactors|Scavenging of heme from p	T cells;B lineage;NK cells;Myeloid dendritic cells;Neutr	T cells;B lineage;Myeloid dendritic cells;Neutrophils;Endoth
212874_at APOE	217005_at LDLR	0.525744843391902	4	R-HSA-174824|R-HSA-196854|R-HSA-2	Plasma lipoprotein assembly, remodeling, and clearance|Metaboli	NA	B lineage;Neutrophils;Endothelial cells
212884_x_at APOE	200785_s_at LRP1	0.73659281894576	4	R-HSA-196854|R-HSA-2187338|R-HSA-	Metabolism of vitamins and cofactors|Visual phototransduction|M	Monocytic lineage;Neutrophils	NA
214953_s_at APP	202756_s_at GPC1	0.666615737203972	4	R-HSA-1630316|R-HSA-1638091|R-HSA	Glycosaminoglycan metabolism|Heparan sulfate/heparin (HS-GA	Neutrophils;Endothelial cells	Endothelial cells
214953_s_at APP	200785_s_at LRP1	0.646753246753247	4	R-HSA-196854|R-HSA-2187338|R-HSA-	Metabolism of vitamins and cofactors|Visual phototransduction|M	Neutrophils;Endothelial cells	NA
215564_at AREG	211607_x_at EGFR	0.790068754774637	4	R-HSA-1227986|R-HSA-1236382|R-HSA	Signaling by ERBB2|Constitutive Signaling by Ligand-Responsiv	T cells;B lineage;Myeloid dendritic cells;Neutrophils;En	T cells;B lineage;NK cells;Myeloid dendritic cells;Neutrophi
1569325_at ARPC5	217103_at LDLR	0.582582123758594	4	R-HSA-174824|R-HSA-196854|R-HSA-2	Plasma lipoprotein assembly, remodeling, and clearance|Metaboli	Neutrophils;Endothelial cells;Fibroblasts	T cells;B lineage;NK cells;Myeloid dendritic cells;Neutrophi
210237_at ARTN	205696_s_at GFRA1	0.556913674560733	4	R-HSA-8853659	RET signaling	T cells;B lineage;NK cells;Neutrophils;Endothelial cells;	NA
210237_at ARTN	214479_at GFRA3	0.633307868601986	4	R-HSA-8853659	RET signaling	T cells;B lineage;NK cells;Neutrophils;Endothelial cells;	Endothelial cells
210237_at ARTN	215771_x_at RET	0.690145148968678	4	R-HSA-8853659	RET signaling	T cells;B lineage;NK cells;Neutrophils;Endothelial cells;	T cells;B lineage;Myeloid dendritic cells;Neutrophils;Endoth
214498_at ASIP	222354_at F11R	0.528800611153552	4	R-HSA-170834|R-HSA-202733|R-HSA-9	Signaling by TGF-beta Receptor Complex|Cell surface interaction	Neutrophils;Endothelial cells	Neutrophils
207848_at AVP	207151_at ADCYAP1R1	0.569442322383499	4	R-HSA-166520|R-HSA-187037|R-HSA-3	Signaling by NTRKs|Signaling by NTRK1 (TRKA)|Class B/2 (Se	T cells;B lineage;NK cells;Myeloid dendritic cells;Neutr	T cells
207848_at AVP	206251_s_at AVPR1A	0.7231474407945	4	R-HSA-5619115	Disorders of transmembrane transporters	T cells;B lineage;NK cells;Myeloid dendritic cells;Neutr	T cells;B lineage;Neutrophils;Endothelial cells;Fibroblasts
207848_at AVP	208111_at AVPR2	0.703590527119939	4	R-HSA-432040|R-HSA-445717|R-HSA-5	Vasopressin regulates renal water homeostasis via Aquaporins|Aq	T cells;B lineage;NK cells;Myeloid dendritic cells;Neutr	Endothelial cells;Fibroblasts
207848_at AVP	217103_at LDLR	0.752788388082506	4	R-HSA-174824|R-HSA-196854|R-HSA-2	Plasma lipoprotein assembly, remodeling, and clearance|Metaboli	T cells;B lineage;NK cells;Myeloid dendritic cells;Neutr	T cells;B lineage;NK cells;Myeloid dendritic cells;Neutrophi
207848_at AVP	205019_s_at VIPR1	0.596638655462185	4	R-HSA-373080|R-HSA-420092	Class B/2 (Secretin family receptors)|Glucagon-type ligand recept	T cells;B lineage;NK cells;Myeloid dendritic cells;Neutr	NA
216231_s_at B2M	221875_x_at HLA-F	0.762872421695951	4	R-HSA-1236977|R-HSA-877300|R-HSA-	Endosomal/Vacuolar pathway|Interferon gamma signaling|Antige	NA	NA
91920_at BCAN	210984_x_at EGFR	0.685255920550038	4	R-HSA-1227986|R-HSA-1236382|R-HSA	Signaling by ERBB2|Constitutive Signaling by Ligand-Responsiv	NA	T cells;B lineage;NK cells;Myeloid dendritic cells;Neutrophi
206382_s_at BDNF	229463_at NTRK2	0.513521772345302	4	R-HSA-166520|R-HSA-187037	Signaling by NTRKs|Signaling by NTRK1 (TRKA)	Endothelial cells	Endothelial cells
208292_at BMP10	210523_at BMPR1B	0.676394194041253	4	R-HSA-201451|R-HSA-9006936	Signaling by BMP|Signaling by TGF-beta family members	T cells;B lineage;Myeloid dendritic cells;Neutrophils;En	T cells;Myeloid dendritic cells;Neutrophils;Endothelial cells;
208292_at BMP10	1556075_at BMPR2	0.646753246753247	4	R-HSA-201451|R-HSA-9006936	Signaling by BMP|Signaling by TGF-beta family members	T cells;B lineage;Myeloid dendritic cells;Neutrophils;En	T cells;B lineage;Myeloid dendritic cells;Neutrophils;Endoth

221332_at BMP15	230979_at BMPR1A	0.557830404889228	4	R-HSA-201451|R-HSA-9006936	Signaling by BMP|Signaling by TGF-beta family members	T cells;NK cells;Neutrophils;Endothelial cells	B lineage;Neutrophils;Endothelial cells;Fibroblasts
221332_at BMP15	210523_at BMPR1B	0.618945760122231	4	R-HSA-201451|R-HSA-9006936	Signaling by BMP|Signaling by TGF-beta family members	T cells;NK cells;Neutrophils;Endothelial cells	T cells;Myeloid dendritic cells;Neutrophils;Endothelial cells;
221332_at BMP15	1556075_at BMPR2	0.617112299465241	4	R-HSA-201451|R-HSA-9006936	Signaling by BMP|Signaling by TGF-beta family members	T cells;NK cells;Neutrophils;Endothelial cells	T cells;B lineage;Myeloid dendritic cells;Neutrophils;Endoth
205289_at BMP2	1559548_at ACVR2B	0.535217723453018	4	R-HSA-1181150|R-HSA-1502540|R-HSA	Signaling by NODAL|Signaling by Activin|Signaling by BMP|Sig	Endothelial cells	B lineage;NK cells;Myeloid dendritic cells;Neutrophils;Endo
205289_at BMP2	210523_at BMPR1B	0.558441558441558	4	R-HSA-201451|R-HSA-9006936	Signaling by BMP|Signaling by TGF-beta family members	Endothelial cells	T cells;Myeloid dendritic cells;Neutrophils;Endothelial cells;
211518_s_at BMP4	230979_at BMPR1A	0.581359816653934	4	R-HSA-201451|R-HSA-9006936	Signaling by BMP|Signaling by TGF-beta family members	T cells;B lineage;Neutrophils;Endothelial cells;Fibroblas	B lineage;Neutrophils;Endothelial cells;Fibroblasts
211518_s_at BMP4	210523_at BMPR1B	0.530328495034377	4	R-HSA-201451|R-HSA-9006936	Signaling by BMP|Signaling by TGF-beta family members	T cells;B lineage;Neutrophils;Endothelial cells;Fibroblas	T cells;Myeloid dendritic cells;Neutrophils;Endothelial cells;
215042_at BMP6	230979_at BMPR1A	0.531245225362872	4	R-HSA-201451|R-HSA-9006936	Signaling by BMP|Signaling by TGF-beta family members	B lineage;Myeloid dendritic cells;Neutrophils;Endotheli	B lineage;Neutrophils;Endothelial cells;Fibroblasts
215042_at BMP6	210523_at BMPR1B	0.806569900687548	4	R-HSA-201451|R-HSA-9006936	Signaling by BMP|Signaling by TGF-beta family members	B lineage;Myeloid dendritic cells;Neutrophils;Endotheli	T cells;Myeloid dendritic cells;Neutrophils;Endothelial cells;
215042_at BMP6	1556075_at BMPR2	0.611306340718105	4	R-HSA-201451|R-HSA-9006936	Signaling by BMP|Signaling by TGF-beta family members	B lineage;Myeloid dendritic cells;Neutrophils;Endotheli	T cells;B lineage;Myeloid dendritic cells;Neutrophils;Endoth
211259_s_at BMP7	230979_at BMPR1A	0.678126673229769	4	R-HSA-201451|R-HSA-9006936	Signaling by BMP|Signaling by TGF-beta family members	B lineage;Neutrophils;Endothelial cells;Fibroblasts	B lineage;Neutrophils;Endothelial cells;Fibroblasts
211259_s_at BMP7	1556075_at BMPR2	0.648941861470669	4	R-HSA-201451|R-HSA-9006936	Signaling by BMP|Signaling by TGF-beta family members	B lineage;Neutrophils;Endothelial cells;Fibroblasts	T cells;B lineage;Myeloid dendritic cells;Neutrophils;Endoth
220204_s_at BMP8A	230979_at BMPR1A	0.512299465240642	4	R-HSA-201451|R-HSA-9006936	Signaling by BMP|Signaling by TGF-beta family members	T cells;B lineage;Neutrophils;Endothelial cells	B lineage;Neutrophils;Endothelial cells;Fibroblasts
220204_s_at BMP8A	210523_at BMPR1B	0.60977845683728	4	R-HSA-201451|R-HSA-9006936	Signaling by BMP|Signaling by TGF-beta family members	T cells;B lineage;Neutrophils;Endothelial cells	T cells;Myeloid dendritic cells;Neutrophils;Endothelial cells;
220204_s_at BMP8A	1556075_at BMPR2	0.580443086325439	4	R-HSA-201451|R-HSA-9006936	Signaling by BMP|Signaling by TGF-beta family members	T cells;B lineage;Neutrophils;Endothelial cells	T cells;B lineage;Myeloid dendritic cells;Neutrophils;Endoth
207865_s_at BMP8B	230979_at BMPR1A	0.628418640183346	4	R-HSA-201451|R-HSA-9006936	Signaling by BMP|Signaling by TGF-beta family members	T cells;B lineage;Myeloid dendritic cells;Neutrophils;En	B lineage;Neutrophils;Endothelial cells;Fibroblasts
207865_s_at BMP8B	1556075_at BMPR2	0.578915202444614	4	R-HSA-201451|R-HSA-9006936	Signaling by BMP|Signaling by TGF-beta family members	T cells;B lineage;Myeloid dendritic cells;Neutrophils;En	T cells;B lineage;Myeloid dendritic cells;Neutrophils;Endoth
207326_at BTC	211551_at EGFR	0.561845826378067	4	R-HSA-1227986|R-HSA-1236394|R-HSA	Signaling by ERBB2|Signaling by ERBB4|SHC1 events in ERBE	Endothelial cells	T cells;B lineage;Myeloid dendritic cells;Neutrophils;Endoth
236226_at BTLA	1555779_a_at CD79A	0.735676088617265	4	R-HSA-5690714|R-HSA-983695|R-HSA-	CD22 mediated BCR regulation|Antigen activates B Cell Recepto	B lineage	B lineage
202953_at C1QB	200785_s_at LRP1	0.661115355233002	4	R-HSA-196854|R-HSA-2187338|R-HSA-	Metabolism of vitamins and cofactors|Visual phototransduction|M	T cells;Monocytic lineage	NA
224197_s_at C1QTNF1	208111_at AVPR2	0.544385026737968	4	R-HSA-432040|R-HSA-445717|R-HSA-5	Vasopressin regulates renal water homeostasis via Aquaporins|Aq	NK cells	Endothelial cells;Fibroblasts
217767_at C3	209906_at C3AR1	0.717647058823529	4	R-HSA-166658	Complement cascade	Neutrophils;Endothelial cells;Fibroblasts	Monocytic lineage
217767_at C3	200675_at CD81	0.656531703590527	4	R-HSA-166658	Complement cascade	Neutrophils;Endothelial cells;Fibroblasts	Monocytic lineage;Neutrophils
217767_at C3	205786_s_at ITGAM	0.622612681436211	4	R-HSA-166016|R-HSA-168898|R-HSA-2	Toll Like Receptor 4 (TLR4) Cascade|Toll-Like Receptors Cascac	Neutrophils;Endothelial cells;Fibroblasts	Monocytic lineage
217767_at C3	200785_s_at LRP1	0.672421695951108	4	R-HSA-196854|R-HSA-2187338|R-HSA-	Metabolism of vitamins and cofactors|Visual phototransduction|M	Neutrophils;Endothelial cells;Fibroblasts	NA
210728_s_at CALCA	207151_at ADCYAP1R1	0.628418640183346	4	R-HSA-166520|R-HSA-187037|R-HSA-3	Signaling by NTRKs|Signaling by NTRK1 (TRKA)|Class B/2 (Se	NA	T cells
217561_at CALCA	207887_s_at CALCR	0.73109243697479	4	R-HSA-373080	Class B/2 (Secretin family receptors)	T cells;B lineage;NK cells;Myeloid dendritic cells;Neutr	T cells;B lineage;Myeloid dendritic cells;Neutrophils;Endoth
217561_at CALCA	206331_at CALCRL	0.542593018620755	4	R-HSA-373080	Class B/2 (Secretin family receptors)	T cells;B lineage;NK cells;Myeloid dendritic cells;Neutr	T cells;Myeloid dendritic cells;Neutrophils;Endothelial cells
213710_s_at CALM1	206811_at ADCY8	0.639621057715145	4	R-HSA-111885|R-HSA-111933|R-HSA-1	Opioid Signalling|Calmodulin induced events|Ca-dependent even	Endothelial cells	Neutrophils;Endothelial cells
213710_s_at CALM1	238636_at CACNA1C	0.513827349121467	4	R-HSA-163685|R-HSA-400042|R-HSA-4	Integration of energy metabolism|Adrenaline,noradrenaline inhibi	Endothelial cells	NA
213710_s_at CALM1	214619_at CRHR1	0.537051184110008	4	R-HSA-373080	Class B/2 (Secretin family receptors)	Endothelial cells	B lineage;NK cells;Myeloid dendritic cells;Neutrophils;Endo
213710_s_at CALM1	201984_s_at EGFR	0.757677616501146	4	R-HSA-1227986|R-HSA-1236382|R-HSA	Signaling by ERBB2|Constitutive Signaling by Ligand-Responsiv	Endothelial cells	T cells;NK cells;Neutrophils;Endothelial cells
213710_s_at CALM1	214217_at GRM5	0.554469060351413	4	R-HSA-6794362	Protein-protein interactions at synapses	Endothelial cells	NA
213710_s_at CALM1	207851_s_at INSR	0.509243697478992	4	R-HSA-199418|R-HSA-6811558|R-HSA-	Negative regulation of the PI3K/AKT network|PI5P, PP2A and IE	Endothelial cells	Neutrophils;Fibroblasts
213710_s_at CALM1	1557042_at KCNQ3	0.526355996944232	4	R-HSA-373760	L1CAM interactions	Endothelial cells	T cells;B lineage;NK cells;Myeloid dendritic cells;Neutrophi
213710_s_at CALM1	1568770_at MYLK	0.528495034377387	4	R-HSA-445355|R-HSA-5627123	Smooth Muscle Contraction|RHO GTPases activate PAKs	Endothelial cells	T cells;B lineage;NK cells;Myeloid dendritic cells;Neutrophi
213710_s_at CALM1	236234_at PDE1A	0.621695951107716	4	R-HSA-111885|R-HSA-112040|R-HSA-1	Opioid Signalling|G-protein mediated events|PLC beta mediated e	Endothelial cells	Endothelial cells
213710_s_at CALM1	243564_at PDE1C	0.646142093200917	4	R-HSA-111885|R-HSA-111933|R-HSA-1	Opioid Signalling|Calmodulin induced events|Ca-dependent even	Endothelial cells	NA
213710_s_at CALM1	205019_s_at VIPR1	0.572803666921314	4	R-HSA-373080|R-HSA-420092	Class B/2 (Secretin family receptors)|Glucagon-type ligand recept	Endothelial cells	NA
212953_x_at CALR	216956_s_at ITGA2B	0.581601473783277	4	R-HSA-114608|R-HSA-354192|R-HSA-3	Platelet degranulation|Integrin alphaIIb beta3 signaling|L1CAM ir	B lineage	T cells;B lineage;Myeloid dendritic cells;Neutrophils;Endoth
212953_x_at CALR	1555353_at LRP1	0.525021011540669	4	R-HSA-196854|R-HSA-2168880|R-HSA-	Metabolism of vitamins and cofactors|Scavenging of heme from p	B lineage	B lineage;NK cells;Neutrophils;Endothelial cells;Fibroblasts
207354_at CCL16	207794_at CCR2	0.537356760886173	4	R-HSA-6783783	Interleukin-10 signaling	T cells;B lineage;NK cells;Myeloid dendritic cells;Neutr	Endothelial cells
210072_at CCL19	209869_at ADRA2A	0.540106951871658	4	R-HSA-163685|R-HSA-422356	Integration of energy metabolism|Regulation of insulin secretion	NA	Fibroblasts
221463_at CCL24	207794_at CCR2	0.655920550038197	4	R-HSA-6783783	Interleukin-10 signaling	T cells;B lineage;Myeloid dendritic cells;Neutrophils;En	Endothelial cells
204103_at CCL4	205098_at CCR1	0.702979373567609	4	R-HSA-6783783	Interleukin-10 signaling	T cells;Cytotoxic lymphocytes	Monocytic lineage
204103_at CCL4	206991_s_at CCR5	0.726508785332315	4	R-HSA-162587|R-HSA-6783783	HIV Life Cycle|Interleukin-10 signaling	T cells;Cytotoxic lymphocytes	T cells;Cytotoxic lymphocytes;Monocytic lineage
1555759_a_at CCL5	205098_at CCR1	0.661420932009167	4	R-HSA-6783783	Interleukin-10 signaling	T cells;Cytotoxic lymphocytes	Monocytic lineage
1555759_a_at CCL5	206991_s_at CCR5	0.779984721161192	4	R-HSA-162587|R-HSA-6783783	HIV Life Cycle|Interleukin-10 signaling	T cells;Cytotoxic lymphocytes	T cells;Cytotoxic lymphocytes;Monocytic lineage
214038_at CCL8	205098_at CCR1	0.625668449197861	4	R-HSA-6783783	Interleukin-10 signaling	NA	Monocytic lineage
201743_at CD14	1555349_a_at ITGB2	0.731398013750955	4	R-HSA-166016|R-HSA-168898|R-HSA-2	Toll Like Receptor 4 (TLR4) Cascade|Toll-Like Receptors Cascac	Monocytic lineage;Neutrophils	Monocytic lineage
209554_at CD36	207446_at TLR6	0.629029793735676	4	R-HSA-166016|R-HSA-166058|R-HSA-1	Toll Like Receptor 4 (TLR4) Cascade|MyD88:Mal cascade initiat	T cells;B lineage;NK cells;Myeloid dendritic cells;Neutr	Endothelial cells
207892_at CD40LG	205785_at ITGAM	0.685561497326203	4	R-HSA-166016|R-HSA-168898|R-HSA-2	Toll Like Receptor 4 (TLR4) Cascade|Toll-Like Receptors Cascac	T cells;Myeloid dendritic cells;Neutrophils;Endothelial c	T cells;Neutrophils;Endothelial cells;Fibroblasts
207176_s_at CD80	234362_s_at CTLA4	0.555385790679908	4	R-HSA-388841|R-HSA-389513	Costimulation by the CD28 family|CTLA4 inhibitory signaling	Neutrophils	T cells;Endothelial cells
201130_s_at CDH1	211551_at EGFR	0.651642475171887	4	R-HSA-1227986|R-HSA-1236394|R-HSA	Signaling by ERBB2|Signaling by ERBB4|SHC1 events in ERBE	T cells;Myeloid dendritic cells;Neutrophils;Endothelial c	T cells;B lineage;Myeloid dendritic cells;Neutrophils;Endoth
201131_s_at CDH1	225330_at IGF1R	0.537051184110008	4	R-HSA-2404192|R-HSA-2428924|R-HSA	Signaling by Type 1 Insulin-like Growth Factor 1 Receptor (IGF1	Endothelial cells	NA
201131_s_at CDH1	200636_s_at PTPRF	0.646753246753247	4	R-HSA-6794362|R-HSA-8849932	Protein-protein interactions at synapses|Synaptic adhesion-like mo	Endothelial cells	Neutrophils;Endothelial cells
223752_at CFC1	208222_at ACVR1B	0.59266615737204	4	R-HSA-1181150|R-HSA-9006936	Signaling by NODAL|Signaling by TGF-beta family members	B lineage;Endothelial cells	T cells;B lineage;NK cells;Neutrophils;Endothelial cells;Fibr
233615_at CGA	202895_s_at SIRPA	0.565469824293354	4	R-HSA-1500931|R-HSA-202733	Cell-Cell communication|Cell surface interactions at the vascular	B lineage;Neutrophils;Fibroblasts	T cells;Neutrophils;Endothelial cells;Fibroblasts
206869_at CHAD	215879_at ITGB1	0.526355996944232	4	R-HSA-1500931|R-HSA-202733|R-HSA-	Cell-Cell communication|Cell surface interactions at the vascular	T cells;Myeloid dendritic cells;Neutrophils;Endothelial c	NK cells;Neutrophils;Endothelial cells
230045_at CNTN2	227209_at CNTN1	0.643697478991597	4	R-HSA-157118|R-HSA-373760	Signaling by NOTCH|L1CAM interactions	Neutrophils;Endothelial cells	T cells;Endothelial cells
229271_x_at COL11A1	215879_at ITGB1	0.573109243697479	4	R-HSA-1500931|R-HSA-202733|R-HSA-	Cell-Cell communication|Cell surface interactions at the vascular	NA	NK cells;Neutrophils;Endothelial cells
216865_at COL14A1	234418_x_at CD44	0.598472116119175	4	R-HSA-1474228|R-HSA-202733	Degradation of the extracellular matrix|Cell surface interactions a	B lineage;NK cells;Myeloid dendritic cells;Endothelial c	T cells;B lineage;NK cells;Myeloid dendritic cells;Neutrophi
202312_s_at COL1A1	209554_at CD36	0.555691367456073	4	R-HSA-114608|R-HSA-166016|R-HSA-1	Platelet degranulation|Toll Like Receptor 4 (TLR4) Cascade|MyD	T cells;NK cells;Neutrophils;Endothelial cells;Fibroblast	T cells;B lineage;NK cells;Myeloid dendritic cells;Neutrophi
202312_s_at COL1A1	234418_x_at CD44	0.620168067226891	4	R-HSA-1474228|R-HSA-202733	Degradation of the extracellular matrix|Cell surface interactions a	T cells;NK cells;Neutrophils;Endothelial cells;Fibroblast	T cells;B lineage;NK cells;Myeloid dendritic cells;Neutrophi
217430_x_at COL1A1	229902_at FLT4	0.538884644766998	4	R-HSA-194138	Signaling by VEGF	Neutrophils;Fibroblasts	Endothelial cells;Fibroblasts
202312_s_at COL1A1	215878_at ITGB1	0.562457026624331	4	R-HSA-202733|R-HSA-373755|R-HSA-3	Cell surface interactions at the vascular wall|Semaphorin interacti	T cells;NK cells;Neutrophils;Endothelial cells;Fibroblast	T cells
217430_x_at COL1A1	232941_at TMPRSS6	0.613750954927425	4	R-HSA-1474228	Degradation of the extracellular matrix	Neutrophils;Fibroblasts	T cells;B lineage;Myeloid dendritic cells;Neutrophils;Endoth

202403_s_at COL1A2	1560359_at ITGA1	0.501298701298701	4	R-HSA-373755|R-HSA-445355	Semaphorin interactions|Smooth Muscle Contraction	Fibroblasts	NA
202403_s_at COL1A2	204627_s_at ITGB3	0.504354469060351	4	R-HSA-194138|R-HSA-202733|R-HSA-3	Signaling by VEGF|Cell surface interactions at the vascular wall|I	Fibroblasts	NA
217404_s_at COL2A1	216956_s_at ITGA2B	0.546760397581862	4	R-HSA-114608|R-HSA-354192|R-HSA-3	Platelet degranulation|Integrin alphaIIb beta3 signaling|L1CAM in	B lineage;Endothelial cells;Fibroblasts	T cells;B lineage;Myeloid dendritic cells;Neutrophils;Endoth
217404_s_at COL2A1	215878_at ITGB1	0.636717856545392	4	R-HSA-202733|R-HSA-373755|R-HSA-3	Cell surface interactions at the vascular wall|Semaphorin interacti	B lineage;Endothelial cells;Fibroblasts	T cells
214641_at COL4A3	201474_s_at ITGA3	0.599694423223835	4	R-HSA-202733|R-HSA-6806834|R-HSA-	Cell surface interactions at the vascular wall|Signaling by MET|M	T cells;B lineage;NK cells;Myeloid dendritic cells;Neutr	T cells;Neutrophils;Endothelial cells
216896_at COL4A3	215879_at ITGB1	0.648483461285971	4	R-HSA-1500931|R-HSA-202733|R-HSA-	Cell-Cell communication|Cell surface interactions at the vascular	T cells;Neutrophils;Endothelial cells;Fibroblasts	NK cells;Neutrophils;Endothelial cells
216898_s_at COL4A3	204626_s_at ITGB3	0.685255920550038	4	R-HSA-194138|R-HSA-202733|R-HSA-3	Signaling by VEGF|Cell surface interactions at the vascular wall|I	Neutrophils;Endothelial cells	T cells;Myeloid dendritic cells;Neutrophils;Endothelial cells;
241565_at COL4A4	216190_x_at ITGB1	0.61100076394194	4	R-HSA-1500931|R-HSA-202733|R-HSA-	Cell-Cell communication|Cell surface interactions at the vascular	T cells;B lineage;NK cells;Neutrophils;Endothelial cells;	T cells;Neutrophils;Endothelial cells;Fibroblasts
241565_at COL4A4	211579_at ITGB3	0.732829095270386	4	R-HSA-194138|R-HSA-202733|R-HSA-2	Signaling by VEGF|Cell surface interactions at the vascular wall|I	T cells;B lineage;NK cells;Neutrophils;Endothelial cells;	T cells;B lineage;NK cells;Myeloid dendritic cells;Neutrophi
213110_s_at COL4A5	226016_at CD47	0.531550802139037	4	R-HSA-1500931|R-HSA-202733	Cell-Cell communication|Cell surface interactions at the vascular	Neutrophils;Endothelial cells	NA
213110_s_at COL4A5	227314_at ITGA2	0.59877769289534	4	R-HSA-373760|R-HSA-6806834|R-HSA-	L1CAM interactions|Signaling by MET|MET promotes cell motil	Neutrophils;Endothelial cells	Neutrophils
213110_s_at COL4A5	202351_at ITGAV	0.73048128342246	4	R-HSA-194138|R-HSA-202733|R-HSA-2	Signaling by VEGF|Cell surface interactions at the vascular wall|I	Neutrophils;Endothelial cells	Neutrophils;Endothelial cells
234387_at COL4A5	216190_x_at ITGB1	0.600305576776165	4	R-HSA-1500931|R-HSA-202733|R-HSA-	Cell-Cell communication|Cell surface interactions at the vascular	T cells	T cells;Neutrophils;Endothelial cells;Fibroblasts
210945_at COL4A6	215879_at ITGB1	0.655614973262032	4	R-HSA-1500931|R-HSA-202733|R-HSA-	Cell-Cell communication|Cell surface interactions at the vascular	T cells;B lineage;Myeloid dendritic cells;Neutrophils;En	NK cells;Neutrophils;Endothelial cells
212940_at COL6A1	229643_at ITGA6	0.719480519480519	4	R-HSA-1474290|R-HSA-2022090|R-HSA	Collagen formation|Assembly of collagen fibrils and other multim	Neutrophils;Fibroblasts	Fibroblasts
212938_at COL6A1	215878_at ITGB1	0.618076249034344	4	R-HSA-202733|R-HSA-373755|R-HSA-3	Cell surface interactions at the vascular wall|Semaphorin interacti	T cells;B lineage;Neutrophils;Endothelial cells;Fibroblas	T cells
217312_s_at COL7A1	215879_at ITGB1	0.608250572956455	4	R-HSA-1500931|R-HSA-202733|R-HSA-	Cell-Cell communication|Cell surface interactions at the vascular	T cells;B lineage;NK cells;Myeloid dendritic cells;Neutr	NK cells;Neutrophils;Endothelial cells
221152_at COL8A1	1560359_at ITGA1	0.619556913674561	4	R-HSA-373755|R-HSA-445355	Semaphorin interactions|Smooth Muscle Contraction	Endothelial cells	NA
1555527_at COL9A1	215879_at ITGB1	0.530328495034377	4	R-HSA-1500931|R-HSA-202733|R-HSA-	Cell-Cell communication|Cell surface interactions at the vascular	NA	NK cells;Neutrophils;Endothelial cells
232542_at COL9A2	216190_x_at ITGB1	0.605805958747135	4	R-HSA-1500931|R-HSA-202733|R-HSA-	Cell-Cell communication|Cell surface interactions at the vascular	T cells;Myeloid dendritic cells;Neutrophils;Endothelial c	T cells;Neutrophils;Endothelial cells;Fibroblasts
210182_at CORT	214491_at SSTR3	0.61038961038961	4	R-HSA-5620920|R-HSA-5620922	Cargo trafficking to the periciliary membrane|BBSome-mediated	T cells;Neutrophils;Endothelial cells;Fibroblasts	T cells;Neutrophils;Endothelial cells;Fibroblasts
205629_s_at CRH	214619_at CRHR1	0.559663865546218	4	R-HSA-373080	Class B/2 (Secretin family receptors)	T cells;B lineage;Myeloid dendritic cells;Neutrophils;En	B lineage;NK cells;Myeloid dendritic cells;Neutrophils;Endo
205629_s_at CRH	211510_s_at CRHR2	0.630252100840336	4	R-HSA-2980736	Peptide hormone metabolism	T cells;B lineage;Myeloid dendritic cells;Neutrophils;En	T cells;B lineage;Myeloid dendritic cells;Neutrophils;Endoth
210229_s_at CSF2	206009_at ITGA9	0.621084797555386	4	R-HSA-373760	L1CAM interactions	Endothelial cells	T cells;B lineage;NK cells;Neutrophils;Endothelial cells
210228_at CSF2	216190_x_at ITGB1	0.662235466826908	4	R-HSA-1500931|R-HSA-202733|R-HSA-	Cell-Cell communication|Cell surface interactions at the vascular	Myeloid dendritic cells;Neutrophils;Endothelial cells	T cells;Neutrophils;Endothelial cells;Fibroblasts
205958_x_at CSHL1	243755_at PRLR	0.722230710466005	4	R-HSA-1170546|R-HSA-982772	Prolactin receptor signaling|Growth hormone receptor signaling	T cells;B lineage;NK cells;Neutrophils;Endothelial cells;	T cells;B lineage;NK cells;Myeloid dendritic cells;Neutrophi
203666_at CXCL12	209869_at ADRA2A	0.645530939648587	4	R-HSA-163685|R-HSA-422356	Integration of energy metabolism|Regulation of insulin secretion	NA	Fibroblasts
209687_at CXCL12	203547_at CD4	0.537356760886173	4	R-HSA-162587|R-HSA-162909|R-HSA-2	HIV Life Cycle|Host Interactions of HIV factors|TCR signaling|C	T cells	Monocytic lineage
209687_at CXCL12	1553678_a_at ITGB1	0.509243697478992	4	R-HSA-1500931|R-HSA-202733|R-HSA-	Cell-Cell communication|Cell surface interactions at the vascular	T cells	NA
201289_at CYR61	212097_at CAV1	0.629640947288006	4	R-HSA-194138|R-HSA-4420097	Signaling by VEGF|VEGFA-VEGFR2 Pathway	NA	NA
242605_at DCN	211551_at EGFR	0.650114591291062	4	R-HSA-1227986|R-HSA-1236394|R-HSA	Signaling by ERBB2|Signaling by ERBB4|SHC1 events in ERBE	T cells;B lineage;Myeloid dendritic cells;Neutrophils;En	T cells;B lineage;Myeloid dendritic cells;Neutrophils;Endoth
239786_at DCN	211599_x_at MET	0.505004203475527	4	R-HSA-199418|R-HSA-2219528|R-HSA-	Negative regulation of the PI3K/AKT network|PI3K/AKT Signali	Fibroblasts	NA
1552730_at DHH	230035_at BOC	0.673338426279603	4	R-HSA-5632684	Hedgehog 'on' state	T cells;B lineage;Neutrophils;Endothelial cells;Fibroblas	T cells;B lineage;Myeloid dendritic cells;Neutrophils;Endoth
1552730_at DHH	208522_s_at PTCH1	0.667838044308633	4	R-HSA-5610787|R-HSA-5632684	Hedgehog 'off' state|Hedgehog 'on' state	T cells;B lineage;Neutrophils;Endothelial cells;Fibroblas	NK cells;Neutrophils;Endothelial cells
224215_s_at DLL1	218902_at NOTCH1	0.515660809778457	4	R-HSA-3781865	Diseases of glycosylation	Neutrophils	NA
227938_s_at DLL1	203237_s_at NOTCH3	0.560470625823973	4	R-HSA-3781865	Diseases of glycosylation	Endothelial cells	NA
227938_s_at DLL1	240786_at NOTCH4	0.556608097784568	4	R-HSA-157118|R-HSA-1912408|R-HSA-	Signaling by NOTCH|Pre-NOTCH Transcription and Translation	Endothelial cells	B lineage;Neutrophils;Endothelial cells;Fibroblasts
223525_at DLL4	240786_at NOTCH4	0.68301627519988	4	R-HSA-157118|R-HSA-1912408|R-HSA-	Signaling by NOTCH|Pre-NOTCH Transcription and Translation	T cells;B lineage;NK cells;Myeloid dendritic cells;Neutr	B lineage;Neutrophils;Endothelial cells;Fibroblasts
217067_s_at DMP1	211579_at ITGB3	0.622660250881323	4	R-HSA-194138|R-HSA-202733|R-HSA-2	Signaling by VEGF|Cell surface interactions at the vascular wall|I	B lineage;NK cells;Neutrophils;Endothelial cells;Fibrobl	T cells;B lineage;NK cells;Myeloid dendritic cells;Neutrophi
211484_s_at DSCAM	242653_at DCC	0.584721161191749	4	R-HSA-109581|R-HSA-373752|R-HSA-3	Apoptosis|Netrin-1 signaling|DSCAM interactions|DCC mediated	T cells;B lineage;NK cells;Myeloid dendritic cells;Neutr	T cells;NK cells;Myeloid dendritic cells;Neutrophils;Endothe
1553701_a_at DUSP18	201474_s_at ITGA3	0.660504201680672	4	R-HSA-202733|R-HSA-6806834|R-HSA-	Cell surface interactions at the vascular wall|Signaling by MET|M	T cells;B lineage;Myeloid dendritic cells;Neutrophils;En	T cells;Neutrophils;Endothelial cells
1553701_a_at DUSP18	215879_at ITGB1	0.622612681436211	4	R-HSA-1500931|R-HSA-202733|R-HSA-	Cell-Cell communication|Cell surface interactions at the vascular	T cells;B lineage;Myeloid dendritic cells;Neutrophils;En	NK cells;Neutrophils;Endothelial cells
1553701_a_at DUSP18	204989_s_at ITGB4	0.677922077922078	4	R-HSA-1474290|R-HSA-1500931|R-HSA	Collagen formation|Cell-Cell communication|Assembly of collage	T cells;B lineage;Myeloid dendritic cells;Neutrophils;En	T cells;Myeloid dendritic cells;Endothelial cells
225275_at EDIL3	202351_at ITGAV	0.778456837280367	4	R-HSA-194138|R-HSA-202733|R-HSA-2	Signaling by VEGF|Cell surface interactions at the vascular wall|I	Neutrophils;Endothelial cells	Neutrophils;Endothelial cells
225275_at EDIL3	201125_s_at ITGB5	0.600305576776165	4	R-HSA-445355	Smooth Muscle Contraction	Neutrophils;Endothelial cells	Endothelial cells
208256_at EFNA2	205977_s_at EPHA1	0.675783040488923	4	R-HSA-2892247|R-HSA-452723	POU5F1 (OCT4), SOX2, NANOG activate genes related to prolif	T cells;B lineage;Myeloid dendritic cells;Neutrophils;En	T cells;B lineage;NK cells;Myeloid dendritic cells;Neutrophi
210132_at EFNA3	205977_s_at EPHA1	0.560886172650879	4	R-HSA-2892247|R-HSA-452723	POU5F1 (OCT4), SOX2, NANOG activate genes related to prolif	T cells;Neutrophils;Endothelial cells;Fibroblasts	T cells;B lineage;NK cells;Myeloid dendritic cells;Neutrophi
233814_at EFNA5	205977_s_at EPHA1	0.526050420168067	4	R-HSA-2892247|R-HSA-452723	POU5F1 (OCT4), SOX2, NANOG activate genes related to prolif	NA	T cells;B lineage;NK cells;Myeloid dendritic cells;Neutrophi
210883_x_at EFNB3	210651_s_at EPHB2	0.682200152788388	4	R-HSA-2682334|R-HSA-373760|R-HSA-	EPH-Ephrin signaling|L1CAM interactions|EPHB-mediated forw	T cells;Myeloid dendritic cells;Neutrophils;Endothelial c	T cells;B lineage;Myeloid dendritic cells;Neutrophils;Endoth
210883_x_at EFNB3	1438_at EPHB3	0.684033613445378	4	R-HSA-2682334|R-HSA-3928662|R-HSA	EPH-Ephrin signaling|EPHB-mediated forward signaling|Ephrin s	T cells;Myeloid dendritic cells;Neutrophils;Endothelial c	T cells;B lineage;NK cells;Myeloid dendritic cells;Neutrophi
210883_x_at EFNB3	216680_s_at EPHB4	0.781818181818182	4	R-HSA-2682334|R-HSA-3928662|R-HSA	EPH-Ephrin signaling|EPHB-mediated forward signaling|Ephrin s	T cells;Myeloid dendritic cells;Neutrophils;Endothelial c	T cells;B lineage;Myeloid dendritic cells;Neutrophils;Endoth
206254_at EGF	201983_s_at EGFR	0.626279602750191	4	R-HSA-1227986|R-HSA-1236382|R-HSA	Signaling by ERBB2|Constitutive Signaling by Ligand-Responsiv	Neutrophils;Endothelial cells	Neutrophils;Endothelial cells
206254_at EGF	205710_at LRP2	0.538884644766998	4	R-HSA-196854|R-HSA-2187338|R-HSA-	Metabolism of vitamins and cofactors|Visual phototransduction|M	Neutrophils;Endothelial cells	Neutrophils;Endothelial cells
205767_at EREG	210984_x_at EGFR	0.527883880825057	4	R-HSA-1227986|R-HSA-1236382|R-HSA	Signaling by ERBB2|Constitutive Signaling by Ligand-Responsiv	Neutrophils;Fibroblasts	T cells;B lineage;NK cells;Myeloid dendritic cells;Neutrophi
205767_at EREG	210930_s_at ERBB2	0.675477463712758	4	R-HSA-1227986|R-HSA-1250196|R-HSA	Signaling by ERBB2|SHC1 events in ERBB2 signaling|Negative	Neutrophils;Fibroblasts	Myeloid dendritic cells;Neutrophils;Endothelial cells
205620_at F10	205785_at ITGAM	0.615584415584416	4	R-HSA-166016|R-HSA-168898|R-HSA-2	Toll Like Receptor 4 (TLR4) Cascade|Toll-Like Receptors Cascac	T cells;B lineage;Myeloid dendritic cells;Neutrophils;En	T cells;Neutrophils;Endothelial cells;Fibroblasts
1569592_a_at F11	207389_at GP1BA	0.656226126814362	4	R-HSA-140837|R-HSA-140877|R-HSA-7	Intrinsic Pathway of Fibrin Clot Formation|Formation of Fibrin C	T cells;B lineage;Monocytic lineage;Neutrophils;Endoth	Neutrophils;Endothelial cells
205754_at F2	207389_at GP1BA	0.553857906799083	4	R-HSA-140837|R-HSA-140877|R-HSA-7	Intrinsic Pathway of Fibrin Clot Formation|Formation of Fibrin C	T cells;B lineage;NK cells;Myeloid dendritic cells;Neutr	Neutrophils;Endothelial cells
205754_at F2	206883_x_at GP9	0.840488922841864	4	R-HSA-140837|R-HSA-140877|R-HSA-7	Intrinsic Pathway of Fibrin Clot Formation|Formation of Fibrin C	T cells;B lineage;NK cells;Myeloid dendritic cells;Neutr	T cells;B lineage;Myeloid dendritic cells;Neutrophils;Endoth
205754_at F2	216956_s_at ITGA2B	0.723257954654822	4	R-HSA-114608|R-HSA-354192|R-HSA-3	Platelet degranulation|Integrin alphaIIb beta3 signaling|L1CAM in	T cells;B lineage;NK cells;Myeloid dendritic cells;Neutr	T cells;B lineage;Myeloid dendritic cells;Neutrophils;Endoth
207218_at F9	1569042_at LRP1	0.533425014926797	4	R-HSA-196854|R-HSA-2168880|R-HSA-	Metabolism of vitamins and cofactors|Scavenging of heme from p	T cells;B lineage;Myeloid dendritic cells;Endothelial cel	T cells;B lineage;Myeloid dendritic cells;Neutrophils;Endoth
1554337_at FARP2	1558140_at PLXNA1	0.521048209939953	4	R-HSA-373755|R-HSA-399954|R-HSA-3	Semaphorin interactions|Sema3A PAK dependent Axon repulsion	T cells	T cells;B lineage;Myeloid dendritic cells;Neutrophils;Endoth
1554337_at FARP2	1553139_s_at PLXNA3	0.688569682151589	4	R-HSA-373755|R-HSA-399954|R-HSA-3	Semaphorin interactions|Sema3A PAK dependent Axon repulsion	T cells	T cells;B lineage;Myeloid dendritic cells;Neutrophils;Endoth
207834_at FBLN1	216190_x_at ITGB1	0.536481016158116	4	R-HSA-1500931|R-HSA-202733|R-HSA-	Cell-Cell communication|Cell surface interactions at the vascular	T cells;B lineage;NK cells;Myeloid dendritic cells;Neutr	T cells;Neutrophils;Endothelial cells;Fibroblasts
203886_s_at FBLN2	204627_s_at ITGB3	0.567608861726509	4	R-HSA-194138|R-HSA-202733|R-HSA-3	Signaling by VEGF|Cell surface interactions at the vascular wall|I	Fibroblasts	NA
208439_s_at FCN2	1569042_at LRP1	0.68530827612337	4	R-HSA-196854|R-HSA-2168880|R-HSA-	Metabolism of vitamins and cofactors|Scavenging of heme from p	T cells;B lineage;Myeloid dendritic cells;Neutrophils;En	T cells;B lineage;Myeloid dendritic cells;Neutrophils;Endoth
231671_at FGA	216956_s_at ITGA2B	0.702017123110976	4	R-HSA-114608|R-HSA-354192|R-HSA-3	Platelet degranulation|Integrin alphaIIb beta3 signaling|L1CAM in	T cells;B lineage;Myeloid dendritic cells;Neutrophils;En	T cells;B lineage;Myeloid dendritic cells;Neutrophils;Endoth

205650_s_at FGA	216190_x_at ITGB1	0.547135217723453	4	R-HSA-1500931|R-HSA-202733|R-HSA-	Cell-Cell communication|Cell surface interactions at the vascular	T cells;B lineage;NK cells;Myeloid dendritic cells;Neutr	T cells;Neutrophils;Endothelial cells;Fibroblasts
205650_s_at FGA	211579_at ITGB3	0.706853084804171	4	R-HSA-194138|R-HSA-202733|R-HSA-2	Signaling by VEGF|Cell surface interactions at the vascular wall|I	T cells;B lineage;NK cells;Myeloid dendritic cells;Neutr	T cells;B lineage;NK cells;Myeloid dendritic cells;Neutrophi
204988_at FGB	216956_s_at ITGA2B	0.727536683311136	4	R-HSA-114608|R-HSA-354192|R-HSA-3	Platelet degranulation|Integrin alphaIIb beta3 signaling|L1CAM in	T cells;B lineage;Myeloid dendritic cells;Neutrophils;En	T cells;B lineage;Myeloid dendritic cells;Neutrophils;Endoth
204988_at FGB	205785_at ITGAM	0.622001527883881	4	R-HSA-166016|R-HSA-168898|R-HSA-2	Toll Like Receptor 4 (TLR4) Cascade|Toll-Like Receptors Cascac	T cells;B lineage;Myeloid dendritic cells;Neutrophils;En	T cells;Neutrophils;Endothelial cells;Fibroblasts
216238_s_at FGB	215878_at ITGB1	0.651386662455725	4	R-HSA-202733|R-HSA-373755|R-HSA-3	Cell surface interactions at the vascular wall|Semaphorin interacti	T cells;B lineage;Myeloid dendritic cells;Neutrophils;En	T cells
204988_at FGB	211579_at ITGB3	0.725189092192088	4	R-HSA-194138|R-HSA-202733|R-HSA-2	Signaling by VEGF|Cell surface interactions at the vascular wall|I	T cells;B lineage;Myeloid dendritic cells;Neutrophils;En	T cells;B lineage;NK cells;Myeloid dendritic cells;Neutrophi
231762_at FGF10	207937_x_at FGFR1	0.627244252728302	4	R-HSA-1226099|R-HSA-1839124|R-HSA	Signaling by FGFR in disease|FGFR1 mutant receptor activation|S	T cells;B lineage;NK cells;Myeloid dendritic cells;Neutr	B lineage;Neutrophils;Endothelial cells;Fibroblasts
231762_at FGF10	211398_at FGFR2	0.73292589763178	4	R-HSA-1226099|R-HSA-1839126|R-HSA	Signaling by FGFR in disease|FGFR2 mutant receptor activation|,	T cells;B lineage;NK cells;Myeloid dendritic cells;Neutr	T cells;B lineage;Myeloid dendritic cells;Neutrophils;Endoth
231803_at FGF11	207937_x_at FGFR1	0.64359385931586	4	R-HSA-1226099|R-HSA-1839124|R-HSA	Signaling by FGFR in disease|FGFR1 mutant receptor activation|S	T cells;B lineage;NK cells;Myeloid dendritic cells;Neutr	B lineage;Neutrophils;Endothelial cells;Fibroblasts
231803_at FGF11	211398_at FGFR2	0.794346829640947	4	R-HSA-1226099|R-HSA-1839126|R-HSA	Signaling by FGFR in disease|FGFR2 mutant receptor activation|,	T cells;B lineage;NK cells;Myeloid dendritic cells;Neutr	T cells;B lineage;Myeloid dendritic cells;Neutrophils;Endoth
231803_at FGF11	204380_s_at FGFR3	0.534606569900688	4	R-HSA-1226099	Signaling by FGFR in disease	T cells;B lineage;NK cells;Myeloid dendritic cells;Neutr	Endothelial cells
231803_at FGF11	1554962_a_at FGFR4	0.661115355233002	4	R-HSA-109704|R-HSA-112399|R-HSA-1	PI3K Cascade|IRS-mediated signalling|Signaling by FGFR in disc	T cells;B lineage;NK cells;Myeloid dendritic cells;Neutr	Myeloid dendritic cells;Neutrophils;Endothelial cells;Fibrobl
214589_at FGF12	207937_x_at FGFR1	0.644816259808388	4	R-HSA-1226099|R-HSA-1839124|R-HSA	Signaling by FGFR in disease|FGFR1 mutant receptor activation|S	B lineage;Myeloid dendritic cells;Neutrophils;Endotheli	B lineage;Neutrophils;Endothelial cells;Fibroblasts
214589_at FGF12	211398_at FGFR2	0.620473644003056	4	R-HSA-1226099|R-HSA-1839126|R-HSA	Signaling by FGFR in disease|FGFR2 mutant receptor activation|,	B lineage;Myeloid dendritic cells;Neutrophils;Endotheli	T cells;B lineage;Myeloid dendritic cells;Neutrophils;Endoth
238521_at FGF12	204379_s_at FGFR3	0.601527883880825	4	R-HSA-1226099|R-HSA-5655332|R-HSA	Signaling by FGFR in disease|Signaling by FGFR3 in disease|Sig	NA	Neutrophils;Endothelial cells
214589_at FGF12	1554962_a_at FGFR4	0.541634835752483	4	R-HSA-109704|R-HSA-112399|R-HSA-1	PI3K Cascade|IRS-mediated signalling|Signaling by FGFR in disc	B lineage;Myeloid dendritic cells;Neutrophils;Endotheli	Myeloid dendritic cells;Neutrophils;Endothelial cells;Fibrobl
231523_at FGF14	207937_x_at FGFR1	0.508977005076242	4	R-HSA-1226099|R-HSA-1839124|R-HSA	Signaling by FGFR in disease|FGFR1 mutant receptor activation|S	T cells;Myeloid dendritic cells;Neutrophils;Endothelial c	B lineage;Neutrophils;Endothelial cells;Fibroblasts
231523_at FGF14	211398_at FGFR2	0.697478991596639	4	R-HSA-1226099|R-HSA-1839126|R-HSA	Signaling by FGFR in disease|FGFR2 mutant receptor activation|,	T cells;Myeloid dendritic cells;Neutrophils;Endothelial c	T cells;B lineage;Myeloid dendritic cells;Neutrophils;Endoth
231523_at FGF14	204380_s_at FGFR3	0.571886936592819	4	R-HSA-1226099	Signaling by FGFR in disease	T cells;Myeloid dendritic cells;Neutrophils;Endothelial c	Endothelial cells
221374_at FGF16	207937_x_at FGFR1	0.555733823915428	4	R-HSA-1226099|R-HSA-1839124|R-HSA	Signaling by FGFR in disease|FGFR1 mutant receptor activation|S	T cells;B lineage;NK cells;Myeloid dendritic cells;Neutr	B lineage;Neutrophils;Endothelial cells;Fibroblasts
221374_at FGF16	211398_at FGFR2	0.689533995416348	4	R-HSA-1226099|R-HSA-1839126|R-HSA	Signaling by FGFR in disease|FGFR2 mutant receptor activation|,	T cells;B lineage;NK cells;Myeloid dendritic cells;Neutr	T cells;B lineage;Myeloid dendritic cells;Neutrophils;Endoth
221374_at FGF16	1554962_a_at FGFR4	0.638197097020626	4	R-HSA-109704|R-HSA-112399|R-HSA-1	PI3K Cascade|IRS-mediated signalling|Signaling by FGFR in disc	T cells;B lineage;NK cells;Myeloid dendritic cells;Neutr	Myeloid dendritic cells;Neutrophils;Endothelial cells;Fibrobl
221376_at FGF17	211398_at FGFR2	0.820320855614973	4	R-HSA-1226099|R-HSA-1839126|R-HSA	Signaling by FGFR in disease|FGFR2 mutant receptor activation|,	B lineage;Neutrophils;Endothelial cells;Fibroblasts	T cells;B lineage;Myeloid dendritic cells;Neutrophils;Endoth
221376_at FGF17	1554962_a_at FGFR4	0.623529411764706	4	R-HSA-109704|R-HSA-112399|R-HSA-1	PI3K Cascade|IRS-mediated signalling|Signaling by FGFR in disc	B lineage;Neutrophils;Endothelial cells;Fibroblasts	Myeloid dendritic cells;Neutrophils;Endothelial cells;Fibrobl
214284_s_at FGF18	207937_x_at FGFR1	0.688364277354689	4	R-HSA-1226099|R-HSA-1839124|R-HSA	Signaling by FGFR in disease|FGFR1 mutant receptor activation|S	T cells;B lineage;NK cells;Myeloid dendritic cells;Neutr	B lineage;Neutrophils;Endothelial cells;Fibroblasts
214284_s_at FGF18	211398_at FGFR2	0.706340718105424	4	R-HSA-1226099|R-HSA-1839126|R-HSA	Signaling by FGFR in disease|FGFR2 mutant receptor activation|,	T cells;B lineage;NK cells;Myeloid dendritic cells;Neutr	T cells;B lineage;Myeloid dendritic cells;Neutrophils;Endoth
231382_at FGF18	204380_s_at FGFR3	0.514132925897632	4	R-HSA-1226099	Signaling by FGFR in disease	Myeloid dendritic cells;Neutrophils;Endothelial cells;Fil	Endothelial cells
214284_s_at FGF18	1554962_a_at FGFR4	0.638197097020626	4	R-HSA-109704|R-HSA-112399|R-HSA-1	PI3K Cascade|IRS-mediated signalling|Signaling by FGFR in disc	T cells;B lineage;NK cells;Myeloid dendritic cells;Neutr	Myeloid dendritic cells;Neutrophils;Endothelial cells;Fibrobl
223761_at FGF19	207937_x_at FGFR1	0.626327452358906	4	R-HSA-1226099|R-HSA-1839124|R-HSA	Signaling by FGFR in disease|FGFR1 mutant receptor activation|S	T cells;B lineage;Neutrophils;Endothelial cells;Fibroblas	B lineage;Neutrophils;Endothelial cells;Fibroblasts
223761_at FGF19	208229_at FGFR2	0.800152788388082	4	R-HSA-1226099|R-HSA-1839126|R-HSA	Signaling by FGFR in disease|FGFR2 mutant receptor activation|,	T cells;B lineage;Neutrophils;Endothelial cells;Fibroblas	B lineage;Neutrophils;Endothelial cells
223761_at FGF19	211237_s_at FGFR4	0.627807486631016	4	R-HSA-109704|R-HSA-112399|R-HSA-1	PI3K Cascade|IRS-mediated signalling|Signaling by FGFR in disc	T cells;B lineage;Neutrophils;Endothelial cells;Fibroblas	T cells;Neutrophils;Endothelial cells;Fibroblasts
208240_s_at FGF1	224999_at EGFR	0.705423987776929	4	R-HSA-1227986|R-HSA-157118|R-HSA-	Signaling by ERBB2|Signaling by NOTCH|Signaling by EGFR|N	Neutrophils;Endothelial cells	Neutrophils;Endothelial cells
205117_at FGF1	208228_s_at FGFR2	0.921842771427489	4	R-HSA-1226099|R-HSA-1839126|R-HSA	Signaling by FGFR in disease|FGFR2 mutant receptor activation|,	Neutrophils;Endothelial cells;Fibroblasts	Neutrophils;Endothelial cells
205117_at FGF1	204379_s_at FGFR3	0.8768525592055	4	R-HSA-1226099|R-HSA-5655332|R-HSA	Signaling by FGFR in disease|Signaling by FGFR3 in disease|Sig	Neutrophils;Endothelial cells;Fibroblasts	Neutrophils;Endothelial cells
208240_s_at FGF1	223321_s_at FGFRL1	0.580137509549274	4	R-HSA-190236|R-HSA-5654736	Signaling by FGFR|Signaling by FGFR1	Neutrophils;Endothelial cells	NA
221433_at FGF21	211398_at FGFR2	0.737565897178931	4	R-HSA-1226099|R-HSA-1839126|R-HSA	Signaling by FGFR in disease|FGFR2 mutant receptor activation|,	T cells;B lineage;NK cells;Myeloid dendritic cells;Neutr	T cells;B lineage;Myeloid dendritic cells;Neutrophils;Endoth
221433_at FGF21	204380_s_at FGFR3	0.576973032473098	4	R-HSA-1226099	Signaling by FGFR in disease	T cells;B lineage;NK cells;Myeloid dendritic cells;Neutr	Endothelial cells
221433_at FGF21	1554962_a_at FGFR4	0.699213081725873	4	R-HSA-109704|R-HSA-112399|R-HSA-1	PI3K Cascade|IRS-mediated signalling|Signaling by FGFR in disc	T cells;B lineage;NK cells;Myeloid dendritic cells;Neutr	Myeloid dendritic cells;Neutrophils;Endothelial cells;Fibrobl
1566814_at FGF22	207937_x_at FGFR1	0.586293836228622	4	R-HSA-1226099|R-HSA-1839124|R-HSA	Signaling by FGFR in disease|FGFR1 mutant receptor activation|S	T cells;B lineage;NK cells;Myeloid dendritic cells;Neutr	B lineage;Neutrophils;Endothelial cells;Fibroblasts
1566814_at FGF22	208229_at FGFR2	0.681283422459893	4	R-HSA-1226099|R-HSA-1839126|R-HSA	Signaling by FGFR in disease|FGFR2 mutant receptor activation|,	T cells;B lineage;NK cells;Myeloid dendritic cells;Neutr	B lineage;Neutrophils;Endothelial cells
221166_at FGF23	207937_x_at FGFR1	0.509627139364303	4	R-HSA-1226099|R-HSA-1839124|R-HSA	Signaling by FGFR in disease|FGFR1 mutant receptor activation|S	T cells;B lineage;NK cells;Neutrophils;Endothelial cells;	B lineage;Neutrophils;Endothelial cells;Fibroblasts
221166_at FGF23	211398_at FGFR2	0.726411492684615	4	R-HSA-1226099|R-HSA-1839126|R-HSA	Signaling by FGFR in disease|FGFR2 mutant receptor activation|,	T cells;B lineage;NK cells;Neutrophils;Endothelial cells;	T cells;B lineage;Myeloid dendritic cells;Neutrophils;Endoth
221166_at FGF23	1554962_a_at FGFR4	0.608144245032556	4	R-HSA-109704|R-HSA-112399|R-HSA-1	PI3K Cascade|IRS-mediated signalling|Signaling by FGFR in disc	T cells;B lineage;NK cells;Neutrophils;Endothelial cells;	Myeloid dendritic cells;Neutrophils;Endothelial cells;Fibrobl
204422_s_at FGF2	208228_s_at FGFR2	0.58598823610549	4	R-HSA-1226099|R-HSA-1839126|R-HSA	Signaling by FGFR in disease|FGFR2 mutant receptor activation|,	Endothelial cells	Neutrophils;Endothelial cells
204422_s_at FGF2	204379_s_at FGFR3	0.557219251336898	4	R-HSA-1226099|R-HSA-5655332|R-HSA	Signaling by FGFR in disease|Signaling by FGFR3 in disease|Sig	Endothelial cells	Neutrophils;Endothelial cells
204422_s_at FGF2	212158_at SDC2	0.574637127578304	4	R-HSA-1630316|R-HSA-1638091|R-HSA	Glycosaminoglycan metabolism|Heparan sulfate/heparin (HS-GA	Endothelial cells	Endothelial cells
214571_at FGF3	207937_x_at FGFR1	0.540606617820397	4	R-HSA-1226099|R-HSA-1839124|R-HSA	Signaling by FGFR in disease|FGFR1 mutant receptor activation|S	Neutrophils	B lineage;Neutrophils;Endothelial cells;Fibroblasts
214571_at FGF3	211398_at FGFR2	0.585637891520244	4	R-HSA-1226099|R-HSA-1839126|R-HSA	Signaling by FGFR in disease|FGFR2 mutant receptor activation|,	Neutrophils	T cells;B lineage;Myeloid dendritic cells;Neutrophils;Endoth
206783_at FGF4	207937_x_at FGFR1	0.62388265137385	4	R-HSA-1226099|R-HSA-1839124|R-HSA	Signaling by FGFR in disease|FGFR1 mutant receptor activation|S	T cells;Myeloid dendritic cells;Neutrophils;Endothelial c	B lineage;Neutrophils;Endothelial cells;Fibroblasts
206783_at FGF4	211398_at FGFR2	0.684033613445378	4	R-HSA-1226099|R-HSA-1839126|R-HSA	Signaling by FGFR in disease|FGFR2 mutant receptor activation|,	T cells;Myeloid dendritic cells;Neutrophils;Endothelial c	T cells;B lineage;Myeloid dendritic cells;Neutrophils;Endoth
206783_at FGF4	204380_s_at FGFR3	0.563025210084034	4	R-HSA-1226099	Signaling by FGFR in disease	T cells;Myeloid dendritic cells;Neutrophils;Endothelial c	Endothelial cells
206783_at FGF4	1554962_a_at FGFR4	0.670893812070283	4	R-HSA-109704|R-HSA-112399|R-HSA-1	PI3K Cascade|IRS-mediated signalling|Signaling by FGFR in disc	T cells;Myeloid dendritic cells;Neutrophils;Endothelial c	Myeloid dendritic cells;Neutrophils;Endothelial cells;Fibrobl
206783_at FGF4	210615_at NRP1	0.703896103896104	4	R-HSA-194138|R-HSA-373755|R-HSA-3	Signaling by VEGF|Semaphorin interactions|L1CAM interactions	T cells;Myeloid dendritic cells;Neutrophils;Endothelial c	T cells;B lineage;Myeloid dendritic cells;Neutrophils;Endoth
208378_x_at FGF5	207937_x_at FGFR1	0.724883492068956	4	R-HSA-1226099|R-HSA-1839124|R-HSA	Signaling by FGFR in disease|FGFR1 mutant receptor activation|S	T cells;B lineage;Neutrophils;Endothelial cells;Fibroblas	B lineage;Neutrophils;Endothelial cells;Fibroblasts
210311_at FGF5	211398_at FGFR2	0.724064171122995	4	R-HSA-1226099|R-HSA-1839126|R-HSA	Signaling by FGFR in disease|FGFR2 mutant receptor activation|,	B lineage;Neutrophils;Endothelial cells	T cells;B lineage;Myeloid dendritic cells;Neutrophils;Endoth
210310_s_at FGF5	211237_s_at FGFR4	0.647872261039708	4	R-HSA-109704|R-HSA-112399|R-HSA-1	PI3K Cascade|IRS-mediated signalling|Signaling by FGFR in disc	B lineage;Neutrophils;Endothelial cells	T cells;Neutrophils;Endothelial cells;Fibroblasts
208417_at FGF6	207937_x_at FGFR1	0.69248987901697	4	R-HSA-1226099|R-HSA-1839124|R-HSA	Signaling by FGFR in disease|FGFR1 mutant receptor activation|S	T cells;B lineage;Myeloid dendritic cells;Neutrophils;En	B lineage;Neutrophils;Endothelial cells;Fibroblasts
208417_at FGF6	211398_at FGFR2	0.719480519480519	4	R-HSA-1226099|R-HSA-1839126|R-HSA	Signaling by FGFR in disease|FGFR2 mutant receptor activation|,	T cells;B lineage;Myeloid dendritic cells;Neutrophils;En	T cells;B lineage;Myeloid dendritic cells;Neutrophils;Endoth
208417_at FGF6	204380_s_at FGFR3	0.504354469060351	4	R-HSA-1226099	Signaling by FGFR in disease	T cells;B lineage;Myeloid dendritic cells;Neutrophils;En	Endothelial cells
208417_at FGF6	1554962_a_at FGFR4	0.685867074102368	4	R-HSA-109704|R-HSA-112399|R-HSA-1	PI3K Cascade|IRS-mediated signalling|Signaling by FGFR in disc	T cells;B lineage;Myeloid dendritic cells;Neutrophils;En	Myeloid dendritic cells;Neutrophils;Endothelial cells;Fibrobl
1555102_at FGF7	207937_x_at FGFR1	0.607380244724726	4	R-HSA-1226099|R-HSA-1839124|R-HSA	Signaling by FGFR in disease|FGFR1 mutant receptor activation|S	Neutrophils;Endothelial cells	B lineage;Neutrophils;Endothelial cells;Fibroblasts
1555102_at FGF7	211398_at FGFR2	0.677922077922078	4	R-HSA-1226099|R-HSA-1839126|R-HSA	Signaling by FGFR in disease|FGFR2 mutant receptor activation|,	Neutrophils;Endothelial cells	T cells;B lineage;Myeloid dendritic cells;Neutrophils;Endoth
1555102_at FGF7	1554961_at FGFR4	0.621084797555386	4	R-HSA-109704|R-HSA-112399|R-HSA-1	PI3K Cascade|IRS-mediated signalling|Signaling by FGFR in disc	Neutrophils;Endothelial cells	NA
1555102_at FGF7	210615_at NRP1	0.573414820473644	4	R-HSA-194138|R-HSA-373755|R-HSA-3	Signaling by VEGF|Semaphorin interactions|L1CAM interactions	Neutrophils;Endothelial cells	T cells;B lineage;Myeloid dendritic cells;Neutrophils;Endoth
208449_s_at FGF8	211237_s_at FGFR4	0.508326967150497	4	R-HSA-109704|R-HSA-112399|R-HSA-1	PI3K Cascade|IRS-mediated signalling|Signaling by FGFR in disc	NA	T cells;Neutrophils;Endothelial cells;Fibroblasts
219612_s_at FGG	216966_at ITGA2B	0.59266615737204	4	R-HSA-373760|R-HSA-5674135|R-HSA-	L1CAM interactions|MAP2K and MAPK activation|Signaling by	B lineage;Neutrophils;Fibroblasts	Neutrophils
219612_s_at FGG	211579_at ITGB3	0.66636106848919	4	R-HSA-194138|R-HSA-202733|R-HSA-2	Signaling by VEGF|Cell surface interactions at the vascular wall|I	B lineage;Neutrophils;Fibroblasts	T cells;B lineage;NK cells;Myeloid dendritic cells;Neutrophi

214701_s_at FN1	234418_x_at CD44	0.513216195569137	4	R-HSA-1474228|R-HSA-202733	Degradation of the extracellular matrix|Cell surface interactions a	Neutrophils	T cells;B lineage;NK cells;Myeloid dendritic cells;Neutrophi
214701_s_at FN1	211809_x_at COL13A1	0.504393003229263	4	R-HSA-1474290|R-HSA-1650814	Collagen formation|Collagen biosynthesis and modifying enzyme	Neutrophils	NA
212464_s_at FN1	1553678_a_at ITGB1	0.501909854851031	4	R-HSA-1500931|R-HSA-202733|R-HSA-	Cell-Cell communication|Cell surface interactions at the vascular	Fibroblasts	NA
214701_s_at FN1	204627_s_at ITGB3	0.528495034377387	4	R-HSA-194138|R-HSA-202733|R-HSA-3	Signaling by VEGF|Cell surface interactions at the vascular wall|I	Neutrophils	NA
214489_at FSHB	205019_s_at VIPR1	0.642169595110772	4	R-HSA-373080|R-HSA-420092	Class B/2 (Secretin family receptors)|Glucagon-type ligand recept	T cells;B lineage;Neutrophils;Endothelial cells;Fibroblas	NA
202177_at GAS6	202686_s_at AXL	0.71825821237586	4	R-HSA-194138|R-HSA-4420097	Signaling by VEGF|VEGFA-VEGFR2 Pathway	T cells;Neutrophils;Endothelial cells	T cells;Monocytic lineage;Endothelial cells
202177_at GAS6	206028_s_at MERTK	0.546829640947288	4	R-HSA-202733	Cell surface interactions at the vascular wall	T cells;Neutrophils;Endothelial cells	Monocytic lineage
206422_at GCG	207151_at ADCYAP1R1	0.501909854851031	4	R-HSA-166520|R-HSA-187037|R-HSA-3	Signaling by NTRKs|Signaling by NTRK1 (TRKA)|Class B/2 (Se	Endothelial cells	T cells
206422_at GCG	208400_at GLP1R	0.693200916730329	4	R-HSA-163685|R-HSA-373080|R-HSA-3	Integration of energy metabolism|Class B/2 (Secretin family recep	Endothelial cells	T cells;B lineage;Myeloid dendritic cells;Neutrophils;Endoth
206159_at GDF10	208222_at ACVR1B	0.615889992360581	4	R-HSA-1181150|R-HSA-9006936	Signaling by NODAL|Signaling by TGF-beta family members	T cells;B lineage;Neutrophils;Endothelial cells;Fibroblas	T cells;B lineage;NK cells;Neutrophils;Endothelial cells;Fibr
216854_at GDF11	208222_at ACVR1B	0.669671504965623	4	R-HSA-1181150|R-HSA-9006936	Signaling by NODAL|Signaling by TGF-beta family members	T cells;B lineage;NK cells;Myeloid dendritic cells;Neutr	T cells;B lineage;NK cells;Neutrophils;Endothelial cells;Fibr
216854_at GDF11	1559548_at ACVR2B	0.515966386554622	4	R-HSA-1181150|R-HSA-1502540|R-HSA	Signaling by NODAL|Signaling by Activin|Signaling by BMP|Sig	T cells;B lineage;NK cells;Myeloid dendritic cells;Neutr	B lineage;NK cells;Myeloid dendritic cells;Neutrophils;Endo
216854_at GDF11	230979_at BMPR1A	0.557219251336898	4	R-HSA-201451|R-HSA-9006936	Signaling by BMP|Signaling by TGF-beta family members	T cells;B lineage;NK cells;Myeloid dendritic cells;Neutr	B lineage;Neutrophils;Endothelial cells;Fibroblasts
216854_at GDF11	210523_at BMPR1B	0.695645530939649	4	R-HSA-201451|R-HSA-9006936	Signaling by BMP|Signaling by TGF-beta family members	T cells;B lineage;NK cells;Myeloid dendritic cells;Neutr	T cells;Myeloid dendritic cells;Neutrophils;Endothelial cells;
216854_at GDF11	1556075_at BMPR2	0.656531703590527	4	R-HSA-201451|R-HSA-9006936	Signaling by BMP|Signaling by TGF-beta family members	T cells;B lineage;NK cells;Myeloid dendritic cells;Neutr	T cells;B lineage;Myeloid dendritic cells;Neutrophils;Endoth
221314_at GDF9	210523_at BMPR1B	0.730786860198625	4	R-HSA-201451|R-HSA-9006936	Signaling by BMP|Signaling by TGF-beta family members	T cells;Neutrophils;Endothelial cells;Fibroblasts	T cells;Myeloid dendritic cells;Neutrophils;Endothelial cells;
221314_at GDF9	1556075_at BMPR2	0.586554621848739	4	R-HSA-201451|R-HSA-9006936	Signaling by BMP|Signaling by TGF-beta family members	T cells;Neutrophils;Endothelial cells;Fibroblasts	T cells;B lineage;Myeloid dendritic cells;Neutrophils;Endoth
221314_at GDF9	230347_at ORAI2	0.655920550038197	4	R-HSA-418346|R-HSA-5576891|R-HSA-	Platelet homeostasis|Cardiac conduction|Ion homeostasis|Antigen	T cells;Neutrophils;Endothelial cells;Fibroblasts	T cells;B lineage;Myeloid dendritic cells;Neutrophils;Endoth
221359_at GDNF	205696_s_at GFRA1	0.525439266615737	4	R-HSA-8853659	RET signaling	B lineage;NK cells;Myeloid dendritic cells;Neutrophils;I	NA
221359_at GDNF	215771_x_at RET	0.760427807486631	4	R-HSA-8853659	RET signaling	B lineage;NK cells;Myeloid dendritic cells;Neutrophils;I	T cells;B lineage;Myeloid dendritic cells;Neutrophils;Endoth
211151_x_at GH1	243755_at PRLR	0.536440030557678	4	R-HSA-1170546|R-HSA-982772	Prolactin receptor signaling|Growth hormone receptor signaling	B lineage;Neutrophils;Endothelial cells	T cells;B lineage;NK cells;Myeloid dendritic cells;Neutrophi
237647_at GHRL	211545_at GHRHR	0.777845683728037	4	R-HSA-373080|R-HSA-420092	Class B/2 (Secretin family receptors)|Glucagon-type ligand recept	T cells;B lineage;NK cells;Myeloid dendritic cells;Neutr	T cells;B lineage;Myeloid dendritic cells;Neutrophils;Endoth
207899_at GIP	208105_at GIPR	0.548399420960262	4	R-HSA-373080|R-HSA-420092	Class B/2 (Secretin family receptors)|Glucagon-type ligand recept	NA	T cells;B lineage;Myeloid dendritic cells;Neutrophils;Endoth
239037_at GNAS	215340_at ADCY1	0.803514132925898	4	R-HSA-111885|R-HSA-111931|R-HSA-1	Opioid Signalling|PKA-mediated phosphorylation of CREB|Calm	T cells;B lineage;Myeloid dendritic cells;Neutrophils;En	T cells;B lineage;Myeloid dendritic cells;Neutrophils;Endoth
228173_at GNAS	203741_s_at ADCY7	0.536745607333843	4	R-HSA-111885|R-HSA-111931|R-HSA-1	Opioid Signalling|PKA-mediated phosphorylation of CREB|Calm	Neutrophils	NA
217057_s_at GNAS	215400_x_at ADCY9	0.600809842077389	4	R-HSA-111885|R-HSA-111931|R-HSA-1	Opioid Signalling|PKA-mediated phosphorylation of CREB|Calm	T cells;Myeloid dendritic cells;Neutrophils;Endothelial c	T cells;B lineage;Neutrophils;Endothelial cells;Fibroblasts
217058_at GNAS	208111_at AVPR2	0.689839572192513	4	R-HSA-432040|R-HSA-445717|R-HSA-5	Vasopressin regulates renal water homeostasis via Aquaporins|Aq	Endothelial cells;Fibroblasts	Endothelial cells;Fibroblasts
217057_s_at GNAS	214619_at CRHR1	0.687700534759358	4	R-HSA-373080	Class B/2 (Secretin family receptors)	T cells;Myeloid dendritic cells;Neutrophils;Endothelial c	B lineage;NK cells;Myeloid dendritic cells;Neutrophils;Endo
239037_at GNAS	208390_s_at GLP1R	0.717952635599694	4	R-HSA-163685|R-HSA-373080|R-HSA-3	Integration of energy metabolism|Class B/2 (Secretin family recep	T cells;B lineage;Myeloid dendritic cells;Neutrophils;En	T cells;B lineage;NK cells;Myeloid dendritic cells;Neutrophi
239037_at GNAS	206187_at PTGIR	0.700840336134454	4	R-HSA-418346	Platelet homeostasis	T cells;B lineage;Myeloid dendritic cells;Neutrophils;En	T cells;B lineage;NK cells;Myeloid dendritic cells;Neutrophi
217057_s_at GNAS	205019_s_at VIPR1	0.671810542398778	4	R-HSA-373080|R-HSA-420092	Class B/2 (Secretin family receptors)|Glucagon-type ligand recept	T cells;Myeloid dendritic cells;Neutrophils;Endothelial c	NA
227059_at GPC6	200785_s_at LRP1	0.542857142857143	4	R-HSA-196854|R-HSA-2187338|R-HSA-	Metabolism of vitamins and cofactors|Visual phototransduction|M	NA	NA
210164_at GZMB	201393_s_at IGF2R	0.7231474407945	4	R-HSA-199992|R-HSA-421837|R-HSA-6	trans-Golgi Network Vesicle Budding|Clathrin derived vesicle bu	Cytotoxic lymphocytes	Monocytic lineage
244857_at HBEGF	1565484_x_at EGFR	0.575859434682964	4	R-HSA-1227986|R-HSA-1236382|R-HSA	Signaling by ERBB2|Constitutive Signaling by Ligand-Responsiv	NA	T cells;B lineage;Myeloid dendritic cells;Neutrophils;Endoth
222076_at HBEGF	234354_x_at ERBB2	0.528189457601222	4	R-HSA-1227986|R-HSA-1250196|R-HSA	Signaling by ERBB2|SHC1 events in ERBB2 signaling|PI3K eve	Neutrophils	T cells;Myeloid dendritic cells;Neutrophils;Endothelial cells;
222076_at HBEGF	206346_at PRLR	0.528800611533552	4	R-HSA-982772	Growth hormone receptor signaling	Neutrophils	B lineage;Neutrophils;Endothelial cells;Fibroblasts
209960_at HGF	211945_s_at ITGB1	0.548663101604278	4	R-HSA-1500931|R-HSA-202733|R-HSA-	Cell-Cell communication|Cell surface interactions at the vascular	NA	NA
215313_x_at HLA-A	208248_x_at APLP2	0.620473644003056	4	R-HSA-381426|R-HSA-8957275	Regulation of Insulin-like Growth Factor (IGF) transport and upta	Monocytic lineage	NA
208729_x_at HLA-B	208853_s_at CANX	0.641558441558442	4	R-HSA-2132295|R-HSA-447115|R-HSA-	MHC class II antigen presentation|Interleukin-12 family signaling	NA	Cytotoxic lymphocytes
211529_x_at HLA-G	203547_at CD4	0.510160427807487	4	R-HSA-162587|R-HSA-162909|R-HSA-2	HIV Life Cycle|Host Interactions of HIV factors|TCR signaling|C	Cytotoxic lymphocytes	Monocytic lineage
39763_at HPX	1569042_at LRP1	0.72864782276547	4	R-HSA-196854|R-HSA-2168880|R-HSA-	Metabolism of vitamins and cofactors|Scavenging of heme from p	T cells;B lineage;Myeloid dendritic cells;Neutrophils;En	T cells;B lineage;Myeloid dendritic cells;Neutrophils;Endoth
216449_x_at HSP90B1	223903_at TLR9	0.650725744843392	4	R-HSA-168138|R-HSA-168181|R-HSA-1	Toll Like Receptor 9 (TLR9) Cascade|Toll Like Receptor 7/8 (TL	NA	NA
201655_s_at HSPG2	1559634_at CHRM3	0.634835752482811	4	R-HSA-163685|R-HSA-422356	Integration of energy metabolism|Regulation of insulin secretion	Neutrophils;Endothelial cells;Fibroblasts	Neutrophils;Endothelial cells
201655_s_at HSPG2	211809_x_at COL13A1	0.532355414495835	4	R-HSA-1474290|R-HSA-1650814	Collagen formation|Collagen biosynthesis and modifying enzyme	Neutrophils;Endothelial cells;Fibroblasts	NA
207370_at IBSP	204625_s_at ITGB3	0.642475171886937	4	R-HSA-194138|R-HSA-202733|R-HSA-3	Signaling by VEGF|Cell surface interactions at the vascular wall|I	T cells;B lineage;Neutrophils;Endothelial cells;Fibroblas	T cells;B lineage;Neutrophils;Fibroblasts
215485_s_at ICAM1	211607_x_at EGFR	0.763789152024446	4	R-HSA-1227986|R-HSA-1236382|R-HSA	Signaling by ERBB2|Constitutive Signaling by Ligand-Responsiv	T cells;B lineage;NK cells;Myeloid dendritic cells;Neutr	T cells;B lineage;NK cells;Myeloid dendritic cells;Neutrophi
202638_s_at ICAM1	1555349_a_at ITGB2	0.611917494270435	4	R-HSA-166016|R-HSA-168898|R-HSA-2	Toll Like Receptor 4 (TLR4) Cascade|Toll-Like Receptors Cascac	NA	Monocytic lineage
209542_x_at IGF1	243358_at IGF1R	0.560580595874714	4	R-HSA-2404192|R-HSA-2428924|R-HSA	Signaling by Type 1 Insulin-like Growth Factor 1 Receptor (IGF1	NA	T cells;NK cells;Myeloid dendritic cells;Endothelial cells
241072_s_at IGF1	207851_s_at INSR	0.656226126814362	4	R-HSA-199418|R-HSA-6811558|R-HSA-	Negative regulation of the PI3K/AKT network|PI5P, PP2A and IE	T cells;B lineage;Myeloid dendritic cells;Neutrophils;En	Neutrophils;Fibroblasts
215420_at IHH	230035_at BOC	0.713063407181054	4	R-HSA-5632684	Hedgehog 'on' state	T cells;B lineage;Myeloid dendritic cells;Neutrophils;En	T cells;B lineage;Myeloid dendritic cells;Neutrophils;Endoth
229358_at IHH	1563677_at CDON	0.552330022918258	4	R-HSA-375170|R-HSA-525793|R-HSA-5	CDO in myogenesis|Myogenesis|Hedgehog 'on' state	Neutrophils;Endothelial cells	T cells;Neutrophils;Endothelial cells
215420_at IHH	238754_at PTCH1	0.854305144215331	4	R-HSA-5610787|R-HSA-5632684	Hedgehog 'off' state|Hedgehog 'on' state	T cells;B lineage;Myeloid dendritic cells;Neutrophils;En	T cells;B lineage;Myeloid dendritic cells;Neutrophils;Endoth
207433_at IL10	209575_at IL10RB	0.634224598930481	4	R-HSA-6783783	Interleukin-10 signaling	NA	NA
206926_s_at IL11	1552646_at IL11RA	0.688517077416255	4	R-HSA-6783589	Interleukin-6 family signaling	T cells;B lineage;NK cells;Myeloid dendritic cells;Neutr	T cells;B lineage;Myeloid dendritic cells;Neutrophils;Endoth
1555016_at IL16	216424_at CD4	0.570664629488159	4	R-HSA-162587|R-HSA-162909|R-HSA-2	HIV Life Cycle|Host Interactions of HIV factors|TCR signaling|D	T cells;B lineage;Myeloid dendritic cells;Neutrophils;En	B lineage;Endothelial cells
1555016_at IL16	207036_x_at GRIN2D	0.576209032165268	4	R-HSA-112314|R-HSA-438064|R-HSA-4	Neurotransmitter receptors and postsynaptic signal transmission|F	T cells;B lineage;Myeloid dendritic cells;Neutrophils;En	T cells;Neutrophils;Endothelial cells
1555016_at IL16	230585_at KCNJ15	0.695851480371422	4	R-HSA-112314	Neurotransmitter receptors and postsynaptic signal transmission	T cells;B lineage;Myeloid dendritic cells;Neutrophils;En	B lineage;Neutrophils;Endothelial cells
224262_at IL1F10	208038_at IL1RL2	0.642780748663102	4	R-HSA-446652|R-HSA-9014826	Interleukin-1 family signaling|Interleukin-36 pathway	T cells;B lineage;Neutrophils;Endothelial cells;Fibroblas	T cells;B lineage;Neutrophils;Endothelial cells;Fibroblasts
216244_at IL1RN	208038_at IL1RL2	0.644308632543927	4	R-HSA-446652|R-HSA-9014826	Interleukin-1 family signaling|Interleukin-36 pathway	T cells;B lineage;NK cells;Myeloid dendritic cells;Neutr	T cells;B lineage;Neutrophils;Endothelial cells;Fibroblasts
224071_at IL20	222828_at IL20RA	0.572498090145149	4	R-HSA-8854691	Interleukin-20 family signaling	T cells;B lineage;Neutrophils;Endothelial cells;Fibroblas	T cells;B lineage;NK cells;Myeloid dendritic cells;Neutrophi
222974_at IL22	237493_at IL22RA2	0.583193277310924	4	R-HSA-8854691	Interleukin-20 family signaling	NA	NA
234377_at IL23A	206890_at IL12RB1	0.646142093200917	4	R-HSA-447115|R-HSA-9020591	Interleukin-12 family signaling|Interleukin-12 signaling	T cells;Neutrophils;Endothelial cells;Fibroblasts	T cells;Neutrophils;Endothelial cells;Fibroblasts
220054_at IL23A	1561853_a_at IL23R	0.540718105423988	4	R-HSA-447115|R-HSA-6785807	Interleukin-12 family signaling|Interleukin-4 and 13 signaling	Neutrophils;Endothelial cells	Endothelial cells
221404_at IL36A	208038_at IL1RL2	0.611047446202309	4	R-HSA-446652|R-HSA-9014826	Interleukin-1 family signaling|Interleukin-36 pathway	T cells;B lineage;NK cells;Myeloid dendritic cells;Neutr	T cells;B lineage;Neutrophils;Endothelial cells;Fibroblasts
231755_at IL36B	208038_at IL1RL2	0.651948051948052	4	R-HSA-446652|R-HSA-9014826	Interleukin-1 family signaling|Interleukin-36 pathway	T cells;B lineage;NK cells;Myeloid dendritic cells;Neutr	T cells;B lineage;Neutrophils;Endothelial cells;Fibroblasts
220322_at IL36G	208038_at IL1RL2	0.566997708174179	4	R-HSA-446652|R-HSA-9014826	Interleukin-1 family signaling|Interleukin-36 pathway	Neutrophils	T cells;B lineage;Neutrophils;Endothelial cells;Fibroblasts
207952_at IL5	211516_at IL5RA	0.616089848233986	4	R-HSA-392451|R-HSA-397795|R-HSA-5	G beta:gamma signalling through PI3Kgamma|G-protein beta:gan	NA	NA

206598_at INS	243358_at IGF1R	0.582582123758594	4	R-HSA-2404192|R-HSA-2428924|R-HSA	Signaling by Type 1 Insulin-like Growth Factor 1 Receptor (IGF1	B lineage;Myeloid dendritic cells;Neutrophils;Endothelia	T cells;NK cells;Myeloid dendritic cells;Endothelial cells
206598_at INS	207851_s_at INSR	0.511382734912147	4	R-HSA-199418|R-HSA-6811558|R-HSA-	Negative regulation of the PI3K/AKT network|PI5P, PP2A and IE	B lineage;Myeloid dendritic cells;Neutrophils;Endothelia	Neutrophils;Fibroblasts
216268_s_at JAG1	212377_s_at NOTCH2	0.562108479755539	4	R-HSA-157118|R-HSA-1980145|R-HSA-	Signaling by NOTCH|Signaling by NOTCH2|Diseases of glycosy	Endothelial cells;Fibroblasts	NA
209097_s_at JAG1	240786_at NOTCH4	0.661726508785332	4	R-HSA-157118|R-HSA-1912408|R-HSA-	Signaling by NOTCH|Pre-NOTCH Transcription and Translation	T cells;B lineage;Myeloid dendritic cells;Neutrophils;En	B lineage;Neutrophils;Endothelial cells;Fibroblasts
205563_at KISS1	208387_s_at MMP24	0.608861726508785	4	R-HSA-1474228|R-HSA-1592389	Degradation of the extracellular matrix|Activation of Matrix Meta	T cells;B lineage;Myeloid dendritic cells;Neutrophils;En	T cells;CD8 T cells;B lineage;NK cells;Neutrophils;Endothe
226534_at KITLG	205051_s_at KIT	0.706340718105424	4	R-HSA-1433557|R-HSA-199418|R-HSA-	Signaling by SCF-KIT|Negative regulation of the PI3K/AKT netw	Endothelial cells	Neutrophils;Endothelial cells
217512_at KNG1	209869_at ADRA2A	0.523605805958747	4	R-HSA-163685|R-HSA-422356	Integration of energy metabolism|Regulation of insulin secretion	T cells;B lineage;Myeloid dendritic cells;Neutrophils;En	Fibroblasts
243562_at KNG1	207389_at GP1BA	0.517533808523936	4	R-HSA-140837|R-HSA-140877|R-HSA-7	Intrinsic Pathway of Fibrin Clot Formation|Formation of Fibrin C	T cells;Neutrophils;Endothelial cells	Neutrophils;Endothelial cells
243562_at KNG1	205785_at ITGAM	0.576361832226834	4	R-HSA-166016|R-HSA-168898|R-HSA-2	Toll Like Receptor 4 (TLR4) Cascade|Toll-Like Receptors Cascac	T cells;Neutrophils;Endothelial cells	T cells;Neutrophils;Endothelial cells;Fibroblasts
217512_at KNG1	212157_at SDC2	0.501604278074866	4	R-HSA-1630316|R-HSA-1638091|R-HSA	Glycosaminoglycan metabolism|Heparan sulfate/heparin (HS-GA	T cells;B lineage;Myeloid dendritic cells;Neutrophils;En	Endothelial cells;Fibroblasts
204585_s_at L1CAM	211607_x_at EGFR	0.544385026737968	4	R-HSA-1227986|R-HSA-1236382|R-HSA	Signaling by ERBB2|Constitutive Signaling by Ligand-Responsiv	NA	T cells;B lineage;NK cells;Myeloid dendritic cells;Neutrophi
222346_at LAMA1	215878_at ITGB1	0.725494692315219	4	R-HSA-202733|R-HSA-373755|R-HSA-3	Cell surface interactions at the vascular wall|Semaphorin interacti	T cells;B lineage;Neutrophils;Endothelial cells;Fibroblas	T cells
222346_at LAMA1	212157_at SDC2	0.530634071810542	4	R-HSA-1630316|R-HSA-1638091|R-HSA	Glycosaminoglycan metabolism|Heparan sulfate/heparin (HS-GA	T cells;B lineage;Neutrophils;Endothelial cells;Fibroblas	Endothelial cells;Fibroblasts
222346_at LAMA1	232941_at TMPRSS6	0.533384262796027	4	R-HSA-1474228	Degradation of the extracellular matrix	T cells;B lineage;Neutrophils;Endothelial cells;Fibroblas	T cells;B lineage;Myeloid dendritic cells;Neutrophils;Endoth
216839_at LAMA2	216190_x_at ITGB1	0.573109243697479	4	R-HSA-1500931|R-HSA-202733|R-HSA-	Cell-Cell communication|Cell surface interactions at the vascular	NA	T cells;Neutrophils;Endothelial cells;Fibroblasts
1568879_a_at LAMA3	216190_x_at ITGB1	0.717035905271199	4	R-HSA-1500931|R-HSA-202733|R-HSA-	Cell-Cell communication|Cell surface interactions at the vascular	T cells;B lineage;Myeloid dendritic cells;Neutrophils;En	T cells;Neutrophils;Endothelial cells;Fibroblasts
203726_s_at LAMA3	230704_s_at ITGB4	0.661318666457513	4	R-HSA-1474290|R-HSA-1500931|R-HSA	Collagen formation|Cell-Cell communication|Assembly of collage	T cells;Neutrophils;Endothelial cells;Fibroblasts	T cells;B lineage;Myeloid dendritic cells;Neutrophils;Endoth
210089_s_at LAMA4	201474_s_at ITGA3	0.658670741023682	4	R-HSA-202733|R-HSA-6806834|R-HSA-	Cell surface interactions at the vascular wall|Signaling by MET|M	B lineage;Neutrophils;Endothelial cells;Fibroblasts	T cells;Neutrophils;Endothelial cells
210089_s_at LAMA4	215879_at ITGB1	0.699923605805959	4	R-HSA-1500931|R-HSA-202733|R-HSA-	Cell-Cell communication|Cell surface interactions at the vascular	B lineage;Neutrophils;Endothelial cells;Fibroblasts	NK cells;Neutrophils;Endothelial cells
229570_at LAMA5	229643_at ITGA6	0.541940412528648	4	R-HSA-1474290|R-HSA-2022090|R-HSA	Collagen formation|Assembly of collagen fibrils and other multim	T cells;B lineage;Myeloid dendritic cells;Neutrophils;En	Fibroblasts
229570_at LAMA5	216190_x_at ITGB1	0.600916730328495	4	R-HSA-1500931|R-HSA-202733|R-HSA-	Cell-Cell communication|Cell surface interactions at the vascular	T cells;B lineage;Myeloid dendritic cells;Neutrophils;En	T cells;Neutrophils;Endothelial cells;Fibroblasts
229570_at LAMA5	230704_s_at ITGB4	0.738024297363629	4	R-HSA-1474290|R-HSA-1500931|R-HSA	Collagen formation|Cell-Cell communication|Assembly of collage	T cells;B lineage;Myeloid dendritic cells;Neutrophils;En	T cells;B lineage;Myeloid dendritic cells;Neutrophils;Endoth
216264_s_at LAMB2	204990_s_at ITGB4	0.525744843391902	4	R-HSA-1474290	Collagen formation	Monocytic lineage	Neutrophils;Endothelial cells
200771_at LAMC1	1553678_a_at ITGB1	0.506187929717341	4	R-HSA-1500931|R-HSA-202733|R-HSA-	Cell-Cell communication|Cell surface interactions at the vascular	NA	NA
202267_at LAMC2	201474_s_at ITGA3	0.506493506493506	4	R-HSA-202733|R-HSA-6806834|R-HSA-	Cell surface interactions at the vascular wall|Signaling by MET|M	T cells;B lineage;Myeloid dendritic cells;Neutrophils;En	T cells;Neutrophils;Endothelial cells
202267_at LAMC2	216190_x_at ITGB1	0.717341482047364	4	R-HSA-1500931|R-HSA-202733|R-HSA-	Cell-Cell communication|Cell surface interactions at the vascular	T cells;B lineage;Myeloid dendritic cells;Neutrophils;En	T cells;Neutrophils;Endothelial cells;Fibroblasts
202267_at LAMC2	230704_s_at ITGB4	0.663610667381002	4	R-HSA-1474290|R-HSA-1500931|R-HSA	Collagen formation|Cell-Cell communication|Assembly of collage	T cells;B lineage;Myeloid dendritic cells;Neutrophils;En	T cells;B lineage;Myeloid dendritic cells;Neutrophils;Endoth
232558_at LAMC3	216190_x_at ITGB1	0.515355233002292	4	R-HSA-1500931|R-HSA-202733|R-HSA-	Cell-Cell communication|Cell surface interactions at the vascular	T cells;B lineage;Myeloid dendritic cells;Neutrophils;En	T cells;Neutrophils;Endothelial cells;Fibroblasts
232558_at LAMC3	230704_s_at ITGB4	0.579876233642851	4	R-HSA-1474290|R-HSA-1500931|R-HSA	Collagen formation|Cell-Cell communication|Assembly of collage	T cells;B lineage;Myeloid dendritic cells;Neutrophils;En	T cells;B lineage;Myeloid dendritic cells;Neutrophils;Endoth
207584_at LPA	205785_at ITGAM	0.53422983508837	4	R-HSA-166016|R-HSA-168898|R-HSA-2	Toll Like Receptor 4 (TLR4) Cascade|Toll-Like Receptors Cascac	T cells;B lineage;Myeloid dendritic cells;Neutrophils;En	T cells;Neutrophils;Endothelial cells;Fibroblasts
203548_s_at LPL	205710_at LRP2	0.561191749427044	4	R-HSA-196854|R-HSA-2187338|R-HSA-	Metabolism of vitamins and cofactors|Visual phototransduction|M	Endothelial cells	Neutrophils;Endothelial cells
203549_s_at LPL	209822_s_at VLDLR	0.583498854087089	4	R-HSA-174824|R-HSA-8866376	Plasma lipoprotein assembly, remodeling, and clearance|Reelin si	Endothelial cells	Neutrophils;Endothelial cells;Fibroblasts
242705_x_at LRPAP1	212807_s_at SORT1	0.657754010695187	4	R-HSA-199992|R-HSA-421837|R-HSA-4	trans-Golgi Network Vesicle Budding|Clathrin derived vesicle bu	NA	Monocytic lineage;Neutrophils;Endothelial cells
242705_x_at LRPAP1	209822_s_at VLDLR	0.593582887700535	4	R-HSA-174824|R-HSA-8866376	Plasma lipoprotein assembly, remodeling, and clearance|Reelin si	NA	Neutrophils;Endothelial cells;Fibroblasts
202729_s_at LTBP1	201125_s_at ITGB5	0.731398013750955	4	R-HSA-445355	Smooth Muscle Contraction	NA	Endothelial cells
219922_s_at LTBP3	201125_s_at ITGB5	0.618029029793736	4	R-HSA-445355	Smooth Muscle Contraction	Monocytic lineage	Endothelial cells
1559754_at LTB	232819_s_at LTBR	0.659587471352177	4	R-HSA-5668541	TNFR2 non-canonical NF-kB pathway	T cells;B lineage;Myeloid dendritic cells;Neutrophils;En	T cells;B lineage;NK cells;Myeloid dendritic cells;Neutrophi
202018_s_at LTF	200785_s_at LRP1	0.583804430863254	4	R-HSA-196854|R-HSA-2187338|R-HSA-	Metabolism of vitamins and cofactors|Visual phototransduction|M	NA	NA
201744_s_at LUM	211945_s_at ITGB1	0.505271199388846	4	R-HSA-1500931|R-HSA-202733|R-HSA-	Cell-Cell communication|Cell surface interactions at the vascular	Fibroblasts	NA
224620_at MAPK1	211398_at FGFR2	0.683728036669213	4	R-HSA-1226099|R-HSA-1839126|R-HSA	Signaling by FGFR in disease|FGFR2 mutant receptor activation|/	T cells;B lineage;Neutrophils;Endothelial cells;Fibroblas	T cells;B lineage;Myeloid dendritic cells;Neutrophils;Endoth
206904_at MATN1	215879_at ITGB1	0.594194041252865	4	R-HSA-1500931|R-HSA-202733|R-HSA-	Cell-Cell communication|Cell surface interactions at the vascular	T cells;Myeloid dendritic cells;Neutrophils;Endothelial c	NK cells;Neutrophils;Endothelial cells
206904_at MATN1	242493_at PTPRD	0.585026737967914	4	R-HSA-6794362|R-HSA-8849932	Protein-protein interactions at synapses|Synaptic adhesion-like m	T cells;Myeloid dendritic cells;Neutrophils;Endothelial c	Endothelial cells
210605_s_at MFGE8	204627_s_at ITGB3	0.564247517188694	4	R-HSA-194138|R-HSA-202733|R-HSA-3	Signaling by VEGF|Cell surface interactions at the vascular wall|I	NA	NA
213783_at MFNG	223508_at NOTCH1	0.516577540106952	4	R-HSA-157118|R-HSA-3781865|R-HSA-	Signaling by NOTCH|Diseases of glycosylation|Transcriptional re	T cells;B lineage;NK cells;Myeloid dendritic cells;Neutr	NA
213783_at MFNG	210756_s_at NOTCH2	0.581054239877769	4	R-HSA-157118|R-HSA-3781865	Signaling by NOTCH|Diseases of glycosylation	T cells;B lineage;NK cells;Myeloid dendritic cells;Neutr	T cells;Monocytic lineage;Myeloid dendritic cells;Neutrophi
206560_s_at MIA	1554700_at CDH7	0.501909854851031	4	R-HSA-1500931	Cell-Cell communication	Neutrophils;Endothelial cells	B lineage;Neutrophils;Endothelial cells
205959_at MMP13	1569042_at LRP1	0.502521008403361	4	R-HSA-196854|R-HSA-2168880|R-HSA-	Metabolism of vitamins and cofactors|Scavenging of heme from p	T cells;Endothelial cells;Fibroblasts	T cells;B lineage;Myeloid dendritic cells;Neutrophils;Endoth
203936_s_at MMP9	229221_at CD44	0.59449961802903	4	R-HSA-1474228	Degradation of the extracellular matrix	Fibroblasts	B lineage
210155_at MYOC	224337_s_at FZD4	0.544385026737968	4	R-HSA-373080|R-HSA-3858494|R-HSA-	Class B/2 (Secretin family receptors)|Beta-catenin independent W	T cells;B lineage;Myeloid dendritic cells;Neutrophils;En	Endothelial cells;Fibroblasts
231532_at NCAM1	208020_s_at CACNA1C	0.587974636905848	4	R-HSA-163685|R-HSA-375165|R-HSA-4	Integration of energy metabolism|NCAM signaling for neurite out	Endothelial cells	T cells;B lineage;Myeloid dendritic cells;Neutrophils;Endoth
212843_at NCAM1	203638_s_at FGFR2	0.936440030557678	4	R-HSA-1226099|R-HSA-5655253|R-HSA	Signaling by FGFR in disease|Signaling by FGFR2 in disease|Sig	Neutrophils;Endothelial cells	Neutrophils;Endothelial cells;Fibroblasts
231532_at NCAM1	205696_s_at GFRA1	0.636669213139801	4	R-HSA-8853659	RET signaling	Endothelial cells	NA
212843_at NCAM1	213194_at ROBO1	0.803514132925898	4	R-HSA-373752	Netrin-1 signaling	Neutrophils;Endothelial cells	Neutrophils;Endothelial cells
205143_at NCAN	203440_at CDH2	0.542857142857143	4	R-HSA-375170|R-HSA-381426|R-HSA-5	CDO in myogenesis|Regulation of Insulin-like Growth Factor (IG	Neutrophils;Endothelial cells	Endothelial cells;Fibroblasts
206814_at NGF	214932_at KIDINS220	0.522383498854087	4	R-HSA-166520|R-HSA-187037	Signaling by NTRKs|Signaling by NTRK1 (TRKA)	T cells;B lineage;Myeloid dendritic cells;Neutrophils;En	T cells;B lineage;Neutrophils;Endothelial cells;Fibroblasts
206814_at NGF	212797_at SORT1	0.527883880825057	4	R-HSA-199992|R-HSA-421837|R-HSA-4	trans-Golgi Network Vesicle Budding|Clathrin derived vesicle bu	T cells;B lineage;Myeloid dendritic cells;Neutrophils;En	T cells;Neutrophils;Endothelial cells
1561082_at NID1	216190_x_at ITGB1	0.663559969442322	4	R-HSA-1500931|R-HSA-202733|R-HSA-	Cell-Cell communication|Cell surface interactions at the vascular	T cells;B lineage;Neutrophils;Endothelial cells	T cells;Neutrophils;Endothelial cells;Fibroblasts
1561082_at NID1	204625_s_at ITGB3	0.531856378915202	4	R-HSA-194138|R-HSA-202733|R-HSA-3	Signaling by VEGF|Cell surface interactions at the vascular wall|I	T cells;B lineage;Neutrophils;Endothelial cells	T cells;B lineage;Neutrophils;Fibroblasts
204114_at NID2	211343_s_at COL13A1	0.548663101604278	4	R-HSA-1474290|R-HSA-1650814	Collagen formation|Collagen biosynthesis and modifying enzyme	NA	NA
205893_at NLGN1	228547_at NRXN1	0.577692895339954	4	R-HSA-6794362	Protein-protein interactions at synapses	Endothelial cells	NA
205893_at NLGN1	209983_s_at NRXN2	0.634530175706646	4	R-HSA-6794362	Protein-protein interactions at synapses	Endothelial cells	Endothelial cells
205893_at NLGN1	229649_at NRXN3	0.732009167303285	4	R-HSA-6794362	Protein-protein interactions at synapses	Endothelial cells	Endothelial cells
235838_at NLGN2	216096_s_at NRXN1	0.557219251336898	4	R-HSA-6794362	Protein-protein interactions at synapses	B lineage;Neutrophils;Endothelial cells;Fibroblasts	T cells;B lineage;Myeloid dendritic cells;Neutrophils;Endoth
226288_s_at NLGN2	209982_s_at NRXN2	0.697173414820474	4	R-HSA-6794362	Protein-protein interactions at synapses	Neutrophils;Endothelial cells	Neutrophils;Endothelial cells
226288_s_at NLGN2	205795_at NRXN3	0.60977845683728	4	R-HSA-6794362	Protein-protein interactions at synapses	Neutrophils;Endothelial cells	Endothelial cells
234751_s_at NLGN3	216096_s_at NRXN1	0.664782276546982	4	R-HSA-6794362	Protein-protein interactions at synapses	T cells;B lineage;NK cells;Myeloid dendritic cells;Neutr	T cells;B lineage;Myeloid dendritic cells;Neutrophils;Endoth
219726_at NLGN3	209983_s_at NRXN2	0.616501145912911	4	R-HSA-6794362	Protein-protein interactions at synapses	Neutrophils;Endothelial cells	Endothelial cells

219726_at NLGN3	229649_at NRXN3	0.550496562261268	4	R-HSA-6794362	Protein-protein interactions at synapses	Neutrophils;Endothelial cells	Endothelial cells
220689_at NODAL	208222_at ACVR1B	0.54656582022147	4	R-HSA-1181150|R-HSA-9006936	Signaling by NODAL|Signaling by TGF-beta family members	NA	T cells;B lineage;NK cells;Neutrophils;Endothelial cells;Fibr
204501_at NOV	1558140_at PLXNA1	0.60305576776165	4	R-HSA-373755|R-HSA-399954|R-HSA-3	Semaphorin interactions|Sema3A PAK dependent Axon repulsion	T cells;B lineage;NK cells;Monocytic lineage;Myeloid d	T cells;B lineage;Myeloid dendritic cells;Neutrophils;Endoth
1553663_a_at NPB	209869_at ADRA2A	0.574025974025974	4	R-HSA-163685|R-HSA-422356	Integration of energy metabolism|Regulation of insulin secretion	T cells;Neutrophils;Endothelial cells	Fibroblasts
244747_at NPNT	215879_at ITGB1	0.638502673796791	4	R-HSA-1500931|R-HSA-202733|R-HSA-	Cell-Cell communication|Cell surface interactions at the vascular	Fibroblasts	NK cells;Neutrophils;Endothelial cells
208241_at NRG1	234354_x_at ERBB2	0.626890756302521	4	R-HSA-1227986|R-HSA-1250196|R-HSA	Signaling by ERBB2|SHC1 events in ERBB2 signaling|PI3K eve	T cells;B lineage;NK cells;Myeloid dendritic cells;Neutr	T cells;Myeloid dendritic cells;Neutrophils;Endothelial cells;
208062_s_at NRG2	234354_x_at ERBB2	0.72620320855615	4	R-HSA-1227986|R-HSA-1250196|R-HSA	Signaling by ERBB2|SHC1 events in ERBB2 signaling|PI3K eve	T cells;B lineage;Myeloid dendritic cells;Neutrophils;En	T cells;Myeloid dendritic cells;Neutrophils;Endothelial cells;
242426_at NRG4	211551_at EGFR	0.566386554621849	4	R-HSA-1227986|R-HSA-1236394|R-HSA	Signaling by ERBB2|Signaling by ERBB4|SHC1 events in ERBE	NA	T cells;B lineage;Myeloid dendritic cells;Neutrophils;Endoth
242426_at NRG4	234354_x_at ERBB2	0.537662337662338	4	R-HSA-1227986|R-HSA-1250196|R-HSA	Signaling by ERBB2|SHC1 events in ERBB2 signaling|PI3K eve	NA	T cells;Myeloid dendritic cells;Neutrophils;Endothelial cells;
237847_at NTN1	242653_at DCC	0.722230710466005	4	R-HSA-109581|R-HSA-373752|R-HSA-3	Apoptosis|Netrin-1 signaling|DSCAM interactions|DCC mediated	T cells;B lineage;NK cells;Myeloid dendritic cells;Neutr	T cells;NK cells;Myeloid dendritic cells;Neutrophils;Endoth
208005_at NTN1	229877_at NEO1	0.531245225362872	4	R-HSA-373752|R-HSA-375170|R-HSA-5	Netrin-1 signaling|CDO in myogenesis|Myogenesis	T cells;Neutrophils;Endothelial cells;Fibroblasts	T cells;B lineage;Myeloid dendritic cells;Neutrophils;Endoth
208005_at NTN1	243833_at UNC5A	0.525439266615737	4	R-HSA-109581|R-HSA-373752|R-HSA-5	Apoptosis|Netrin-1 signaling|Programmed Cell Death	T cells;Neutrophils;Endothelial cells;Fibroblasts	T cells;B lineage;Myeloid dendritic cells;Neutrophils;Endoth
227816_at NTN1	41856_at UNC5B	0.547746371275783	4	R-HSA-109581|R-HSA-5357801	Apoptosis|Programmed Cell Death	Endothelial cells	Neutrophils;Endothelial cells
203675_at NUCB2	214012_at ERAP1	0.648227383863081	4	R-HSA-983170	Antigen Presentation: Folding, assembly and peptide loading of c	NA	NA
232377_at NXPH1	228547_at NRXN1	0.544996180290298	4	R-HSA-6794362	Protein-protein interactions at synapses	T cells;Neutrophils;Endothelial cells	NA
232377_at NXPH1	209983_s_at NRXN2	0.574025974025974	4	R-HSA-6794362	Protein-protein interactions at synapses	T cells;Neutrophils;Endothelial cells	Endothelial cells
232377_at NXPH1	229649_at NRXN3	0.581970970206264	4	R-HSA-6794362	Protein-protein interactions at synapses	T cells;Neutrophils;Endothelial cells	Endothelial cells
230883_at NXPH2	209915_s_at NRXN1	0.728953399541635	4	R-HSA-6794362	Protein-protein interactions at synapses	Neutrophils;Endothelial cells	NA
221991_at NXPH3	216096_s_at NRXN1	0.577692895339954	4	R-HSA-6794362	Protein-protein interactions at synapses	T cells;NK cells;Myeloid dendritic cells;Neutrophils;Enc	T cells;B lineage;Myeloid dendritic cells;Neutrophils;Endoth
228210_at NXPH3	215020_at NRXN3	0.645225362872422	4	R-HSA-6794362	Protein-protein interactions at synapses	Endothelial cells	Endothelial cells
234841_x_at OBP2A	221375_at OR1G1	0.659179465595589	4	R-HSA-381753	Olfactory Signaling Pathway	NA	NA
207576_x_at OXT	206251_s_at AVPR1A	0.643086325439267	4	R-HSA-5619115	Disorders of transmembrane transporters	T cells;B lineage;Myeloid dendritic cells;Neutrophils;En	T cells;B lineage;Neutrophils;Endothelial cells;Fibroblasts
207576_x_at OXT	208111_at AVPR2	0.515660809778457	4	R-HSA-432040|R-HSA-445717|R-HSA-5	Vasopressin regulates renal water homeostasis via Aquaporins|Aq	T cells;B lineage;Myeloid dendritic cells;Neutrophils;En	Endothelial cells;Fibroblasts
233896_s_at PAPLN	202895_s_at SIRPA	0.571581359816654	4	R-HSA-1500931|R-HSA-202733	Cell-Cell communication|Cell surface interactions at the vascular	NA	T cells;Neutrophils;Endothelial cells;Fibroblasts
227759_at PCSK9	217173_s_at LDLR	0.662948815889992	4	R-HSA-174824|R-HSA-196854|R-HSA-2	Plasma lipoprotein assembly, remodeling, and clearance|Metaboli	B lineage;Neutrophils;Endothelial cells;Fibroblasts	NA
205463_s_at PDGFA	203131_at PDGFRA	0.634224598930481	4	R-HSA-199418|R-HSA-2219528|R-HSA-	Negative regulation of the PI3K/AKT network|PI3K/AKT Signali	Endothelial cells	NA
216061_x_at PDGFB	200784_s_at LRP1	0.625362872421696	4	R-HSA-196854|R-HSA-2187338|R-HSA-	Metabolism of vitamins and cofactors|Visual phototransduction|M	T cells;NK cells;Neutrophils;Endothelial cells	T cells;Endothelial cells
216061_x_at PDGFB	1554828_at PDGFRA	0.566014676535276	4	R-HSA-186763|R-HSA-186797|R-HSA-1	Downstream signal transduction|Signaling by PDGF|Negative reg	T cells;NK cells;Neutrophils;Endothelial cells	B lineage;NK cells;Neutrophils;Endothelial cells
222719_s_at PDGFC	204406_at FLT1	0.501909854851031	4	R-HSA-194138	Signaling by VEGF	NA	T cells;NK cells;Myeloid dendritic cells;Neutrophils;Endoth
218718_at PDGFC	203131_at PDGFRA	0.615278838808251	4	R-HSA-199418|R-HSA-2219528|R-HSA-	Negative regulation of the PI3K/AKT network|PI3K/AKT Signali	T cells;Endothelial cells	NA
210938_at PDX1	206535_at SLC2A2	0.541940412528648	4	R-HSA-163685|R-HSA-422356	Integration of energy metabolism|Regulation of insulin secretion	T cells;B lineage;Myeloid dendritic cells;Neutrophils;En	NA
237774_at PENK	207792_at OPRD1	0.61038961038961	4	R-HSA-6785807	Interleukin-4 and 13 signaling	T cells;B lineage;Myeloid dendritic cells;Neutrophils;En	Neutrophils;Endothelial cells;Fibroblasts
237774_at PENK	207994_s_at OPRM1	0.810236822001528	4	R-HSA-111885|R-HSA-6785807	Opioid Signalling|Interleukin-4 and 13 signaling	T cells;B lineage;Myeloid dendritic cells;Neutrophils;En	T cells;B lineage;Myeloid dendritic cells;Neutrophils;Endoth
209652_s_at PGF	204406_at FLT1	0.561497326203208	4	R-HSA-194138	Signaling by VEGF	Endothelial cells;Fibroblasts	T cells;NK cells;Myeloid dendritic cells;Neutrophils;Endoth
209652_s_at PGF	210841_s_at NRP2	0.500076394194041	4	R-HSA-194138|R-HSA-373760	Signaling by VEGF|L1CAM interactions	Endothelial cells;Fibroblasts	T cells;CD8 T cells;B lineage;Myeloid dendritic cells;Neutro
1559638_at PIK3CD-AS1	207114_at LY6G6C	0.571886936592819	4	R-HSA-163125	Post-translational modification: synthesis of GPI-anchored protei	NA	NA
201251_at PKM	1557905_s_at CD44	0.762566844919786	4	R-HSA-1474228	Degradation of the extracellular matrix	B lineage	B lineage
205479_s_at PLAU	205786_s_at ITGAM	0.718869365928189	4	R-HSA-166016|R-HSA-168898|R-HSA-2	Toll Like Receptor 4 (TLR4) Cascade|Toll-Like Receptors Cascac	Monocytic lineage	Monocytic lineage
205479_s_at PLAU	1555349_a_at ITGB2	0.682200152788388	4	R-HSA-166016|R-HSA-168898|R-HSA-2	Toll Like Receptor 4 (TLR4) Cascade|Toll-Like Receptors Cascac	Monocytic lineage	Monocytic lineage
240033_at PLG	204406_at FLT1	0.697173414820474	4	R-HSA-194138	Signaling by VEGF	T cells;B lineage;NK cells;Myeloid dendritic cells;Neutr	T cells;NK cells;Myeloid dendritic cells;Neutrophils;Endoth
240033_at PLG	1555336_a_at ITGA9	0.683116883116883	4	R-HSA-373760	L1CAM interactions	T cells;B lineage;NK cells;Myeloid dendritic cells;Neutr	CD8 T cells;Endothelial cells;Fibroblasts
230931_at PLG	205785_at ITGAM	0.638808250572956	4	R-HSA-166016|R-HSA-168898|R-HSA-2	Toll Like Receptor 4 (TLR4) Cascade|Toll-Like Receptors Cascac	T cells;B lineage;Myeloid dendritic cells;Neutrophils;En	T cells;Neutrophils;Endothelial cells;Fibroblasts
240033_at PLG	215879_at ITGB1	0.705423987776929	4	R-HSA-1500931|R-HSA-202733|R-HSA-	Cell-Cell communication|Cell surface interactions at the vascular	T cells;B lineage;NK cells;Myeloid dendritic cells;Neutr	NK cells;Neutrophils;Endothelial cells
202075_s_at PLTP	203504_s_at ABCA1	0.59022154316272	4	R-HSA-1989781|R-HSA-400206	PPARA activates gene expression|Regulation of lipid metabolism	Monocytic lineage	Monocytic lineage
205720_at POMC	207792_at OPRD1	0.54503781960581	4	R-HSA-6785807	Interleukin-4 and 13 signaling	T cells;B lineage;NK cells;Myeloid dendritic cells;Neutr	Neutrophils;Endothelial cells;Fibroblasts
205720_at POMC	207994_s_at OPRM1	0.751776302904566	4	R-HSA-111885|R-HSA-6785807	Opioid Signalling|Interleukin-4 and 13 signaling	T cells;B lineage;NK cells;Myeloid dendritic cells;Neutr	T cells;B lineage;Myeloid dendritic cells;Neutrophils;Endoth
205445_at PRL	210476_s_at PRLR	0.574942704354469	4	R-HSA-1170546|R-HSA-982772	Prolactin receptor signaling|Growth hormone receptor signaling	T cells;Myeloid dendritic cells;Neutrophils;Endothelial c	NA
207808_s_at PROS1	202686_s_at AXL	0.695034377387319	4	R-HSA-194138|R-HSA-4420097	Signaling by VEGF|VEGFA-VEGFR2 Pathway	Monocytic lineage;Neutrophils;Endothelial cells	T cells;Monocytic lineage;Endothelial cells
200871_s_at PSAP	200785_s_at LRP1	0.757677616501146	4	R-HSA-196854|R-HSA-2187338|R-HSA-	Metabolism of vitamins and cofactors|Visual phototransduction|M	Monocytic lineage	NA
200871_s_at PSAP	212807_s_at SORT1	0.677922077922078	4	R-HSA-199992|R-HSA-421837|R-HSA-4	trans-Golgi Network Vesicle Budding|Clathrin derived vesicle bu	Monocytic lineage	Monocytic lineage;Neutrophils;Endothelial cells
207782_s_at PSEN1	208759_at NCSTN	0.536745607333843	4	R-HSA-157118|R-HSA-2682334|R-HSA-	Signaling by NOTCH|EPH-Ephrin signaling|EPH-ephrin mediate	Endothelial cells	NA
226577_at PSEN1	210756_s_at NOTCH2	0.507715813598167	4	R-HSA-157118|R-HSA-3781865	Signaling by NOTCH|Diseases of glycosylation	Endothelial cells	T cells;Monocytic lineage;Myeloid dendritic cells;Neutrophi
221373_x_at PSPN	221199_at GFRA4	0.611917494270435	4	R-HSA-8853659	RET signaling	NA	NA
1555238_at PTH2	207151_at ADCYAP1R1	0.601527883880825	4	R-HSA-166520|R-HSA-187037|R-HSA-3	Signaling by NTRKs|Signaling by NTRK1 (TRKA)|Class B/2 (Se	T cells;B lineage;NK cells;Myeloid dendritic cells;Neutr	T cells
1555238_at PTH2	205019_s_at VIPR1	0.619862490450726	4	R-HSA-373080|R-HSA-420092	Class B/2 (Secretin family receptors)|Glucagon-type ligand recept	T cells;B lineage;NK cells;Myeloid dendritic cells;Neutr	NA
206977_at PTH	207151_at ADCYAP1R1	0.637585943468296	4	R-HSA-166520|R-HSA-187037|R-HSA-3	Signaling by NTRKs|Signaling by NTRK1 (TRKA)|Class B/2 (Se	T cells;NK cells;Endothelial cells	T cells
209465_x_at PTN	226571_s_at PTPRS	0.664171122994652	4	R-HSA-6794362	Protein-protein interactions at synapses	Neutrophils;Endothelial cells;Fibroblasts	Neutrophils;Endothelial cells
211737_x_at PTN	202898_at SDC3	0.600916730328495	4	R-HSA-196854|R-HSA-202733|R-HSA-2	Metabolism of vitamins and cofactors|Cell surface interactions at	Neutrophils;Endothelial cells;Fibroblasts	T cells;Monocytic lineage
205923_at RELN	209822_s_at VLDLR	0.537356760886173	4	R-HSA-174824|R-HSA-8866376	Plasma lipoprotein assembly, remodeling, and clearance|Reelin si	Neutrophils;Endothelial cells	Neutrophils;Endothelial cells;Fibroblasts
234406_at RGMA	1556075_at BMPR2	0.545912910618793	4	R-HSA-201451|R-HSA-9006936	Signaling by BMP|Signaling by TGF-beta family members	NA	T cells;B lineage;Myeloid dendritic cells;Neutrophils;Endoth
223468_s_at RGMA	204321_at NEO1	0.574331550802139	4	R-HSA-373752|R-HSA-375170|R-HSA-5	Netrin-1 signaling|CDO in myogenesis|Myogenesis	NA	Neutrophils;Endothelial cells
216184_s_at RIMS1	208020_s_at CACNA1C	0.598670641215466	4	R-HSA-163685|R-HSA-375165|R-HSA-4	Integration of energy metabolism|NCAM signaling for neurite out	T cells;B lineage;Neutrophils;Endothelial cells;Fibroblas	T cells;B lineage;Myeloid dendritic cells;Neutrophils;Endoth
231986_at RIMS1	204230_s_at SLC17A7	0.564858670741024	4	R-HSA-112310|R-HSA-210500	Neurotransmitter release cycle|Glutamate Neurotransmitter Releas	NA	NA
216184_s_at RIMS1	205857_at SLC18A2	0.585943468296409	4	R-HSA-112310	Neurotransmitter release cycle	T cells;B lineage;Neutrophils;Endothelial cells;Fibroblas	NK cells;Endothelial cells;Fibroblasts
216184_s_at RIMS1	207150_at SLC18A3	0.581054239877769	4	R-HSA-112310|R-HSA-8856825|R-HSA-	Neurotransmitter release cycle|Cargo recognition for clathrin-med	T cells;B lineage;Neutrophils;Endothelial cells;Fibroblas	T cells;B lineage;NK cells;Myeloid dendritic cells;Neutrophi
205230_at RPH3A	209915_s_at NRXN1	0.607028265851795	4	R-HSA-6794362	Protein-protein interactions at synapses	NA	NA
244624_at RPS27A	234354_x_at ERBB2	0.511993888464477	4	R-HSA-1227986|R-HSA-1250196|R-HSA	Signaling by ERBB2|SHC1 events in ERBB2 signaling|PI3K eve	T cells;B lineage;Neutrophils;Endothelial cells;Fibroblas	T cells;Myeloid dendritic cells;Neutrophils;Endothelial cells;

244624_at RPS27A	211398_at FGFR2	0.697478991596639	4	R-HSA-1226099|R-HSA-1839126|R-HSA	Signaling by FGFR in disease|FGFR2 mutant receptor activation|	T cells;B lineage;Neutrophils;Endothelial cells;Fibroblas	T cells;B lineage;Myeloid dendritic cells;Neutrophils;Endoth
244624_at RPS27A	217103_at LDLR	0.592360580595875	4	R-HSA-174824|R-HSA-196854|R-HSA-2	Plasma lipoprotein assembly, remodeling, and clearance|Metaboli	T cells;B lineage;Neutrophils;Endothelial cells;Fibroblas	T cells;B lineage;NK cells;Myeloid dendritic cells;Neutrophi
244624_at RPS27A	207334_s_at TGFBR2	0.533384262796027	4	R-HSA-170834|R-HSA-2173789|R-HSA-	Signaling by TGF-beta Receptor Complex|TGF-beta receptor sign	T cells;B lineage;Neutrophils;Endothelial cells;Fibroblas	B lineage;Neutrophils;Endothelial cells;Fibroblasts
203535_at S100A9	224341_x_at TLR4	0.532467532467532	4	R-HSA-109581|R-HSA-166016|R-HSA-1	Apoptosis|Toll Like Receptor 4 (TLR4) Cascade|MyD88:Mal casc	Monocytic lineage	Monocytic lineage
209879_at SELPLG	205786_s_at ITGAM	0.667532467532468	4	R-HSA-166016|R-HSA-168898|R-HSA-2	Toll Like Receptor 4 (TLR4) Cascade|Toll-Like Receptors Cascac	Monocytic lineage	Monocytic lineage
209879_at SELPLG	202803_s_at ITGB2	0.574025974025974	4	R-HSA-166016|R-HSA-168898|R-HSA-2	Toll Like Receptor 4 (TLR4) Cascade|Toll-Like Receptors Cascac	Monocytic lineage	Monocytic lineage
244849_at SEMA3A	210615_at NRP1	0.72864782276547	4	R-HSA-194138|R-HSA-373755|R-HSA-3	Signaling by VEGF|Semaphorin interactions|L1CAM interactions	NA	T cells;B lineage;Myeloid dendritic cells;Neutrophils;Endoth
244163_at SEMA3A	210841_s_at NRP2	0.638808250572956	4	R-HSA-194138|R-HSA-373760	Signaling by VEGF|L1CAM interactions	T cells;B lineage;NK cells;Myeloid dendritic cells;Neutr	T cells;CD8 T cells;B lineage;Myeloid dendritic cells;Neutro
244163_at SEMA3A	1558140_at PLXNA1	0.623223834988541	4	R-HSA-373755|R-HSA-399954|R-HSA-3	Semaphorin interactions|Sema3A PAK dependent Axon repulsion	T cells;B lineage;NK cells;Myeloid dendritic cells;Neutr	T cells;B lineage;Myeloid dendritic cells;Neutrophils;Endoth
244849_at SEMA3A	227032_at PLXNA2	0.570359052711994	4	R-HSA-373755	Semaphorin interactions	NA	Endothelial cells
244163_at SEMA3A	1553139_s_at PLXNA3	0.6640690675657	4	R-HSA-373755|R-HSA-399954|R-HSA-3	Semaphorin interactions|Sema3A PAK dependent Axon repulsion	T cells;B lineage;NK cells;Myeloid dendritic cells;Neutr	T cells;B lineage;Myeloid dendritic cells;Neutrophils;Endoth
244163_at SEMA3A	234859_at PLXNA4	0.524828113063407	4	R-HSA-373755|R-HSA-399954|R-HSA-3	Semaphorin interactions|Sema3A PAK dependent Axon repulsion	T cells;B lineage;NK cells;Myeloid dendritic cells;Neutr	Endothelial cells
203788_s_at SEMA3C	210510_s_at NRP1	0.563941940412529	4	R-HSA-194138|R-HSA-373755	Signaling by VEGF|Semaphorin interactions	NA	Endothelial cells
203789_s_at SEMA3C	222877_at NRP2	0.649809014514897	4	R-HSA-194138|R-HSA-373760	Signaling by VEGF|L1CAM interactions	Endothelial cells	Monocytic lineage
215324_at SEMA3D	210615_at NRP1	0.713063407181054	4	R-HSA-194138|R-HSA-373755|R-HSA-3	Signaling by VEGF|Semaphorin interactions|L1CAM interactions	Neutrophils;Endothelial cells;Fibroblasts	T cells;B lineage;Myeloid dendritic cells;Neutrophils;Endoth
209730_at SEMA3F	210615_at NRP1	0.711841100076394	4	R-HSA-194138|R-HSA-373755|R-HSA-3	Signaling by VEGF|Semaphorin interactions|L1CAM interactions	T cells;B lineage;Myeloid dendritic cells;Neutrophils;En	T cells;B lineage;Myeloid dendritic cells;Neutrophils;Endoth
209730_at SEMA3F	228102_at NRP2	0.619251336898396	4	R-HSA-194138|R-HSA-373760	Signaling by VEGF|L1CAM interactions	T cells;B lineage;Myeloid dendritic cells;Neutrophils;En	B lineage;Neutrophils;Endothelial cells;Fibroblasts
209730_at SEMA3F	1558140_at PLXNA1	0.673032849503438	4	R-HSA-373755|R-HSA-399954|R-HSA-3	Semaphorin interactions|Sema3A PAK dependent Axon repulsion	T cells;B lineage;Myeloid dendritic cells;Neutrophils;En	T cells;B lineage;Myeloid dendritic cells;Neutrophils;Endoth
209730_at SEMA3F	1567519_at PLXNA3	0.712146676852559	4	R-HSA-373755|R-HSA-399954|R-HSA-3	Semaphorin interactions|Sema3A PAK dependent Axon repulsion	T cells;B lineage;Myeloid dendritic cells;Neutrophils;En	T cells;B lineage;Myeloid dendritic cells;Neutrophils;Endoth
228891_at SEMA4D	215807_s_at PLXNB1	0.737509549274255	4	R-HSA-373755|R-HSA-416482	Semaphorin interactions|G alpha (12/13) signalling events	Neutrophils;Endothelial cells	Neutrophils;Endothelial cells
208124_s_at SEMA4F	228102_at NRP2	0.554163483575248	4	R-HSA-194138|R-HSA-373760	Signaling by VEGF|L1CAM interactions	T cells;B lineage;NK cells;Myeloid dendritic cells;Neutr	B lineage;Neutrophils;Endothelial cells;Fibroblasts
220454_s_at SEMA6A	207290_at PLXNA2	0.538314616896908	4	R-HSA-373755|R-HSA-399954	Semaphorin interactions|Sema3A PAK dependent Axon repulsion	T cells;B lineage;Neutrophils;Endothelial cells	T cells;Endothelial cells
220454_s_at SEMA6A	234859_at PLXNA4	0.571930630441421	4	R-HSA-373755|R-HSA-399954|R-HSA-3	Semaphorin interactions|Sema3A PAK dependent Axon repulsion	T cells;B lineage;Neutrophils;Endothelial cells	Endothelial cells
244746_at SEMA6D	1558140_at PLXNA1	0.747899159663866	4	R-HSA-373755|R-HSA-399954|R-HSA-3	Semaphorin interactions|Sema3A PAK dependent Axon repulsion	T cells;B lineage;Myeloid dendritic cells;Neutrophils;En	T cells;B lineage;Myeloid dendritic cells;Neutrophils;Endoth
226492_at SEMA6D	219725_at TREM2	0.59327731092437	4	R-HSA-2172127|R-HSA-2424491|R-HSA	DAP12 interactions|DAP12 signaling|Semaphorin interactions	Neutrophils;Endothelial cells;Fibroblasts	NA
210083_at SEMA7A	216190_x_at ITGB1	0.689228418640183	4	R-HSA-1500931|R-HSA-202733|R-HSA-	Cell-Cell communication|Cell surface interactions at the vascular	T cells;B lineage;NK cells;Myeloid dendritic cells;Neutr	T cells;Neutrophils;Endothelial cells;Fibroblasts
210083_at SEMA7A	206471_s_at PLXNC1	0.649503437738732	4	R-HSA-373755	Semaphorin interactions	T cells;B lineage;NK cells;Myeloid dendritic cells;Neutr	T cells;Myeloid dendritic cells;Neutrophils;Endothelial cells;
211429_s_at SERPINA1	200785_s_at LRP1	0.675783040488923	4	R-HSA-196854|R-HSA-2187338|R-HSA-	Metabolism of vitamins and cofactors|Visual phototransduction|M	Monocytic lineage	NA
210049_at SERPINC1	202755_s_at GPC1	0.508021390374331	4	R-HSA-1630316|R-HSA-1638091|R-HSA	Glycosaminoglycan metabolism|Heparan sulfate/heparin (HS-GA	Endothelial cells;Fibroblasts	Endothelial cells
210049_at SERPINC1	1569042_at LRP1	0.559663865546218	4	R-HSA-196854|R-HSA-2168880|R-HSA-	Metabolism of vitamins and cofactors|Scavenging of heme from p	Endothelial cells;Fibroblasts	T cells;B lineage;Myeloid dendritic cells;Neutrophils;Endoth
227487_s_at SERPINE2	1555353_at LRP1	0.610283445894479	4	R-HSA-196854|R-HSA-2168880|R-HSA-	Metabolism of vitamins and cofactors|Scavenging of heme from p	Endothelial cells	B lineage;NK cells;Neutrophils;Endothelial cells;Fibroblasts
200986_at SERPING1	200785_s_at LRP1	0.606417112299465	4	R-HSA-196854|R-HSA-2187338|R-HSA-	Metabolism of vitamins and cofactors|Visual phototransduction|M	T cells;Cytotoxic lymphocytes;Monocytic lineage	NA
230130_at SLIT2	213194_at ROBO1	0.588082505729565	4	R-HSA-373752	Netrin-1 signaling	Endothelial cells	Neutrophils;Endothelial cells
203021_at SLPI	203547_at CD4	0.522077922077922	4	R-HSA-162587|R-HSA-162909|R-HSA-2	HIV Life Cycle|Host Interactions of HIV factors|TCR signaling|C	NA	Monocytic lineage
218087_s_at SORBS1	213792_s_at INSR	0.693506493506493	4	R-HSA-199418|R-HSA-6811558|R-HSA-	Negative regulation of the PI3K/AKT network|PI5P, PP2A and IE	Neutrophils;Endothelial cells;Fibroblasts	Neutrophils;Endothelial cells
222513_s_at SORBS1	201124_at ITGB5	0.693812070282658	4	R-HSA-445355	Smooth Muscle Contraction	Neutrophils;Endothelial cells;Fibroblasts	Neutrophils;Endothelial cells
223869_at SOST	205606_at LRP6	0.538273491214668	4	R-HSA-4641262|R-HSA-4791275	Disassembly of the destruction complex and recruitment of AXIN	T cells;B lineage;Neutrophils;Endothelial cells;Fibroblas	T cells;Myeloid dendritic cells;Neutrophils;Endothelial cells
235546_at SPINT1	216906_at ST14	0.628418640183346	4	R-HSA-6805567|R-HSA-6809371	Keratinization|Formation of the cornified envelope	T cells;Neutrophils;Endothelial cells;Fibroblasts	T cells;Myeloid dendritic cells;Endothelial cells;Fibroblasts
209875_s_at SPP1	202351_at ITGAV	0.800152788388082	4	R-HSA-194138|R-HSA-202733|R-HSA-2	Signaling by VEGF|Cell surface interactions at the vascular wall|I	Neutrophils	Neutrophils;Endothelial cells
1568574_x_at SPP1	214020_x_at ITGB5	0.585332314744079	4	R-HSA-445355	Smooth Muscle Contraction	NA	NA
1556864_at TCTN1	1552765_x_at TMEM67	0.5181450087702	4	R-HSA-5620912	Anchoring of the basal body to the plasma membrane	Neutrophils	Neutrophils
214378_at TFPI	1569042_at LRP1	0.532773109243697	4	R-HSA-196854|R-HSA-2168880|R-HSA-	Metabolism of vitamins and cofactors|Scavenging of heme from p	Endothelial cells	T cells;B lineage;Myeloid dendritic cells;Neutrophils;Endoth
214064_at TF	230863_at LRP2	0.911910767425701	4	R-HSA-196854|R-HSA-2187338|R-HSA-	Metabolism of vitamins and cofactors|Visual phototransduction|M	Neutrophils;Endothelial cells	Neutrophils;Endothelial cells;Fibroblasts
211258_s_at TGFA	211607_x_at EGFR	0.802902979373568	4	R-HSA-1227986|R-HSA-1236382|R-HSA	Signaling by ERBB2|Constitutive Signaling by Ligand-Responsiv	T cells;B lineage;NK cells;Myeloid dendritic cells;Neutr	T cells;B lineage;NK cells;Myeloid dendritic cells;Neutrophi
211258_s_at TGFA	234354_x_at ERBB2	0.622918258212376	4	R-HSA-1227986|R-HSA-1250196|R-HSA	Signaling by ERBB2|SHC1 events in ERBB2 signaling|PI3K even	T cells;B lineage;NK cells;Myeloid dendritic cells;Neutr	T cells;Myeloid dendritic cells;Neutrophils;Endothelial cells;
203084_at TGFB1	215878_at ITGB1	0.597295440661372	4	R-HSA-202733|R-HSA-373755|R-HSA-3	Cell surface interactions at the vascular wall|Semaphorin interacti	B lineage;Endothelial cells;Fibroblasts	T cells
203084_at TGFB1	211579_at ITGB3	0.690503478216613	4	R-HSA-194138|R-HSA-202733|R-HSA-2	Signaling by VEGF|Cell surface interactions at the vascular wall|I	B lineage;Endothelial cells;Fibroblasts	T cells;B lineage;NK cells;Myeloid dendritic cells;Neutrophi
203084_at TGFB1	205396_at SMAD3	0.566997708174179	4	R-HSA-446652|R-HSA-5689880|R-HSA-	Interleukin-1 family signaling|Ub-specific processing proteases|T	B lineage;Endothelial cells;Fibroblasts	Endothelial cells
220406_at TGFB2	207334_s_at TGFBR2	0.535523300229183	4	R-HSA-170834|R-HSA-2173789|R-HSA-	Signaling by TGF-beta Receptor Complex|TGF-beta receptor sign	T cells;B lineage;Neutrophils;Endothelial cells;Fibroblas	B lineage;Neutrophils;Endothelial cells;Fibroblasts
1555540_at TGFB3	215879_at ITGB1	0.702979373567609	4	R-HSA-1500931|R-HSA-202733|R-HSA-	Cell-Cell communication|Cell surface interactions at the vascular	T cells;B lineage;NK cells;Myeloid dendritic cells;Neutr	NK cells;Neutrophils;Endothelial cells
1555540_at TGFB3	211579_at ITGB3	0.691267478524442	4	R-HSA-194138|R-HSA-202733|R-HSA-2	Signaling by VEGF|Cell surface interactions at the vascular wall|I	T cells;B lineage;NK cells;Myeloid dendritic cells;Neutr	T cells;B lineage;NK cells;Myeloid dendritic cells;Neutrophi
209747_at TGFB3	201124_at ITGB5	0.72559205500382	4	R-HSA-445355	Smooth Muscle Contraction	Monocytic lineage;Neutrophils;Endothelial cells	Neutrophils;Endothelial cells
1555540_at TGFB3	207334_s_at TGFBR2	0.651642475171887	4	R-HSA-170834|R-HSA-2173789|R-HSA-	Signaling by TGF-beta Receptor Complex|TGF-beta receptor sign	T cells;B lineage;NK cells;Myeloid dendritic cells;Neutr	B lineage;Neutrophils;Endothelial cells;Fibroblasts
216183_at TGM2	1555336_a_at ITGA9	0.567303284950344	4	R-HSA-373760	L1CAM interactions	T cells;B lineage;NK cells;Neutrophils;Endothelial cells	CD8 T cells;Endothelial cells;Fibroblasts
216183_at TGM2	216190_x_at ITGB1	0.588999236058059	4	R-HSA-1500931|R-HSA-202733|R-HSA-	Cell-Cell communication|Cell surface interactions at the vascular	T cells;B lineage;NK cells;Neutrophils;Endothelial cells	T cells;Neutrophils;Endothelial cells;Fibroblasts
216183_at TGM2	204626_s_at ITGB3	0.625974025974026	4	R-HSA-194138|R-HSA-202733|R-HSA-3	Signaling by VEGF|Cell surface interactions at the vascular wall|I	T cells;B lineage;NK cells;Neutrophils;Endothelial cells	T cells;Myeloid dendritic cells;Neutrophils;Endothelial cells;
216183_at TGM2	207554_x_at TBXA2R	0.617112299465241	4	R-HSA-416482	G alpha (12/13) signalling events	T cells;B lineage;NK cells;Neutrophils;Endothelial cells	T cells;B lineage;Myeloid dendritic cells;Neutrophils;Endoth
201107_s_at THBS1	209554_at CD36	0.533689839572193	4	R-HSA-114608|R-HSA-166016|R-HSA-1	Platelet degranulation|Toll Like Receptor 4 (TLR4) Cascade|MyD	T cells;Endothelial cells;Fibroblasts	T cells;B lineage;NK cells;Myeloid dendritic cells;Neutrophi
201107_s_at THBS1	216956_s_at ITGA2B	0.637224946315362	4	R-HSA-114608|R-HSA-354192|R-HSA-3	Platelet degranulation|Integrin alphaIIb beta3 signaling|L1CAM in	T cells;Endothelial cells;Fibroblasts	T cells;B lineage;Myeloid dendritic cells;Neutrophils;Endoth
239336_at THBS1	201474_s_at ITGA3	0.547746371275783	4	R-HSA-202733|R-HSA-6806834|R-HSA-	Cell surface interactions at the vascular wall|Signaling by MET|M	Neutrophils;Endothelial cells	T cells;Neutrophils;Endothelial cells
215775_at THBS1	216190_x_at ITGB1	0.600916730328495	4	R-HSA-1500931|R-HSA-202733|R-HSA-	Cell-Cell communication|Cell surface interactions at the vascular	B lineage;Neutrophils;Endothelial cells	T cells;Neutrophils;Endothelial cells;Fibroblasts
215775_at THBS1	204626_s_at ITGB3	0.686783804430863	4	R-HSA-194138|R-HSA-202733|R-HSA-3	Signaling by VEGF|Cell surface interactions at the vascular wall|I	B lineage;Neutrophils;Endothelial cells	T cells;Myeloid dendritic cells;Neutrophils;Endothelial cells;
215775_at THBS1	1569042_at LRP1	0.649197860962567	4	R-HSA-196854|R-HSA-2168880|R-HSA-	Metabolism of vitamins and cofactors|Scavenging of heme from p	B lineage;Neutrophils;Endothelial cells	T cells;B lineage;Myeloid dendritic cells;Neutrophils;Endoth
201107_s_at THBS1	209468_at LRP5	0.573720397249809	4	R-HSA-4641262|R-HSA-4791275	Disassembly of the destruction complex and recruitment of AXIN	T cells;Endothelial cells;Fibroblasts	NA
211154_at THPO	217240_at SIRPB1	0.747288006111536	4	R-HSA-1500931|R-HSA-2172127	Cell-Cell communication|DAP12 interactions	T cells;B lineage;NK cells;Neutrophils;Endothelial cells;	T cells;CD8 T cells;B lineage;NK cells;Myeloid dendritic ce
232763_at TLN1	204628_s_at ITGB3	0.571275783040489	4	R-HSA-194138|R-HSA-202733|R-HSA-3	Signaling by VEGF|Cell surface interactions at the vascular wall|I	T cells;B lineage;Myeloid dendritic cells;Neutrophils;En	Neutrophils
216005_at TNC	227314_at ITGA2	0.588585837152112	4	R-HSA-373760|R-HSA-6806834|R-HSA-	L1CAM interactions|Signaling by MET|MET promotes cell motil	NA	Neutrophils

216005_at TNC	202351_at ITGAV	0.709297885789227	4	R-HSA-194138|R-HSA-202733|R-HSA-2	Signaling by VEGF|Cell surface interactions at the vascular wall|I	NA	Neutrophils;Endothelial cells
201645_at TNC	211945_s_at ITGB1	0.573414820473644	4	R-HSA-1500931|R-HSA-202733|R-HSA-	Cell-Cell communication|Cell surface interactions at the vascular	Monocytic lineage	NA
214329_x_at TNFSF10	209295_at TNFRSF10B	0.661420932009167	4	R-HSA-109581|R-HSA-202733|R-HSA-5	Apoptosis|Cell surface interactions at the vascular wall|Programm	NA	Neutrophils
210314_x_at TNFSF13	212158_at SDC2	0.565775401069519	4	R-HSA-1630316|R-HSA-1638091|R-HSA	Glycosaminoglycan metabolism|Heparan sulfate/heparin (HS-GA	T cells;Monocytic lineage	Endothelial cells
210314_x_at TNFSF13	207643_s_at TNFRSF1A	0.639419404125286	4	R-HSA-5668541|R-HSA-6783783|R-HSA	TNFR2 non-canonical NF-kB pathway|Interleukin-10 signaling|D	T cells;Monocytic lineage	Monocytic lineage
206907_at TNFSF9	204413_at TRAF2	0.586554621848739	4	R-HSA-109581|R-HSA-168928|R-HSA-5	Apoptosis|DDX58/IFIH1-mediated induction of interferon-alpha/	NA	Fibroblasts
208980_s_at UBC	221060_s_at TLR4	0.503437738731856	4	R-HSA-109581|R-HSA-5357801	Apoptosis|Programmed Cell Death	NA	NA
232674_at UCN2	208593_x_at CRHR1	0.520589809755255	4	R-HSA-373080	Class B/2 (Secretin family receptors)	NA	Endothelial cells;Fibroblasts
1552676_at UCN3	211897_s_at CRHR1	0.532355414495835	4	R-HSA-373080	Class B/2 (Secretin family receptors)	T cells;B lineage;NK cells;Myeloid dendritic cells;Neutr	T cells;B lineage;Neutrophils;Endothelial cells;Fibroblasts
1552676_at UCN3	211510_s_at CRHR2	0.699923605805959	4	R-HSA-2980736	Peptide hormone metabolism	T cells;B lineage;NK cells;Myeloid dendritic cells;Neutr	T cells;B lineage;Myeloid dendritic cells;Neutrophils;Endoth
203868_s_at VCAM1	211945_s_at ITGB1	0.617723453017571	4	R-HSA-1500931|R-HSA-202733|R-HSA-	Cell-Cell communication|Cell surface interactions at the vascular	T cells;Cytotoxic lymphocytes;Monocytic lineage	NA
203868_s_at VCAM1	202803_s_at ITGB2	0.506493506493506	4	R-HSA-166016|R-HSA-168898|R-HSA-2	Toll Like Receptor 4 (TLR4) Cascade|Toll-Like Receptors Cascac	T cells;Cytotoxic lymphocytes;Monocytic lineage	Monocytic lineage
211571_s_at VCAN	234418_x_at CD44	0.500687547746371	4	R-HSA-1474228|R-HSA-202733	Degradation of the extracellular matrix|Cell surface interactions a	Endothelial cells;Fibroblasts	T cells;B lineage;NK cells;Myeloid dendritic cells;Neutrophi
211571_s_at VCAN	1565483_at EGFR	0.552330022918258	4	R-HSA-1227986|R-HSA-1236382|R-HSA	Signaling by ERBB2|Constitutive Signaling by Ligand-Responsiv	Endothelial cells;Fibroblasts	T cells;B lineage;Myeloid dendritic cells;Neutrophils;Endoth
204619_s_at VCAN	211945_s_at ITGB1	0.710007639419404	4	R-HSA-1500931|R-HSA-202733|R-HSA-	Cell-Cell communication|Cell surface interactions at the vascular	Neutrophils	NA
204619_s_at VCAN	210176_at TLR1	0.571581359816654	4	R-HSA-166016|R-HSA-166058|R-HSA-1	Toll Like Receptor 4 (TLR4) Cascade|MyD88:Mal cascade initiat	Neutrophils	Monocytic lineage
221731_x_at VCAN	204924_at TLR2	0.666921313980138	4	R-HSA-166016|R-HSA-166058|R-HSA-1	Toll Like Receptor 4 (TLR4) Cascade|MyD88:Mal cascade initiat	NA	Monocytic lineage
210513_s_at VEGFA	210651_s_at EPHB2	0.561802902979374	4	R-HSA-2682334|R-HSA-373760|R-HSA-	EPH-Ephrin signaling|L1CAM interactions|EPHB-mediated forw	NA	T cells;B lineage;Myeloid dendritic cells;Neutrophils;Endoth
212171_x_at VEGFA	222033_s_at FLT1	0.620779220779221	4	R-HSA-194138	Signaling by VEGF	NA	NA
210512_s_at VEGFA	202351_at ITGAV	0.515966386554622	4	R-HSA-194138|R-HSA-202733|R-HSA-2	Signaling by VEGF|Cell surface interactions at the vascular wall|I	NA	Neutrophils;Endothelial cells
210512_s_at VEGFA	211945_s_at ITGB1	0.596638655462185	4	R-HSA-1500931|R-HSA-202733|R-HSA-	Cell-Cell communication|Cell surface interactions at the vascular	NA	NA
212171_x_at VEGFA	210510_s_at NRP1	0.527883880825057	4	R-HSA-194138|R-HSA-373755	Signaling by VEGF|Semaphorin interactions	NA	Endothelial cells
212171_x_at VEGFA	214632_at NRP2	0.565469824293354	4	R-HSA-194138	Signaling by VEGF	NA	Neutrophils
210513_s_at VEGFA	202895_s_at SIRPA	0.525744843391902	4	R-HSA-1500931|R-HSA-202733	Cell-Cell communication|Cell surface interactions at the vascular	NA	T cells;Neutrophils;Endothelial cells;Fibroblasts
209946_at VEGFC	1555336_a_at ITGA9	0.519938884644767	4	R-HSA-373760	L1CAM interactions	Fibroblasts	CD8 T cells;Endothelial cells;Fibroblasts
206577_at VIP	205019_s_at VIPR1	0.695034377387319	4	R-HSA-373080|R-HSA-420092	Class B/2 (Secretin family receptors)|Glucagon-type ligand recept	Endothelial cells	NA
233980_s_at VWF	207389_at GP1BA	0.633613445378151	4	R-HSA-140837|R-HSA-140877|R-HSA-7	Intrinsic Pathway of Fibrin Clot Formation|Formation of Fibrin C	T cells;B lineage;Neutrophils;Endothelial cells;Fibroblas	Neutrophils;Endothelial cells
233980_s_at VWF	216956_s_at ITGA2B	0.603147928802574	4	R-HSA-114608|R-HSA-354192|R-HSA-3	Platelet degranulation|Integrin alphaIIb beta3 signaling|L1CAM i	T cells;B lineage;Neutrophils;Endothelial cells;Fibroblas	T cells;B lineage;Myeloid dendritic cells;Neutrophils;Endoth
233980_s_at VWF	204626_s_at ITGB3	0.592360580595875	4	R-HSA-194138|R-HSA-202733|R-HSA-3	Signaling by VEGF|Cell surface interactions at the vascular wall|I	T cells;B lineage;Neutrophils;Endothelial cells;Fibroblas	T cells;Myeloid dendritic cells;Neutrophils;Endothelial cells;
233980_s_at VWF	202895_s_at SIRPA	0.556302521008403	4	R-HSA-1500931|R-HSA-202733	Cell-Cell communication|Cell surface interactions at the vascular	T cells;B lineage;Neutrophils;Endothelial cells;Fibroblas	T cells;Neutrophils;Endothelial cells;Fibroblasts
205648_at WNT2	204452_s_at FZD1	0.60799144497099	4	R-HSA-3858494|R-HSA-4086400|R-HSA	Beta-catenin independent WNT signaling|PCP/CE pathway|Disas	T cells;B lineage;NK cells;Myeloid dendritic cells;Neutr	Endothelial cells
205648_at WNT2	206136_at FZD5	0.532467532467532	4	R-HSA-373080|R-HSA-3858494|R-HSA-	Class B/2 (Secretin family receptors)|Beta-catenin independent W	T cells;B lineage;NK cells;Myeloid dendritic cells;Neutr	T cells;Endothelial cells
205648_at WNT2	207639_at FZD9	0.661726508785332	4	R-HSA-373080	Class B/2 (Secretin family receptors)	T cells;B lineage;NK cells;Myeloid dendritic cells;Neutr	T cells;Endothelial cells
205648_at WNT2	205606_at LRP6	0.686478227654698	4	R-HSA-4641262|R-HSA-4791275	Disassembly of the destruction complex and recruitment of AXIN	T cells;B lineage;NK cells;Myeloid dendritic cells;Neutr	T cells;Myeloid dendritic cells;Neutrophils;Endothelial cells
230751_at WNT4	238129_s_at FZD2	0.791902215431627	4	R-HSA-373080|R-HSA-3858494|R-HSA-	Class B/2 (Secretin family receptors)|Beta-catenin independent W	T cells;B lineage;Myeloid dendritic cells;Neutrophils;En	T cells;B lineage;Myeloid dendritic cells;Neutrophils;Endoth
238105_x_at WNT7B	204452_s_at FZD1	0.644663459746822	4	R-HSA-3858494|R-HSA-4086400|R-HSA	Beta-catenin independent WNT signaling|PCP/CE pathway|Disas	T cells;B lineage;NK cells;Myeloid dendritic cells;Neutr	Endothelial cells
238105_x_at WNT7B	219764_at FZD10	0.577692895339954	4	R-HSA-373080	Class B/2 (Secretin family receptors)	T cells;B lineage;NK cells;Myeloid dendritic cells;Neutr	Neutrophils;Endothelial cells
238105_x_at WNT7B	229591_at LRP5	0.603972498090145	4	R-HSA-4641262|R-HSA-4791275	Disassembly of the destruction complex and recruitment of AXIN	T cells;B lineage;NK cells;Myeloid dendritic cells;Neutr	Neutrophils;Endothelial cells
207612_at WNT8B	229591_at LRP5	0.616195569136746	4	R-HSA-4641262|R-HSA-4791275	Disassembly of the destruction complex and recruitment of AXIN	T cells;B lineage;Myeloid dendritic cells;Neutrophils;En	Neutrophils;Endothelial cells
202535_at FADD	204781_s_at FAS	0.619862490450726	4	R-HSA-109581|R-HSA-5357801	Apoptosis|Programmed Cell Death	B lineage	B lineage
235600_at DLK1	223508_at NOTCH1	0.505271199388846	4	R-HSA-157118|R-HSA-3781865|R-HSA-	Signaling by NOTCH|Diseases of glycosylation|Transcriptional re	B lineage;Myeloid dendritic cells;Neutrophils;Endotheli	NA
222898_s_at DLL3	223508_at NOTCH1	0.556913674560733	4	R-HSA-157118|R-HSA-3781865|R-HSA-	Signaling by NOTCH|Diseases of glycosylation|Transcriptional re	T cells;B lineage;NK cells;Myeloid dendritic cells;Neutr	NA
229755_x_at DLL3	210756_s_at NOTCH2	0.555080213903743	4	R-HSA-157118|R-HSA-3781865	Signaling by NOTCH|Diseases of glycosylation	T cells;B lineage;Myeloid dendritic cells;Neutrophils;En	T cells;Monocytic lineage;Myeloid dendritic cells;Neutrophi
223525_at DLL4	210756_s_at NOTCH2	0.527160212402592	4	R-HSA-157118|R-HSA-3781865	Signaling by NOTCH|Diseases of glycosylation	T cells;B lineage;NK cells;Myeloid dendritic cells;Neutr	T cells;Monocytic lineage;Myeloid dendritic cells;Neutrophi
235600_at DLK1	240786_at NOTCH4	0.557830404889228	4	R-HSA-157118|R-HSA-1912408|R-HSA-	Signaling by NOTCH|Pre-NOTCH Transcription and Translation	B lineage;Myeloid dendritic cells;Neutrophils;Endotheli	B lineage;Neutrophils;Endothelial cells;Fibroblasts
222898_s_at DLL3	240786_at NOTCH4	0.629946524064171	4	R-HSA-157118|R-HSA-1912408|R-HSA-	Signaling by NOTCH|Pre-NOTCH Transcription and Translation	T cells;B lineage;NK cells;Myeloid dendritic cells;Neutr	B lineage;Neutrophils;Endothelial cells;Fibroblasts
231033_at DSC2	1553105_s_at DSG2	0.575859434682964	4	R-HSA-109581|R-HSA-111465|R-HSA-3	Apoptosis|Apoptotic cleavage of cellular proteins|Apoptotic cleav	Myeloid dendritic cells;Neutrophils	T cells;NK cells;Neutrophils;Endothelial cells
207324_s_at DSC1	206642_at DSG1	0.57182151589242	4	R-HSA-109581|R-HSA-111465|R-HSA-3	Apoptosis|Apoptotic cleavage of cellular proteins|Apoptotic cleav	T cells;Myeloid dendritic cells;Neutrophils;Endothelial c	B lineage;NK cells;Neutrophils;Endothelial cells
207324_s_at DSC1	1553105_s_at DSG2	0.57422263136491	4	R-HSA-109581|R-HSA-111465|R-HSA-3	Apoptosis|Apoptotic cleavage of cellular proteins|Apoptotic cleav	T cells;Myeloid dendritic cells;Neutrophils;Endothelial c	T cells;NK cells;Neutrophils;Endothelial cells
206033_s_at DSC3	206642_at DSG1	0.591641838383431	4	R-HSA-109581|R-HSA-111465|R-HSA-3	Apoptosis|Apoptotic cleavage of cellular proteins|Apoptotic cleav	T cells;B lineage;NK cells;Myeloid dendritic cells;Neutr	B lineage;NK cells;Neutrophils;Endothelial cells
206032_at DSC3	1553105_s_at DSG2	0.615278838808251	4	R-HSA-109581|R-HSA-111465|R-HSA-3	Apoptosis|Apoptotic cleavage of cellular proteins|Apoptotic cleav	T cells;B lineage;NK cells;Myeloid dendritic cells;Neutr	T cells;NK cells;Neutrophils;Endothelial cells
215485_s_at ICAM1	216980_s_at SPN	0.798319327731092	4	R-HSA-202733	Cell surface interactions at the vascular wall	T cells;B lineage;NK cells;Myeloid dendritic cells;Neutr	T cells;B lineage;NK cells;Myeloid dendritic cells;Neutrophi
211657_at CEACAM6	210610_at CEACAM1	0.500993124522536	4	R-HSA-202733	Cell surface interactions at the vascular wall	T cells;B lineage;Myeloid dendritic cells;Neutrophils;En	NA
216268_s_at JAG1	208783_s_at CD46	0.589304812834225	4	R-HSA-166658|R-HSA-977606	Complement cascade|Regulation of Complement cascade	Endothelial cells;Fibroblasts	NA
206760_s_at FCER2	244097_at CR2	0.59144385026738	4	R-HSA-166658|R-HSA-977606	Complement cascade|Regulation of Complement cascade	T cells;Myeloid dendritic cells;Neutrophils;Endothelial c	B lineage;Myeloid dendritic cells;Neutrophils;Endothelial ce
203757_s_at CEACAM6	206676_at CEACAM8	0.663610667381002	4	R-HSA-202733	Cell surface interactions at the vascular wall	NA	NA
219669_at CD177	1559921_at PECAM1	0.722841864018335	4	R-HSA-202733|R-HSA-418346|R-HSA-4	Cell surface interactions at the vascular wall|Platelet homeostasis|	Myeloid dendritic cells;Neutrophils	T cells;B lineage;Myeloid dendritic cells;Neutrophils;Endoth
238105_x_at WNT7B	224337_s_at FZD4	0.72864782276547	4	R-HSA-373080|R-HSA-3858494|R-HSA-	Class B/2 (Secretin family receptors)|Beta-catenin independent W	T cells;B lineage;NK cells;Myeloid dendritic cells;Neutr	Endothelial cells;Fibroblasts
205746_s_at ADAM17	206794_at ERBB4	0.542551566080978	4	GO:0001934|GO:0007169|GO:0007507|G	positive regulation of protein phosphorylation|transmembrane rec	T cells;B lineage;NK cells;Myeloid dendritic cells;Neutr	Endothelial cells
206281_at ADCYAP1	217303_s_at ADRB3	0.541023682200153	4	GO:0007188|GO:0006898	adenylate cyclase-modulating G-protein coupled receptor signalin	T cells;B lineage;Neutrophils;Endothelial cells;Fibroblas	T cells;Cytotoxic lymphocytes
215564_at AREG	215638_at ERBB3	0.713368983957219	4	GO:0007169|GO:0007507|GO:0009968|G	transmembrane receptor protein tyrosine kinase signaling pathwa	T cells;B lineage;Myeloid dendritic cells;Neutrophils;En	T cells;B lineage;Myeloid dendritic cells;Neutrophils;Endoth
207848_at AVP	217303_s_at ADRB3	0.753093964858671	4	GO:0007188|GO:0006898	adenylate cyclase-modulating G-protein coupled receptor signalin	T cells;B lineage;Myeloid dendritic cells;Neutrophils;En	T cells;Cytotoxic lymphocytes
207848_at AVP	208260_at AVPR1B	0.74392666157372	4	GO:0007204	positive regulation of cytosolic calcium ion concentration	T cells;B lineage;Myeloid dendritic cells;Neutrophils;En	T cells;NK cells;Myeloid dendritic cells;Neutrophils;Endoth
216231_s_at B2M	210031_at CD247	0.508326967150497	4	GO:0002250|GO:0031295|GO:0038096|G	adaptive immune response|T cell costimulation|Fc-gamma recept	T cells;B lineage;Myeloid dendritic cells;Neutrophils;En	T cells;B lineage;Myeloid dendritic cells;Neutrophils;Endoth
241412_at BTC	233498_at ERBB4	0.514438502673797	4	GO:0001934|GO:0007169|GO:0014068|G	positive regulation of protein phosphorylation|transmembrane rec	T cells;B lineage;Neutrophils;Endothelial cells;Fibroblas	Endothelial cells
217561_at CALCA	217303_s_at ADRB3	0.617112299465241	4	GO:0007188|GO:0006898	adenylate cyclase-modulating G-protein coupled receptor signalin	Fibroblasts	T cells;Cytotoxic lymphocytes
210133_at CCL11	217119_s_at CXCR3	0.603361344537815	4	GO:0007204	positive regulation of cytosolic calcium ion concentration	Endothelial cells	B lineage;Neutrophils;Endothelial cells;Fibroblasts

216714_at CCL13	208304_at CCR3	0.699923605805959	4	GO:0007188|GO:0007204	adenylate cyclase-modulating G-protein coupled receptor signalin	Fibroblasts	T cells;Myeloid dendritic cells;Endothelial cells
206407_s_at CCL13	217119_s_at CXCR3	0.60916730328495	4	GO:0007204	positive regulation of cytosolic calcium ion concentration	T cells;Neutrophils;Endothelial cells	B lineage;Neutrophils;Endothelial cells;Fibroblasts
207354_at CCL16	221169_s_at HRH4	0.681588999236058	4	GO:0007204	positive regulation of cytosolic calcium ion concentration	Neutrophils;Endothelial cells	T cells;B lineage;Myeloid dendritic cells;Neutrophils;Endoth
221463_at CCL24	208304_at CCR3	0.573414820473644	4	GO:0007188|GO:0007204	adenylate cyclase-modulating G-protein coupled receptor signalin	T cells;B lineage;Myeloid dendritic cells;Neutrophils;En	T cells;Myeloid dendritic cells;Endothelial cells
223710_at CCL26	208304_at CCR3	0.788235294117647	4	GO:0007188|GO:0007204	adenylate cyclase-modulating G-protein coupled receptor signalin	Endothelial cells	T cells;Myeloid dendritic cells;Endothelial cells
201130_s_at CDH1	215638_at ERBB3	0.671199388846448	4	GO:0007169|GO:0007507|GO:0009968|G	transmembrane receptor protein tyrosine kinase signaling pathway	T cells;B lineage;NK cells;Neutrophils;Endothelial cells;	T cells;B lineage;Myeloid dendritic cells;Neutrophils;Endoth
233615_at CGA	217303_s_at ADRB3	0.524216959511077	4	GO:0007188|GO:0006898	adenylate cyclase-modulating G-protein coupled receptor signalin	B lineage;Endothelial cells;Fibroblasts	T cells;Cytotoxic lymphocytes
202312_s_at COL1A1	222899_at ITGA11	0.561497326203208	4	GO:0007160|GO:0007229|GO:0030198	cell-matrix adhesion|integrin-mediated signaling pathway|extracel	T cells;B lineage;Myeloid dendritic cells;Neutrophils;En	T cells;B lineage;Neutrophils;Endothelial cells;Fibroblasts
214641_at COL4A3	213055_at CD47	0.500993124522536	4	GO:0050900	leukocyte migration	T cells;Neutrophils;Endothelial cells	Neutrophils;Endothelial cells;Fibroblasts
205629_s_at CRH	217303_s_at ADRB3	0.633919022154316	4	GO:0007188|GO:0006898	adenylate cyclase-modulating G-protein coupled receptor signalin	T cells	T cells;Cytotoxic lymphocytes
230101_at CXCL2	221468_at XCR1	0.684033613445378	4	GO:0007204	positive regulation of cytosolic calcium ion concentration	T cells;B lineage;Myeloid dendritic cells;Neutrophils;En	B lineage;Endothelial cells
240556_at DCN	233494_at ERBB4	0.538884644766998	4	GO:0001934|GO:0007169|GO:0007507|G	positive regulation of protein phosphorylation|transmembrane rec	Fibroblasts	T cells;Neutrophils;Endothelial cells
206254_at EGF	233498_at ERBB4	0.562414056531704	4	GO:0001934|GO:0007169|GO:0014068|G	positive regulation of protein phosphorylation|transmembrane rec	T cells;B lineage;Myeloid dendritic cells;Neutrophils;En	Endothelial cells
217254_s_at EPO	209962_at EPOR	0.760733384262796	4	GO:0007507	heart development	B lineage;Neutrophils;Endothelial cells;Fibroblasts	T cells;B lineage;NK cells;Myeloid dendritic cells;Neutrophi
205767_at EREG	215638_at ERBB3	0.610695187165775	4	GO:0007169|GO:0007507|GO:0009968|G	transmembrane receptor protein tyrosine kinase signaling pathway	T cells;Myeloid dendritic cells;Neutrophils;Endothelial c	T cells;B lineage;Myeloid dendritic cells;Neutrophils;Endoth
205767_at EREG	206794_at ERBB4	0.510466004583652	4	GO:0001934|GO:0007169|GO:0007507|G	positive regulation of protein phosphorylation|transmembrane rec	T cells;Myeloid dendritic cells;Neutrophils;Endothelial c	Endothelial cells
203305_at F13A1	227297_at ITGA9	0.646447669977082	4	GO:0030198	extracellular matrix organization	Endothelial cells	Neutrophils;Endothelial cells
205754_at F2	206429_at F2RL1	0.792268319219548	4	GO:0007204|GO:0007596|GO:0050900	positive regulation of cytosolic calcium ion concentration|blood c	Endothelial cells	Monocytic lineage
205754_at F2	206795_at F2RL2	0.624140565317036	4	GO:0007596|GO:0030168	blood coagulation|platelet activation	Endothelial cells	T cells;Neutrophils;Endothelial cells;Fibroblasts
205754_at F2	207221_at F2RL3	0.594194041252865	4	GO:0007596|GO:0030168	blood coagulation|platelet activation	Endothelial cells	T cells;Endothelial cells
208449_s_at FGF8	230842_at FGFR2	0.582887700534759	4	GO:0051897|GO:0090263	positive regulation of protein kinase B signaling|positive regulatic	T cells;B lineage;NK cells;Myeloid dendritic cells;Neutr	T cells;B lineage;Myeloid dendritic cells;Neutrophils;Endoth
214701_s_at FN1	214265_at ITGA8	0.503743315508021	4	GO:0030198	extracellular matrix organization	T cells;B lineage;Neutrophils;Endothelial cells;Fibroblas	T cells
212464_s_at FN1	210845_s_at PLAUR	0.565775401069519	4	GO:0007596	blood coagulation	T cells;Neutrophils;Endothelial cells	T cells;B lineage;Myeloid dendritic cells;Neutrophils;Endoth
206670_s_at GAD1	210234_at GRM4	0.538579067990833	4	GO:0000187	activation of MAPK activity	Endothelial cells	Neutrophils;Endothelial cells
224242_at GALP	33579_i_at GALR3	0.502101002305773	4	GO:0007188	adenylate cyclase-modulating G-protein coupled receptor signalin	Neutrophils;Endothelial cells;Fibroblasts	T cells;B lineage;Myeloid dendritic cells;Neutrophils;Endoth
214240_at GAL	220821_at GALR1	0.535828877005348	4	GO:0007204	positive regulation of cytosolic calcium ion concentration	T cells;B lineage;NK cells;Myeloid dendritic cells;Neutr	T cells;NK cells;Myeloid dendritic cells;Endothelial cells
214240_at GAL	33579_i_at GALR3	0.655614973262032	4	GO:0007188	adenylate cyclase-modulating G-protein coupled receptor signalin	T cells;B lineage;NK cells;Myeloid dendritic cells;Neutr	T cells;B lineage;Myeloid dendritic cells;Neutrophils;Endoth
221359_at GDNF	205722_s_at GFRA2	0.625057295645531	4	GO:0007169	transmembrane receptor protein tyrosine kinase signaling pathway	Endothelial cells;Fibroblasts	T cells;B lineage;NK cells;Myeloid dendritic cells;Neutrophi
214524_at GHRH	217303_s_at ADRB3	0.509549274255157	4	GO:0007188|GO:0006898	adenylate cyclase-modulating G-protein coupled receptor signalin	Endothelial cells;Fibroblasts	T cells;Cytotoxic lymphocytes
207899_at GIP	217303_s_at ADRB3	0.679449961802903	4	GO:0007188|GO:0006898	adenylate cyclase-modulating G-protein coupled receptor signalin	T cells;B lineage;Myeloid dendritic cells;Neutrophils;En	T cells;Cytotoxic lymphocytes
217057_s_at GNAS	217303_s_at ADRB3	0.688006111535523	4	GO:0007188|GO:0006898	adenylate cyclase-modulating G-protein coupled receptor signalin	T cells;B lineage;Myeloid dendritic cells;Neutrophils;En	T cells;Cytotoxic lymphocytes
38037_at HBEGF	214053_at ERBB4	0.504354469060351	4	GO:0001934|GO:0007169|GO:0014068|G	positive regulation of protein phosphorylation|transmembrane rec	T cells;B lineage;NK cells;Myeloid dendritic cells;Neutr	T cells;B lineage;Myeloid dendritic cells;Neutrophils;Endoth
207067_s_at HDC	221170_at HRH4	0.624341051558548	4	GO:0007204	positive regulation of cytosolic calcium ion concentration	Endothelial cells	T cells;B lineage;Myeloid dendritic cells;Neutrophils;Endoth
213932_x_at HLA-A	207697_x_at LILRB2	0.613139801375095	4	GO:0002250|GO:0050776	adaptive immune response|regulation of immune response	Neutrophils;Endothelial cells	T cells;Myeloid dendritic cells;Neutrophils;Endothelial cells;
208812_x_at HLA-C	207697_x_at LILRB2	0.507104660045837	4	GO:0002250|GO:0050776	adaptive immune response|regulation of immune response	T cells;B lineage;NK cells;Neutrophils;Endothelial cells;	T cells;Myeloid dendritic cells;Neutrophils;Endothelial cells;
210514_x_at HLA-G	207697_x_at LILRB2	0.542551566080978	4	GO:0002250|GO:0050776	adaptive immune response|regulation of immune response	Endothelial cells;Fibroblasts	T cells;Myeloid dendritic cells;Neutrophils;Endothelial cells;
207062_at IAPP	217303_s_at ADRB3	0.528495034377387	4	GO:0007188|GO:0006898	adenylate cyclase-modulating G-protein coupled receptor signalin	T cells;B lineage;Myeloid dendritic cells;Neutrophils;En	T cells;Cytotoxic lymphocytes
208261_x_at IFNA10	204785_x_at IFNAR2	0.582276546982429	4	GO:0007259|GO:0060338	JAK-STAT cascade|regulation of type I interferon-mediated signa	T cells;B lineage;NK cells;Myeloid dendritic cells;Neutr	Monocytic lineage;Neutrophils
208182_x_at IFNA14	204785_x_at IFNAR2	0.572498090145149	4	GO:0007259|GO:0060338	JAK-STAT cascade|regulation of type I interferon-mediated signa	T cells;B lineage;NK cells;Myeloid dendritic cells;Neutr	Monocytic lineage;Neutrophils
211405_x_at IFNA17	204785_x_at IFNAR2	0.514172207169485	4	GO:0007259|GO:0060338	JAK-STAT cascade|regulation of type I interferon-mediated signa	Cytotoxic lymphocytes	Monocytic lineage;Neutrophils
214569_at IFNA5	204785_x_at IFNAR2	0.521161191749427	4	GO:0007259|GO:0060338	JAK-STAT cascade|regulation of type I interferon-mediated signa	T cells;B lineage;Myeloid dendritic cells;Neutrophils;En	Monocytic lineage;Neutrophils
210118_s_at IL1A	205227_at IL1RAP	0.511688311688312	4	GO:0065003|GO:0070498	protein-containing complex assembly|interleukin-1-mediated sign	T cells;Neutrophils;Endothelial cells	T cells;NK cells;Neutrophils;Endothelial cells
205067_at IL1B	205227_at IL1RAP	0.537967914438503	4	GO:0065003|GO:0070498	protein-containing complex assembly|interleukin-1-mediated sign	Neutrophils;Fibroblasts	T cells;NK cells;Neutrophils;Endothelial cells
221091_at INSL5	221066_at RXFP3	0.694423223834988	4	GO:0007188	adenylate cyclase-modulating G-protein coupled receptor signalin	Endothelial cells	T cells;Neutrophils;Endothelial cells
211124_s_at KITLG	209962_at EPOR	0.657448433919022	4	GO:0007507	heart development	T cells;B lineage;NK cells;Neutrophils;Endothelial cells;	T cells;B lineage;NK cells;Myeloid dendritic cells;Neutrophi
204582_s_at KLK3	209962_at EPOR	0.747192301057587	4	GO:0007507	heart development	B lineage;Neutrophils;Fibroblasts	T cells;B lineage;NK cells;Myeloid dendritic cells;Neutrophi
227048_at LAMA1	205816_at ITGB8	0.600916730328495	4	GO:0030198	extracellular matrix organization	T cells;Myeloid dendritic cells;Neutrophils;Endothelial c	T cells;B lineage;Myeloid dendritic cells;Neutrophils;Endoth
203236_s_at LGALS9	235458_at HAVCR2	0.616501145912911	4	GO:0002250|GO:0010629	adaptive immune response|negative regulation of gene expression	T cells;B lineage;NK cells;Myeloid dendritic cells;Neutr	T cells;B lineage;NK cells;Myeloid dendritic cells;Neutrophi
208241_at NRG1	215638_at ERBB3	0.735064935064935	4	GO:0007169|GO:0007507|GO:0009968|G	transmembrane receptor protein tyrosine kinase signaling pathway	T cells;B lineage;NK cells;Myeloid dendritic cells;Neutr	T cells;B lineage;Myeloid dendritic cells;Neutrophils;Endoth
208232_x_at NRG1	206794_at ERBB4	0.614973262032086	4	GO:0001934|GO:0007169|GO:0007507|G	positive regulation of protein phosphorylation|transmembrane rec	B lineage;Myeloid dendritic cells;Neutrophils;Endothelia	Endothelial cells
208062_s_at NRG2	1563252_at ERBB3	0.844461420932009	4	GO:0007169|GO:0007507|GO:0009968|G	transmembrane receptor protein tyrosine kinase signaling pathway	T cells;B lineage;Myeloid dendritic cells;Neutrophils;En	B lineage;Myeloid dendritic cells;Neutrophils;Endothelial ce
208062_s_at NRG2	206794_at ERBB4	0.698701298701299	4	GO:0001934|GO:0007169|GO:0007507|G	positive regulation of protein phosphorylation|transmembrane rec	T cells;B lineage;Myeloid dendritic cells;Neutrophils;En	Endothelial cells
229233_at NRG3	202454_s_at ERBB3	0.578304048892284	4	GO:0007169|GO:0009968|GO:0014068|G	transmembrane receptor protein tyrosine kinase signaling pathway	T cells;B lineage;Myeloid dendritic cells;Neutrophils;En	T cells;B lineage;NK cells;Myeloid dendritic cells;Neutrophi
229233_at NRG3	214053_at ERBB4	0.85912910618793	4	GO:0001934|GO:0007169|GO:0014068|G	positive regulation of protein phosphorylation|transmembrane rec	T cells;B lineage;Myeloid dendritic cells;Neutrophils;En	T cells;B lineage;Myeloid dendritic cells;Neutrophils;Endoth
207576_x_at OXT	208260_at AVPR1B	0.703284950343774	4	GO:0007204	positive regulation of cytosolic calcium ion concentration	T cells;B lineage;Myeloid dendritic cells;Neutrophils;En	T cells;NK cells;Myeloid dendritic cells;Neutrophils;Endoth
237774_at PENK	207553_at OPRK1	0.61100076394194	4	GO:0007193|GO:0019233	adenylate cyclase-inhibiting G-protein coupled receptor signaling	Monocytic lineage	Endothelial cells;Fibroblasts
205479_s_at PLAU	210845_s_at PLAUR	0.771122994652406	4	GO:0007596	blood coagulation	T cells;B lineage;NK cells;Neutrophils;Endothelial cells;	T cells;B lineage;Myeloid dendritic cells;Neutrophils;Endoth
240033_at PLG	206429_at F2RL1	0.780349914417402	4	GO:0007204|GO:0007596|GO:0050900	positive regulation of cytosolic calcium ion concentration|blood c	Neutrophils	Monocytic lineage
205720_at POMC	217303_s_at ADRB3	0.513408206861655	4	GO:0007188|GO:0006898	adenylate cyclase-modulating G-protein coupled receptor signalin	T cells;B lineage;Neutrophils;Endothelial cells;Fibroblas	T cells;Cytotoxic lymphocytes
210670_at PPY	210729_at NPY2R	0.585377035859226	4	GO:0007193|GO:0007204	adenylate cyclase-inhibiting G-protein coupled receptor signaling	B lineage	Neutrophils;Endothelial cells
201433_s_at PTDSS1	215834_x_at SCARB1	0.502406602428905	4	GO:0006898	receptor-mediated endocytosis	Endothelial cells	T cells;B lineage;NK cells;Myeloid dendritic cells;Neutrophi
1555238_at PTH2	217303_s_at ADRB3	0.621084797555386	4	GO:0007188|GO:0006898	adenylate cyclase-modulating G-protein coupled receptor signalin	T cells;Myeloid dendritic cells;Neutrophils;Endothelial c	T cells;Cytotoxic lymphocytes
211253_x_at PYY	210729_at NPY2R	0.616806722689076	4	GO:0007193|GO:0007204	adenylate cyclase-inhibiting G-protein coupled receptor signaling	Monocytic lineage	Neutrophils;Endothelial cells
1553403_at RLN3	1552715_a_at RXFP1	0.682811306340718	4	GO:0007188	adenylate cyclase-modulating G-protein coupled receptor signalin	T cells;B lineage;NK cells;Myeloid dendritic cells;Neutr	T cells;B lineage;NK cells;Myeloid dendritic cells;Neutrophi
209875_s_at SPP1	227297_at ITGA9	0.59266615737204	4	GO:0030198	extracellular matrix organization	Neutrophils;Endothelial cells	Neutrophils;Endothelial cells
205016_at TGFA	202454_s_at ERBB3	0.834377387318564	4	GO:0007169|GO:0009968|GO:0014068|G	transmembrane receptor protein tyrosine kinase signaling pathway	Neutrophils;Fibroblasts	T cells;B lineage;NK cells;Myeloid dendritic cells;Neutrophi
205016_at TGFA	214053_at ERBB4	0.748204736440031	4	GO:0001934|GO:0007169|GO:0014068|G	positive regulation of protein phosphorylation|transmembrane rec	Neutrophils;Fibroblasts	T cells;B lineage;Myeloid dendritic cells;Neutrophils;Endoth

215775_at THBS1	215835_at SCARB1	0.700840336134454	4	GO:0006898|GO:0033344|GO:0042632|G	receptor-mediated endocytosis|cholesterol efflux|cholesterol home	Endothelial cells	T cells
214529_at TSHB	217303_s_at ADRB3	0.577081741787624	4	GO:0007188|GO:0006898	adenylate cyclase-modulating G-protein coupled receptor signalin	T cells;B lineage;Myeloid dendritic cells;Neutrophils;En	T cells;Cytotoxic lymphocytes
232674_at UCN2	207897_at CRHR2	0.506837804214318	4	GO:0007188	adenylate cyclase-modulating G-protein coupled receptor signalin	T cells;B lineage;NK cells;Myeloid dendritic cells;Neutr	T cells;Neutrophils;Endothelial cells;Fibroblasts
203868_s_at VCAM1	227297_at ITGA9	0.548051948051948	4	GO:0030198	extracellular matrix organization	T cells;B lineage;NK cells;Myeloid dendritic cells;Neutr	Neutrophils;Endothelial cells
204806_x_at HLA-F	207697_x_at LILRB2	0.537967914438503	4	GO:0002250|GO:0050776	adaptive immune response|regulation of immune response	B lineage;NK cells;Myeloid dendritic cells;Endothelial c	T cells;Myeloid dendritic cells;Neutrophils;Endothelial cells;

ls;Endothelial cells;Fibroblasts

ls;Endothelial cells;Fibroblasts

elial cells;Fibroblasts
lls;Fibroblasts

elial cells;Fibroblasts

lls;Neutrophils;Endothelial cells;Fibroblasts
lls;Neutrophils;Endothelial cells;Fibroblasts
ls;Endothelial cells;Fibroblasts

ls;Endothelial cells;Fibroblasts
elial cells;Fibroblasts

elial cells;Fibroblasts

elial cells;Fibroblasts
elial cells;Fibroblasts

elial cells;Fibroblasts

ls;Endothelial cells;Fibroblasts
ls;Endothelial cells;Fibroblasts

elial cells;Fibroblasts

ls;Endothelial cells;Fibroblasts

ls;Endothelial cells;Fibroblasts

;Fibroblasts
elial cells;Fibroblasts

;Fibroblasts
ıelial cells;Fibroblasts
›thelial cells
;Fibroblasts

;Fibroblasts

;Fibroblasts
ıelial cells;Fibroblasts

ıelial cells;Fibroblasts

;Fibroblasts
ıelial cells;Fibroblasts

ıelial cells;Fibroblasts
ıelial cells;Fibroblasts

ıelial cells;Fibroblasts

›thelial cells

lls;Endothelial cells;Fibroblasts
lls;Endothelial cells;Fibroblasts

ıelial cells;Fibroblasts

ıelial cells;Fibroblasts

roblasts

lls;Endothelial cells;Fibroblasts
lls;Endothelial cells;Fibroblasts
lls;Endothelial cells;Fibroblasts

ıelial cells;Fibroblasts

ıelial cells;Fibroblasts

;Fibroblasts

ls;Endothelial cells;Fibroblasts

othelial cells
ıelial cells;Fibroblasts

ls;Endothelial cells;Fibroblasts

ıelial cells;Fibroblasts

ıelial cells;Fibroblasts

ls;Endothelial cells;Fibroblasts
elial cells;Fibroblasts

ls;Endothelial cells;Fibroblasts
ls;Endothelial cells;Fibroblasts
ls;Endothelial cells;Fibroblasts
ıelial cells;Fibroblasts
ls;Endothelial cells;Fibroblasts
ıelial cells;Fibroblasts

ls;Endothelial cells;Fibroblasts

ıelial cells;Fibroblasts
ıelial cells;Fibroblasts
ıelial cells;Fibroblasts
ıelial cells;Fibroblasts
ıelial cells;Fibroblasts

ıelial cells;Fibroblasts
ıelial cells;Fibroblasts

ls;Endothelial cells;Fibroblasts
elial cells;Fibroblasts

ls;Endothelial cells;Fibroblasts

elial cells;Fibroblasts

elial cells;Fibroblasts

lasts

elial cells;Fibroblasts

lasts

elial cells;Fibroblasts

elial cells;Fibroblasts
lasts
elial cells;Fibroblasts
lasts

elial cells;Fibroblasts

lasts

elial cells;Fibroblasts

lasts

elial cells;Fibroblasts
lasts

elial cells;Fibroblasts

elial cells;Fibroblasts

lasts
elial cells;Fibroblasts

elial cells;Fibroblasts

elial cells;Fibroblasts

lasts

elial cells;Fibroblasts

elial cells;Fibroblasts

ls;Endothelial cells;Fibroblasts

ls;Endothelial cells;Fibroblasts

helial cells;Fibroblasts
roblasts
roblasts
othelial cells

;Fibroblasts
helial cells;Fibroblasts
;Fibroblasts
helial cells;Fibroblasts
helial cells;Fibroblasts

helial cells;Fibroblasts
ls;Endothelial cells;Fibroblasts
helial cells;Fibroblasts
helial cells;Fibroblasts
helial cells;Fibroblasts

othelial cells
ls;Endothelial cells;Fibroblasts
ls;Endothelial cells;Fibroblasts

helial cells
;Fibroblasts

helial cells;Fibroblasts

ls;Endothelial cells;Fibroblasts

helial cells;Fibroblasts

helial cells;Fibroblasts

helial cells;Fibroblasts

ls;Endothelial cells;Fibroblasts

lial cells;Fibroblasts

ls;Endothelial cells;Fibroblasts

helial cells;Fibroblasts

helial cells;Fibroblasts

helial cells;Fibroblasts

helial cells;Fibroblasts

helial cells;Fibroblasts

ls;Endothelial cells;Fibroblasts

helial cells;Fibroblasts

ls;Endothelial cells;Fibroblasts

helial cells;Fibroblasts

helial cells;Fibroblasts

helial cells;Fibroblasts

helial cells;Fibroblasts

roblasts

elial cells;Fibroblasts

;Fibroblasts

;Fibroblasts

elial cells;Fibroblasts

;Fibroblasts

elial cells;Fibroblasts

elial cells;Fibroblasts

elial cells;Fibroblasts

elial cells;Fibroblasts

elial cells;Fibroblasts

elial cells;Fibroblasts

elial cells;Fibroblasts

phils;Endothelial cells;Fibroblasts

elial cells;Fibroblasts

elial cells;Fibroblasts

ls;Endothelial cells;Fibroblasts

elial cells;Fibroblasts

elial cells;Fibroblasts

ls;Endothelial cells;Fibroblasts

;Fibroblasts

elial cells;Fibroblasts
ls;Endothelial cells;Fibroblasts

elial cells;Fibroblasts
phils;Endothelial cells;Fibroblasts
elial cells;Fibroblasts

elial cells;Fibroblasts

elial cells;Fibroblasts
elial cells;Fibroblasts

elial cells;Fibroblasts
elial cells;Fibroblasts

elial cells;Fibroblasts

;Fibroblasts

elial cells;Fibroblasts

elial cells;Fibroblasts

ls;Endothelial cells;Fibroblasts
;Fibroblasts

ls;Endothelial cells;Fibroblasts

ls;Endothelial cells;Fibroblasts

;Fibroblasts
elial cells;Fibroblasts
ls;Endothelial cells;Fibroblasts
elial cells;Fibroblasts

;Fibroblasts
elial cells;Fibroblasts

ls;Neutrophils;Endothelial cells;Fibroblasts

ıelial cells;Fibroblasts

lls;Endothelial cells;Fibroblasts
ıelial cells;Fibroblasts

ıelial cells;Fibroblasts

ıelial cells;Fibroblasts
;Fibroblasts

ıelial cells;Fibroblasts

lls;Endothelial cells;Fibroblasts
lls;Endothelial cells;Fibroblasts

lls;Endothelial cells;Fibroblasts

ells

ıelial cells;Fibroblasts

ıelial cells;Fibroblasts

elial cells;Fibroblasts
ıelial cells;Fibroblasts

helial cells;Fibroblasts

helial cells;Fibroblasts

lls;Endothelial cells;Fibroblasts
helial cells;Fibroblasts

helial cells;Fibroblasts

helial cells;Fibroblasts

helial cells;Fibroblasts

helial cells;Fibroblasts
lls;Endothelial cells;Fibroblasts

helial cells;Fibroblasts
helial cells;Fibroblasts
;Fibroblasts
;Fibroblasts
;Fibroblasts

lls;Endothelial cells;Fibroblasts
lls;Endothelial cells;Fibroblasts
helial cells;Fibroblasts
lls;Endothelial cells;Fibroblasts
helial cells;Fibroblasts

ells;Fibroblasts

lls;Endothelial cells;Fibroblasts
helial cells;Fibroblasts
elial cells;Fibroblasts

helial cells;Fibroblasts

lls;Endothelial cells;Fibroblasts

lls;Endothelial cells;Fibroblasts

lls;Endothelial cells;Fibroblasts
helial cells;Fibroblasts

;Fibroblasts

Table S8 L-R pairs selection

LR pairs	L	R	Specificity	PCNSL Reference (PMID)	OS (p-value <0,01)	RFS (p-value <0,01)
ADAM10-AXL	ADAM10	AXL	Specific		NO	YES
ADAM15-ITGA9	ADAM15	ITGA9	Specific		NO	NO
ADAM17-ITGB1	ADAM17	ITGB1	Specific		NO	NO
ADAM9-ITGA6	ADAM9	ITGA6	Specific		NO	YES
ADAM9-ITGAV	ADAM9	ITGAV	Specific		NO	YES
ANGPT1-TEK	ANGPT1	TEK	Specific		NO	YES
ANGPTL1-TEK	ANGPTL1	TEK	Specific		NO	NO
B2M-HLA-F	B2M	HLA-F	Specific		NO	NO
BCAN-EGFR	BCAN	EGFR	Specific		NO	NO
BMP8A-BMPR1B	BMP8A	BMPR1B	Specific		NO	NO
BTLA-CD79A	BTLA	CD79A	Specific		NO	NO
C3-C3AR1	C3	C3AR1	Specific		YES	YES
C3-CD81	C3	CD81	Specific		YES	YES
CALM1-MYLK	CALM1	MYLK	Specific		NO	NO
CALM1-PDE1A	CALM1	PDE1A	Specific		NO	NO
CALM1-PDE1C	CALM1	PDE1C	Specific		NO	NO
CCL4-CCR5	CCL4	CCR5	Specific	Venetz 2010 International Jounral of Cancer (20872671)	NO	NO
CCL5-CCR5	CCL5	CCR5	Specific	Venetz 2010 International Jounral of Cancer (20872671)	NO	NO
CCL8-CCR1	CCL8	CCR1	Specific		NO	NO
CD14-ITGB2	CD14	ITGB2	Specific		NO	NO
CDH1-PTPRF	CDH1	PTPRF	Specific	Rubenstein 2006 Blood (16418334)	NO	NO
COL1A1-CD44	COL1A1	CD44	Specific		NO	NO
COL1A2-ITGA1	COL1A2	ITGA1	Specific	Tun 2008 Blood (18184868)	NO	NO
COL1A2-ITGB3	COL1A2	ITGB3	Specific	Tun 2008 Blood (18184868)	NO	NO
COL4A5-ITGAV	COL4A5	ITGAV	Specific		NO	NO
COL7A1-ITGB1	COL7A1	ITGB1	Specific		NO	NO
COL8A1-ITGA1	COL8A1	ITGA1	Specific		NO	NO
CRH-CRHR2	CRH	CRHR2	Specific		NO	NO
CD86-CTLA-4	CD86	CTLA4	Specific		NO	NO
CXCL12-CD4	CXCL12	CD4	Specific		NO	YES
DLL1-NOTCH1	DLL1	NOTCH1	Specific		NO	NO
DLL1-NOTCH3	DLL1	NOTCH3	Specific		NO	NO
DLL4-NOTCH4	DLL4	NOTCH4	Specific		NO	NO
EDIL3-ITGAV	EDIL3	ITGAV	Specific		NO	NO
EDIL3-ITGB5	EDIL3	ITGB5	Specific		NO	NO
EFNB3-EPHB3	EFNB3	EPHB3	Specific	Rubenstein 2006 Blood (16418334)	NO	NO
EFNB3-EPHB4	EFNB3	EPHB4	Specific		NO	NO
EGF-EGFR	EGF	EGFR	Specific		NO	YES
EGF-LRP2	EGF	LRP2	Specific		NO	NO
FBLN1-ITGB1	FBLN1	ITGB1	Specific		NO	YES
FGB-ITGAM	FGB	ITGAM	Specific		NO	NO

FGF12-FGFR2	FGF12	FGFR2	Specific		NO	NO
FGF12-FGFR3	FGF12	FGFR3	Specific		NO	NO
FGF14-FGFR2	FGF14	FGFR2	Specific		NO	NO
FGF14-FGFR3	FGF14	FGFR3	Specific		NO	NO
FGF1-EGFR	FGF1	EGFR	Specific		NO	NO
FGF1-FGFR2	FGF1	FGFR2	Specific		NO	NO
FGF1-FGFR3	FGF1	FGFR3	Specific		NO	NO
FGF1-FGFRL1	FGF1	FGFRL1	Specific		NO	NO
FGF22-FGFR2	FGF22	FGFR2	Specific		NO	NO
FGF2-FGFR2	FGF2	FGFR2	Specific		NO	NO
FGF2-FGFR3	FGF2	FGFR3	Specific		NO	NO
FGF2-SDC2	FGF2	SDC2	Specific		NO	NO
FN1-COL13A1	FN1	COL13A1	Specific		NO	NO
FN1-ITGB3	FN1	ITGB3	Specific		NO	NO
GDF11-ACVR2B	GDF11	ACVR2B	Specific		NO	NO
GDF11-BMPR1B	GDF11	BMPR1B	Specific		NO	NO
GDNF-GFRA1	GDNF	GFRA1	Specific		NO	NO
HBEGF-EGFR	HBEGF	EGFR	Specific		NO	NO
HGF-ITGB1	HGF	ITGB1	Specific		NO	YES
HSPG2-COL13A1	HSPG2	COL13A1	Specific		NO	NO
ICAM1-ITGB2	ICAM1	ITGB2	Specific		NO	NO
IL10-IL10RB	IL10	IL10RB	Specific	Kishimoto 2016 Cancer Med (27709813)	NO	NO
JAG1-NOTCH2	JAG1	NOTCH2	Specific		NO	NO
KITLG-KIT	KITLG	KIT	Specific		NO	NO
LAMA1-SDC2	LAMA1	SDC2	Specific		NO	NO
LAMA4-ITGB1	LAMA4	ITGB1	Specific	Tun 2008 Blood (18184868)	NO	NO
LAMA5-ITGA6	LAMA5	ITGA6	Specific		NO	NO
LAMC3-ITGB1	LAMC3	ITGB1	Specific		NO	NO
LTBP1-ITGB5	LTBP1	ITGB5	Specific		NO	NO
LTBP3-ITGB5	LTBP3	ITGB5	Specific		NO	NO
LTF-LRP1	LTF	LRP1	Specific		YES	YES
MFGE8-ITGB3	MFGE8	ITGB3	Specific		NO	NO
MMP9-CD44	MMP9	CD44	Specific		NO	NO
NCAM1-FGFR2	NCAM1	FGFR2	Specific		NO	NO
NCAM1-GFRA1	NCAM1	GFRA1	Specific		NO	YES
NID1-ITGB1	NID1	ITGB1	Specific		NO	NO
NID1-ITGB3	NID1	ITGB3	Specific		NO	NO
NID2-COL13A1	NID2	COL13A1	Specific		NO	NO
PDGFB-LRP1	PDGFB	LRP1	Specific		NO	YES
PDGFB-PDGFRA	PDGFB	PDGFRA	Specific		YES	YES
PDGFC-FLT1	PDGFC	FLT1	Specific		NO	YES
PDGFC-PDGFRA	PDGFC	PDGFRA	Specific		NO	YES
PGF-FLT1	PGF	FLT1	Specific		NO	NO
PKM-CD44	PKM	CD44	Specific		NO	NO
PLAU-ITGB2	PLAU	ITGB2	Specific		NO	NO

PLTP-ABCA1	PLTP	ABCA1	Specific		NO	YES
PROS1-AXL	PROS1	AXL	Specific		NO	YES
PSAP-LRP1	PSAP	LRP1	Specific		YES	YES
PTN-PTPRS	PTN	PTPRS	Specific		NO	NO
PTN-SDC3	PTN	SDC3	Specific		NO	YES
SELPLG-ITGAM	SELPLG	ITGAM	Specific		NO	YES
SELPLG-ITGB2	SELPLG	ITGB2	Specific		YES	YES
SEMA6D-TREM2	SEMA6D	TREM2	Specific		NO	NO
SERPINA1-LRP1	SERPINA1	LRP1	Specific		YES	YES
SERPINE2-LRP1	SERPINE2	LRP1	Specific		NO	NO
SORBS1-ITGB5	SORBS1	ITGB5	Specific		NO	NO
SPP1-ITGAV	SPP1	ITGAV	Specific	Sung 2011 Blood (21088137);Tun 2008 Blood (18184868)	NO	NO
SPP1-ITGB5	SPP1	ITGB5	Specific	Sung 2011 Blood (21088137);Tun 2008 Blood (18184868)	NO	NO
TF-LRP2	TF	LRP2	Specific		NO	NO
TGFA-EGFR	TGFA	EGFR	Specific		NO	NO
TGFA-ERBB2	TGFA	ERBB2	Specific		NO	NO
TGFB1-ITGB3	TGFB1	ITGB3	Specific		NO	NO
TGFB3-ITGB5	TGFB3	ITGB5	Specific		NO	NO
TGM2-ITGB3	TGM2	ITGB3	Specific		NO	NO
THBS1-CD36	THBS1	CD36	Specific		NO	NO
THBS1-ITGB1	THBS1	ITGB1	Specific		NO	NO
THBS1-ITGB3	THBS1	ITGB3	Specific		NO	NO
TLN1-ITGB3	TLN1	ITGB3	Specific		NO	NO
TNFSF13-SDC2	TNFSF13	SDC2	Specific		NO	NO
TNFSF13-TNFRSF1A	TNFSF13	TNFRSF1A	Specific	Tun 2008 Blood (18184868); Mulazzani 2019 Journal of Hematology and Oncology (31615554)	NO	YES
TNFSF9-TRAF2	TNFSF9	TRAF2	Specific		NO	NO
VCAM1-ITGB2	VCAM1	ITGB2	Specific	Montesinos-Rongen 2008 Leukemia (17989719)	NO	NO
VCAN-EGFR	VCAN	EGFR	Specific		NO	NO
VCAN-ITGB1	VCAN	ITGB1	Specific		NO	YES
FADD-FAS	FADD	FAS	Specific	Rubenstein 2006 Blood (16418334)	NO	NO
ICAM1-SPN	ICAM1	SPN	Specific		NO	NO
B2M-CD247	B2M	CD247	Specific		NO	NO
EGF-ERBB4	EGF	ERBB4	Specific		NO	NO
HLA-A-LILRB2	HLA-A	LILRB2	Specific		NO	NO
HLA-C-LILRB2	HLA-C	LILRB2	Specific		NO	NO
LAMA1-ITGB8	LAMA1	ITGB8	Specific		YES	YES
LGALS9-HAVCR2	LGALS9	HAVCR2	Specific		NO	NO
PLAU-PLAUR	PLAU	PLAUR	Specific		NO	NO
SPP1-ITGA9	SPP1	ITGA9	Specific	2011 Blood (21088137);Tun 2008 Blood (1818	NO	NO

TGFA-ERBB3	TGFA	ERBB3	Specific	NO	NO
TGFA-ERBB4	TGFA	ERBB4	Specific	NO	NO
HLA-F-LILRB2	HLA-F	LILRB2	Specific	NO	NO
